# ChAdOx1 nCoV-19 Vaccine and Thrombosis with Thrombocytopenia Syndrome among Adults: A Systematic Review

**DOI:** 10.34172/apb.2023.081

**Published:** 2023-04-29

**Authors:** Homa Faghihi, Negar Mottaghi-Dastjerdi, Mohammad Sharifzadeh, Nader Rahimi Kakavandi

**Affiliations:** ^1^Department of Pharmaceutics and Pharmaceutical Nanotechnology, School of Pharmacy Iran University of Medical Sciences, Tehran, Iran.; ^2^Department of Pharmacognosy and Pharmaceutical Biotechnology, School of Pharmacy, Iran University of Medical Sciences, Tehran, Iran.; ^3^Department of Pharmacology and Toxicology, Faculty of Pharmacy, Toxicology and Poisoning Research Centre, Tehran University of Medical Sciences, Tehran, Iran.; ^4^Department of Toxicology & Pharmacology, Faculty of Pharmacy, Tehran University of Medical Sciences, Tehran, Iran.; ^5^Health and Environment Research Center, Ilam University of Medical Sciences, Ilam, Iran.

**Keywords:** Cerebral venous sinus thrombosis, Cerebral venous thrombosis, ChAdOx1 nCov-19 vaccine, Oxford AstraZeneca COVID-19 vaccine, Thrombotic thrombocytopenia syndrome

## Abstract

Several vaccine-induced thrombotic thrombocytopenia syndrome (VITTS) cases have been reported after the ChAdOx1 nCov-19 vaccination. The current study systematically reviewed the reported post-ChAdOx1 nCoV-19 vaccination thrombotic thrombocytopenia cases. Their laboratory and clinical features, as well as the diagnostic and therapeutic measures, were investigated. Online databases were searched until 25 August 2021. Studies reporting post-ChAdOx1 nCov-19 vaccination thrombotic thrombocytopenia syndrome (TTS) were included. Overall, 167 cases (21-77 years old) from 53 publications were included showing a female dominance of 1.75 times. About 85% of the cases exhibited the primary symptoms within the first two weeks post-vaccination. Headache was the most common initial symptom (>44.2%), and hemorrhage/thrombotic problems (22.46%), as well as discoordination/weakness/numbness/ hemiparesis/cyanotic toes (19.6%), were the most prevalent uncommon initial symptoms. Prothrombin time (PT), D-dimers, and C-reactive protein were the most remarkable increased laboratory parameters in 50.6%, 99.1%, and 55.6% of cases, respectively. In comparison, platelet and fibrinogen were the most remarkable decreased laboratory parameters in 92.7% and 50.5% of cases, respectively. Most VITT cases presented with cerebral venous thrombosis/cerebral venous sinus thrombosis, supraventricular tachycardia, transverse sinus/cerebral thrombosis, pulmonary embolism, and cerebral hemorrhage. Anti-PF4 antibody measurement through immunoassays and functional assays were positive in 86.2% and 73% of cases, respectively. About 31% of the cases died. Early diagnosis and proper therapeutic measures are important in ChAdOx1 nCov-19 vaccine-induced VITTS patients. Therefore, experts are recommended to know the corresponding clinical and laboratory features, as well as diagnostic methods. Elucidation of the pathophysiologic mechanism of ChAdOx1 nCov-19 vaccine-induced TTS deserves further investigation.

## Introduction

 Vaccine-induced thrombotic thrombocytopenia (VITT) is a severe adverse event upon vaccination associated with extraordinary thrombosis and a concurrent decrease in platelet counts. VITT is likewise known as vaccine-induced prothrombotic immune thrombocytopenia and/or thrombosis with thrombocytopenia syndrome (TTS).^1^ ChAdOx1 nCoV-19 (Oxford/AstraZeneca) and Janssen COVID-19 vaccines, as adenoviral vector-based vaccines, have been implicated in creating VITT. The probable explanation for such phenomenon is that the free existing DNA in these vaccines might bind to anti-platelet factor 4 (PF4) antibodies.^2^ VITT is mainly attributed to PF4 antibodies partially like heparin-induced thrombocytopenia (HIT) regarding clinical and biochemical aspects. These immunoglobulin G class antibodies activate platelets via FcɣRIIa receptors, causing them to clump together, leading to clot formation and thrombocytopenia.^3^

 About a hundred cases of thrombosis at atypical sites such as cerebral sinus, splanchnic veins, and the right ventricle with variable degrees of thrombocytopenia have been reported 5 to 30 days upon vaccination with Oxford/AstraZeneca and Janssen COVID-19 vaccines. Microvascular events in the brain, the lungs, and the kidneys have been additionally observed.^4^ The precise incidence of VITT after vaccination against COVID-19 remains ambiguous due to insufficient clinical experiences, complicated diagnostic methods, several feasibly-involved mechanisms, and lack of well-defined periods for follow-up.^5,6^ Based on data latest updated in November 2021 offered by Uptodate.com, the highest incidence rates following Oxford/AstraZeneca and Janssen vaccine were 1 in 26 000 and 1 in 533 333, respectively. While crucial risk factors for VITT have not been comprehensively known, young females are proposed as the most vulnerable groups to such an adverse event. Unfortunately, patients with VITT often exhibit intravascular coagulation combined with thrombocytopenia without noticeable clinical symptoms until the immediate onset of thrombosis.^7^

 Infection with SARS-CoV-2 can cause the systemic release of viral RNA leading to activation of the innate immune coagulation pathway associated with systemic and pulmonary immunothrombosis. Recently, COVID-19 viral vectored vaccines such as the ChAdOx1 nCoV-19 vaccine are associated with thrombotic thrombocytopenia after vaccination called VITT.^3^ One of the main mechanisms clarified by the Greifswald Working Group with Andreas Greinacher leadership was antibody formation against platelet antigens (anti-PF4) due to the stimulation of the immune system and inflammatory reactions. These antibodies can finally lead to an extensive activation of the platelets via the Fc receptor, which resembles HIT.^8^ After intramuscular administration of an adenoviral-vectored vaccine, a cascade of events occurs, including microvascular damage, microbleeding and activation of the platelets with the release of PF4 and disperse of the adenovirus cargo with the engagement of DNA-PF4 can interrupt the immune tolerance causing rare autoimmunity directed by PF4.^3^ According to the reported deaths associated with ChAdOx1 nCoV-19 post-vaccination VITT, early diagnosis and fast therapeutic measures could benefit the outcome of the patients.

 In this study, we systematically reviewed the reported cases of post-vaccination thrombotic thrombocytopenia contributed to the ChAdOx1 nCoV-19 vaccine and investigated their laboratory and clinical features and the diagnostic and therapeutic measures applied in these cases.

## Methods

 This study was performed based on the PRISMA (Preferred Reporting Items for Systematic Reviews and Meta-Analyses) protocol for reporting systematic reviews and meta-analyses.

###  Search strategy

 We performed a comprehensive literature search in the online databases of PubMed, Scopus, and Google Scholar up to August 25th, 2021. In the investigation, we purposed to identify case reports investigating the effects of ChAdOx1 nCoV-19 vaccination on vaccine-induced immune thrombotic thrombocytopenia in adults. The following keywords were used in the search strategy: (Thrombosis OR Thromboses OR Thrombus OR “Blood Clot” OR “Blood Clots” OR “Clot, Blood” OR “Clots, Blood” OR Thrombocytopenia OR “cerebral venous sinus thrombosis (CVST )” OR “Vaccine-induced immune thrombotic thrombocytopenia (VITT)” OR “vaccine-induced prothrombotic immune thrombocytopenia (VIPIT)” OR Platelets OR “Blood Platelet” OR “Platelet, Blood” OR “Platelets, Blood” OR Thrombocytes OR thrombocyte OR platelets OR platelet OR “low-platelet syndrome” OR Thrombocytopenias OR thrombopenia OR thrombopenias) AND (AstraZeneca OR “ChAdOx1 SARS2 vaccine” OR “ChAdOx1 nCoV-19 “ OR Covishield OR AZD1222 OR “Oxford AstraZeneca COVID-19 vaccine” OR “ChAdOx1 COVID-19 vaccine” OR “COVID-19 vaccine” OR “SARS-CoV-2 vaccine”). No time restriction was applied. References of the relevant publications were manually screened to avoid missing any eligible studies. Unpublished researches were not included. Two independent investigators conducted a literature search.

###  Inclusion criteria

 We included eligible studies that met the following criteria: 1) case reports, 2) studies that administered ChAdOx1 nCoV-19, 3) case reports and case series with thrombocytopenia and thrombosis after ChAdOx1 nCoV-19 administration. The complete one was included if > 1 article was published for one dataset.

###  Exclusion criteria

 In the current systematic review, we excluded experimental studies, those with a cohort, cross-sectional, and case-control design, clinical trials, and review articles. We also excluded studies with reports on thrombosis without thrombocytopenia or thrombocytopenia without thrombosis after ChAdOx1 nCoV-19 vaccine administration.

###  Data extraction

 Two independent investigators performed data extraction from each eligible case report and case series. The following information was extracted: name of the first author, publication year, individuals’ characteristics (age, sex, ethnicity, smoker/non-smoker), place of measure, number of cases in each study, first dose of ChAdOx1 nCoV-19 vaccine, medical history, previous exposure to heparin, medication on admission, chief complaint/initial symptom, onset of symptom (days) post vaccination (initial symptoms/thrombocytopenia/thrombotic complications), therapeutic measures, medical examination/imaging method/site of thrombosis/hemorrhages, outcome, laboratory parameters including hemoglobin, platelets/white/red blood cells count, prothrombin time (PT)/ international normalized ratio (INR), activated partial thromboplastin time (ratio)/aPTT/PTT, thrombin time, fibrinogen, D-dimers, antithrombin, C-reactive protein, haptoglobin, neutrophil, lymphocyte, monocyte, eosinophil, aminotransferase, Gamma glutamyl transferase, sodium, potassium, calcium, glucose, nitrogen urea, lactate dehydrogenase, lactate, total bilirubin, amylase, coagulation factor, folic acid, vitamin B12, pro-calcitonin, creatinine, total protein, albumin, paroxysmal nocturnal haemoglobinuria, ferritin, interleukin, cholesterol/ high-density lipoprotein ratio, urea, EXTEM CT, EXTEM A10, FIBTEM A10, INTEM CT, fibrin degradation product, protein C activity, protein S (free antigen), VWF, thrombophilia mutations (factor V Leiden, prothrombin G20210A, MTHFR, Janus kinase 2 (JAK2)), antiphospholipid antibodies, anti-thyroid peroxidase, myeloperoxidase, anti-neutrophil cytoplasmic anti-bodies, rheumatoid factor, antinuclear antibodies, extractable nuclear antigen, dsDNA, anti-globulin test, neutrophil DNA extracellular traps, schistocytes on peripheral blood smear, blood film, immunoglobulins IgA/IgM/IgG, complement, cryoglobulins, ADAMTS13, antiplatelet antibodies, homocysteine, septic screen of blood/urine/respiratory cultures, acute kidney injury, SARS-CoV-2 screening, test for other possible causes of thrombocytopenia including hepatitis B virus, hepatitis C virus, HIV, Epstein–Barr virus, cytomegalovirus, hantaviruses, and Helicobacter pylori infections, as well as anti-PF4 antibodies screening (immunoassays and functional assays). If data on laboratory parameters were reported in different units, we converted them to the most frequently used unit.

## Results and Discussion

 Many regulatory agencies approve the Oxford-AstraZeneca COVID-19 vaccine with the viral vector platform for the prevention of COVID-19. Also called Vaxzevria, Covishield, AZD1222, ChAdOx1 SARS2 vaccine, and ChAdOx1 nCov-19, this vaccine has an efficacy of 66.7% two weeks after the second dose.^9^ Common side effects of the ChAdOx1 nCoV-19 vaccine include site reaction (tenderness, pain, warmth, itching or bruising), chills or feverish, headache, nausea, fatigue, unwell feeling, and joint pain, or muscle ache; all be resolved within a few days.^10^ However, there were few reports on rare cases of thrombosis at unusual sites associated with thrombocytopenia shortly after ChAdOx1 nCov-19 vaccine administration.^2,11^ Accordingly, in this systematic review, we investigated different features of the studies reporting the cases with thrombotic thrombocytopenia syndrome (TTS) after the ChAdOx1 nCov-19 vaccine administration.

**Figure 1:**
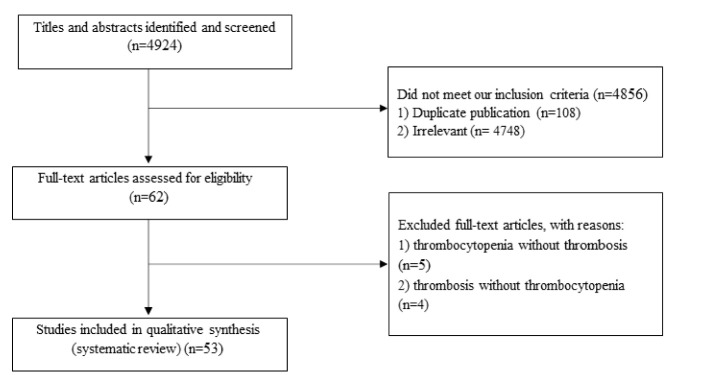
Flow diagram of study selection

 Totally, 4924 publications were identified in our initial search. After the screening, 4856 unrelated articles were excluded based on duplication (n = 108), title, and abstract assessment. Then, 62 publications remained for further evaluation of a full text. Out of these 62 eligible publications, five studies were also excluded due to the absence of thrombosis,^12-16^ and four studies were excluded due to the lack of thrombocytopenia.^17-20^ The flow diagram of study selection is outlined in Figure 1.

###  Characteristics of the included studies

 The characteristics of 53 case reports included in the current systematic review are illustrated in Table 1.One hundred and sixty-seven cases are included in the 53 studies in this systematic review (63.6% females and 36.4% males). These cases were between 21 to 77 years old (19.6% (20-29 years old), 20.9% (30-39 years old), 21.5% (40-49 years old), 15.3% (50-59 y/o), 16.6% (60-69 years old), and 6.1% (70-77 years old)). The primary symptoms after ChAdOx1 nCov-19 vaccination initiated from the first day ranged from 0-26 days after vaccination. However, these cases’ thrombocytopenia/thrombotic complications started from day 2 to 35 days after vaccination with ChAdOx1 nCov-19.

**Table 1:** Summary of case reports on VITT after ChAdOx1 nCov-19 vaccination

**First Author**	**Year**	**No. of cases**	**Onset of primary side effects post vaccination, days**	**Onset of symptoms (thrombocytopenia /thrombotic** **complications) post vaccination, days**	**Age (y)**	**Sex**	**Place of measure**	**Ethnicity**	**Ref.**
Greinacher	2021	9	4-16	4-16	22-49	8 F, 1 M	Germany	NA	^11^
Al-Mayhani	2021	3	6-21	11-21	35-43	2 F, 1 M	UK	2 Asian 1 White	^21^
Scully	2021	23	NA	6-24 (22 out of 23)	21-77	14 F, 9 M	UK	NA	^22^
Schultz	2021	5	7-10	7-10	32-54	4 F, 1 M	Norway	NA	^23^
Greinacher	2021	11	NA	5-16	22-49	9 F, 2 M	Germany and Austria	NA	^2^
Bayas	2021	1	0	8	55	1 F	Germany	NA	^24^
Blauenfeldt	2021	1	< 7	7	60	1 F	Denmark	Danish	^25^
Mehta	2021	2	6-9	6-9	25-32	2 M	UK	White	^26^
Castelli	2021	1	7	7	50	1 M	Italy	Caucasian	^27^
D’Agostino	2021	1	12	12	54	1 F	Italy	NA	^28^
Abu Esba	2021	2	14	> 14-16	40-61	1 F, 1 M	Saudi Arabia	NA	^29^
Franchini	2021	1	7	11	50	1 M	Italy	White	^30^
Garnier	2021	1	0	8	26	1 F	France	NA	^31^
Geeraerts	2021	2	NA	NA	NA	NA	France	NA	^32^
Gras-Champel	2021	27	NA	2-35	21-74	13 F, 14 M	France	NA	^33^
Jones	2021	1	20	25	63	1 M	UK	NA	^34^
Ramdeny	2021	1	14	21	54	1 M	UK	NA	^35^
Wolf	2021	3	0-8	7-17	22-46	3 F	Germany	NA	^36^
Xie	2021	1	< 7	7	23	1 F	UK	NA	^37^
Muster	2021	1	8	8	51	1 F	Austria	NA	^38^
Aladdin	2021	1	14	14	36	1 F	Saudi Arabia	NA	^39^
Althaus	2021	8	6-20	6-20	41.5 (24-53)	5 F, 3 M	Germany	NA	^40^
Bano	2021	3	8-13	10-16	53-61	2 F 1 M	UK	NA	^41^
Bonato	2021	1	14	14	26	1 F	Italy	Italian	^42^
Bourguignon	2021	3	7-18	15-24	63-72	1 F, 2 M	Canada	NA	^43^
Choi	2021	1	0	12	33	1 M	Korea	Korean	^44^
Cliff-Patel	2021	3	8-21	8-28	28-61	3 M	UK	NA	^45^
Gangi	2021	6	3-26	9-31	38 (26-48)	4 M, 2 F	UK	NA	^46^
Gattringer	2021	2	6-8	8-12	24-39	2 F	Austria	NA	^47^
Gessler	2021	2	7-12	7-12	47-50	2 F	Germany	NA	^48^
Graf	2021	1	9	14	29	1 M	Germany	NA	^49^
Guan	2021	1	5	10	52	1 M	Taiwan	East Asian	^50^
Huang	2021	1	0	10	34	1 M	Taiwan	Asian	^51^
Ikenberg	2021	1	0	7	early 30s	1 F	Germany	NA	^52^
Jacob	2021	1	0	7	39	1 F	UK	NA	^53^
Khuhapinant	2021	1	3	8	26	1 F	Thailand	Asian	^54^
Mauriello	2021	1	1	18	48	1 F	Italy	Caucasian	^55^
De Michele	2021	2	7-9	9-10	55-57	2 F	Italy	NA	^56^
Panovska-Stavridis	2021	1	9	10	29	1 F	Macedonia	Caucasian	^57^
Patriquin	2021	3	10-16	10-16	45-48	3 F	Canada	NA	^58^
Paul	2021	1	5	5	middle aged	1 M	India	NA	^59^
Ramessur	2021	1	0	14	73	1 M	UK	NA	^60^
Soleimani	2021	3	4-11	10-14	34- 59	1 M, 2 F	UK	NA	^61^
Tølbøll Sørensen	2021	1	8	11	30	1 F	Denmark	NA	^62^
Suresh	2021	1	2	12	27	1 M	UK	NA	^63^
Tejpal	2021	1	20	20	61	1 M	Canada	NA	^64^
Tiede	2021	5	5-11	6-15	41-67	5 F	Germany	NA	^65^
Turi	2021	1	2	6	57	1 F	Italy	NA	^66^
Varona	2021	1	10	11	47	1 M	Spain	NA	^67^
Vayne	2021	9	9-18	9-18	44 (21-69)	2 M, 7 F	Singapore	NA	^68^
Wang	2021	1	0	7	41	1 F	Taiwan	NA	^69^
Wiedmann	2021	1	7	10	34	1 F	Norway	NA	^70^
Zanferrari	2021	1	0	7	40	1 F	Italy	NA	^71^

###  Findings from the systematic review

####  Patient characteristics, site of thrombosis, and outcome of case reports on VITT after ChAdOx1 nCov-19 vaccination

 Our results showed that TTS occurs 1.75 times more in women. Most cases (62%) aged between 20-49 years old. Among the cases with TTS in this systematic review, about 60% had no remarkable PMH of any personal or familial disease/risk factors, and about 24% had pre-existing conditions. In addition, less than 30% of the cases had several regular medications with contraceptives as the most used medication.^22,23,33,40,42,46,62,70^ However, the number of cases for each condition or medication is not enough to find a relationship between the pre-existing condition or regular medication and TTS risk post-ChAdOx1 nCoV-19 vaccination.

 In addition to the common symptoms associated with administration of ChAdOx1 nCoV-19 vaccine with headache as the most common initial symptom ( > 44.2%),^2,11,21-23,25-27,29-31,35,36,39,41-43,45-55,57,58,60-63,65,66,69-71^ cases in this systematic review reported having some uncommon initial symptoms, including hemorrhage/thrombotic problems (22.46%)^11,22,40,58,67,68^ and discoordination/weakness/numbness/hemiparesis/cyanotic toes (19.6%)^21,23,25,26,34,36,39,41-45,53,58,61,65,70^ as the most prevalent uncommon initial symptoms which could be a signal alert to be aware of vaccine-induced thrombotic thrombocytopenia syndrome (VITTS). However, further study is needed to figure out a relationship between these initial symptoms and the risk of VITTS.

 The initial symptoms were appeared within the first, second, third, fourth, and fifth week post vaccination with ChAdOx1 nCov-19 vaccine in 32.1%,^2,11,21-27,30,31,33,36,37,40,43,44,46-48,50-56,59-61,63,65,66,69-71^ 53.3%,^2,11,21-23,26,28,29,33,35,36,38-43,45-49,56-58,61,62,65,67,68^ 11.5%,^2,11,22,33,34,40,43,45,58,64,68^ 1.8%,^22,33,46^ and 1.2%^33^ of the cases, respectively. These results showed that the initial symptoms after ChAdOx1 nCov-19 vaccination might start from the first day ranging from 0-26 days after vaccination. However, the thrombocytopenia/thrombotic complications in these cases started from day 2 to 35 days after vaccination with ChAdOx1 nCov-19. Our results confirmed that more than 85% of the cases showed the initial symptoms within the first- and second-week post-vaccination.

 This systematic review also investigated the clinical features in a cohort of cases presenting with acute atypical thrombosis, mainly involving the cerebral veins and concurrent thrombocytopenia. Most cases involved in this systematic review presented with cerebral venous thrombosis/cerebral venous sinus thrombosis ^2,11,21-23,29,30,32,33,35,40,41,44,46,50,52,61,62,65,67-69^, supraventricular tachycardia (thrombosis of the portal, mesenteric, splenic, ileal, or hepatic veins),^2,11,21-23,26,28-33,35,37,39-41,43,44,46,49,50,52,56,58,61-63,65-67,69^ transverse sinus/cerebral thrombosis^21,23,27-31,36,39,41-44,46,47,49,50,52,55,58,61,65,70^ and pulmonary embolism,^2,11,21-23,31,34,37,38,40,41,43,45,46,56,58,61,64,66-69^ as well as cerebral hemorrhage (occipital, temporo-occipital, frontal, juxtacortical, cerebellar, intraparenchymal/hemispheric/parenchymal hemorrhage/subarachnoid hemorrhage).^2,22,23,26-28,30,36,41,42,44,46,47,49,52,55,61,63,70,71^ However, further study is needed to make a reliable conclusion on the relationship between the outcome of the cases and ChAdOx1 nCov-19 vaccine-induced TTS.

 About 31.4% of the reported cases died^2,11,21-23,25-28,30,32,33,39-41,44,48,55,56,63,70^ and about 68.6% of the cases fully recovered.^2,11,21-24,29,31,33-38,40-43,45-47,49-54,56-62,64,65,67,69,71^ Details on patient characteristics, therapeutic measures, site of thrombosis, and outcome of case reports on VITT after ChAdOx1 nCov-19 vaccination are illustrated in Table S1a and summarized in Table S1b (see Supplementary fie 1).

###  Laboratory tests

 Common laboratory parameters including hemoglobin, WBC, platelet, PT/INR, aPTT, fibrinogen, thrombin, D-dimers, C-reactive protein, and antithrombin were also assessed in the included cases with ChAdOx1 nCov-19 vaccine-induced TTS in our systematic review. Summary of these laboratory blood tests are illustrated in Table 2 and the details are illustrated in Table S2. Our results showed normal levels of antithrombin in all cases, high levels of WBC, PT/INR, aPTT, fibrinogen, thrombin, D-dimers, C-reactive protein in 33.3%, 50.6%, 20.5%, 4.4%, 14.3%, 99.1%, and 55.6% of cases, respectively as well as lower than normal levels of hemoglobin, WBC, platelet, PT, aPTT and fibrinogen in 22.2%, 13.3%, 92.7%, 1.3%, 11.5%, and 50.5% of cases, respectively. PT, D-dimers, and C-reactive protein were the most remarkable increased laboratory parameters in 50.6%, 99.1%, and 55.6% of cases, respectively. In comparison, platelet and fibrinogen were the most remarkable decreased laboratory parameters in 92.7% and 50.5% of cases, respectively. Accordingly, these laboratory parameters should be considered most. In addition, abnormally increased levels of CRP are correlated with worse prospects and more rates of mortality in patients with COVID-19.^72^ However, further studies are needed to find a reliable relationship between these common laboratory parameters and ChAdOx1 nCov-19 vaccine-induced TTS.

**Table 2:** Summary of common laboratory blood tests and SARS-CoV-2 screening of case reports on VITT after ChAdOx1 nCov-19 vaccination

**Hemoglobin (n=27)**	**White cell (n=15)**	**Platelets** **(n=164)**	**PT/INR** **(n=79)**	**Activated partial thromboplastin time (ratio)/aPTT/PTT** **(n=78)**	**Thrombin time** **(n=8)**	**Fibrinogen** **(n=91)**	**D-dimers** **(n=111)**	**Antithrombin** **(n=12)**	**C-reactive protein** **(n=27)**	**SARS-CoV-2 screening** **(n=80)**
Normal: 77.8%	Normal: 53.3%	Normal: 7.3%	Normal: 48.1%	Normal: 67.9%	Normal: 75%	Normal: 45.1%	Normal: 0.9%	Normal: 100%	Normal: 44.4%	Negative: 98.75%
Low: 22.2%	High: 33.3%	Thrombocytopenia: 92.7%	High: 50.6%	High: 20.5%	High: 25%	High: 4.4%	High: 99.1%		High: 55.6%	Positive: 1.25%
	Low: 13.3%		Low: 1.3%	Low: 11.5%		Low: 50.5%				

 Less common laboratory tests of case reports on VITT after ChAdOx1 nCov-19 vaccination are summarized in Table 3, and the details are illustrated in Table S3.

**Table 3:** Summary of less common laboratory tests of case reports on VITT after ChAdOx1 nCov-19 vaccination

**Laboratory parameters**	**Results**
Haptoglobin (n = 3)	Normal: 100%
RBC (n = 5)	Normal: 80%; Low: 20%
Neutrophil (n = 8)	Normal: 37.5%; High: 62.5%
Lymphocyte (n = 5)	Normal: 60%; Low: 40%
Monocyte (n = 5)	Normal: 80%; Low: 20%
Eosinophil (n = 5)	Normal: 100%
Basophil (n = 2)	Normal: 100%
Aspartate aminotransferase (n = 6)	AST
Alanine aminotransferase (n = 8)	Normal: 100%
	ALT
	Normal: 37.5%; High: 62.5%
Gamma glutamyl transferase (n = 4)	Normal: 25%; High: 75%
Alkaline phosphatase (n = 3)	Normal: 100%
Sodium; Potassium (n = 2)	Normal: 100%
Nitrogen urea (n = 2)	Normal: 100%
Lactate dehydrogenase (n = 12)	Normal: 41%; High: 59%
Lactate (n = 3)	Normal: 100%
Total bilirubin (n = 10)	Normal: 70%; High: 30%
Clotting factors (n = 9)	Normal: 55%; Low:33%; High: 11%
Folic acid (n = 2)	Low:100%
Vitamin B12 (n = 1)	Normal: 100%
Procalcitonin (n = 1)	Normal: 100%
Creatinine (n = 13)	Normal: 85%; High: 15%
Total protein; Albumin (n = 1)	Normal: 100%
Paroxysmal nocturnal hemoglobinuria (n = 3)	Normal: 100%
Septic screening of blood, urine, and respiratory cultures (n = 1)	Normal: 100%
Acute kidney injury (n = 2)	Normal: 50%; High: 50%
Ferritin (n = 2)	Normal: 100%
Cholesterol high-density lipoprotein ratio (n = 2)	Normal: 50%; High: 50%
Urea (n = 1)	Normal: 100%

 Summary of thrombophilia marker assays in case reports on VITT after ChAdOx1 nCov-19 vaccination is illustrated in Table 4. Antiphospholipid antibodies (lupus anticoagulant, beta-2-glycoprotein 1, and anticardiolipin antibodies) as the most commonly evaluated thrombophilia markers were evaluated in a couple of cases as follows^2,11,22-26,30,32,34,36,37,41,42,47,53-57,61,62,65,66,71^: Lupus anticoagulant measured in 20.3% of cases (positive in 8 cases, negative in 26 cases), beta-2-glycoprotein was evaluated in 20.3% of cases (positive in 2 cases, negative in 32 cases), and anticardiolipin antibodies were measured in 21.5% of cases (positive in 4 cases, negative in 32 cases). Details of thrombophilia marker assays in case reports on VITT after ChAdOx1 nCov-19 vaccination is illustrated in Table S4.Coagulopathy has been found in association with the detection of antiphospholipid antibodies, but the latter’s relation with VITT remains controversial. Although the levels of these markers have been measured in about 20% of all-studied cases, the outcome did not provide significant evidence of antiphospholipid antibody linkages to the occurrence of VITT post-vaccination. It should be noted that there were no complete details of such screening in all cases, and therefore, the pathogenesis of these antibodies in VITT deserves further attention. Von Willebrand factor (VWF) was measured in 8 cases which were positive in 73%. In a previously performed research work, VWF and P-selectin were introduced as key factors which were involved in the formation of platelet–leukocyte complex, leading to platelet activation and enhanced thrombocytopenia upon adenovirus exposure which highlights the further assessments of this marker to elucidate its possible role in the pathogenesis of VITT. ^73^

**Table 4:** Summary of thrombophilia marker assays in case reports on VITT after ChAdOx1 nCov-19 vaccination

**Thrombophilia marker assays**	**Results**
EXTEM CT^a^ (n = 1)	High: 100%
EXTEM A10^b^ (n = 1)	High: 100%
FIBTEM A10^c^ (n = 1)	Normal: 100%
INTEM CT^d^ (n = 1)	High: 100%
Fibrin degradation product (n = 1)	High: 100%
Protein C activity (n = 19)	High: 5.26%; Normal: 94.74%
Protein S, free antigen (n = 19)	Normal: 100%
VWF (n = 8)	Normal: 12.5%; High: 87.5%
Thrombophilia mutations (Factor V Leiden, prothrombin G20210A, MTHFR, JAK2)	- Generally mentioned as Thrombophilia (n = 32)
	Normal: 100%
	- Factor V Leiden (n = 12)
	Normal: 33%; High: 67%
	- Prothrombin (G20210A) (n = 7)
	Normal: 43%; High: 57%
	- MTHFR (n = 2)
	High: 100%
	- JAK2 (n = 4)
	Normal: 100%
Antiphospholipid antibodies	- Lupus anticoagulant (n = 34)
	Normal: 76.5%; High: 23.5%
	- Beta-2-glycoprotein (n = 34)
	Normal: 94%; High: 6%
	- Anticardiolipin antibodies (n = 36)
	Normal: 88.8%; High: 11.2%
Anti-thyroid peroxidase, myeloperoxidase, anti-neutrophil cytoplasmic anti-bodies, rheumatoid factor, and antinuclear antibodies, extractable nuclear antigen, dsDNA, Anti-globulin test	- Antinuclear antibodies (n = 31)
	Normal: 100%
	- Extractable nuclear antigen (n = 26)
	Normal: 96.16%; High: 3.84%
	- Myeloperoxidase (n = 2)
	Normal: 50%; High: 50%
	- Proteinase 3 antineutrophil cytoplasmic antibody (n = 1)
	High: 100%
	- Anti–double-stranded DNA antibody (n = 2)
	Normal: 50%; High: 50%
	- Antineutrophilic antibodies (n = 2)
	Normal: 100%
	- Anti-globulin (n = 1)
	Normal: 100%
Neutrophil DNA extracellular traps (n = 1)	High: 100%
Schistocytes (n = 11)	Normal: 18%; High: 82%
Blood film (n = 16)	Normal: 20%
	- Thrombocytopenia without cell fragments: 20%
	- Leukocytosis of neutrophils with polychromasia, anisocytosis, and moderate thrombocytopenia: 6.66%
	- No hemolysis or fragments of red cell: 40%
	- Giant platelets: 6.66%
	- Platelet anisocytosis: 6.66%
Immunoglobulins (n = 3)	Normal: 67%; High: 33%
Complement (n = 11)	Normal: 72.7%; High: 9.1%; Low: 18.2%
Cryoglobulins (n = 1)	Normal: 100%
ADAMTS13 (n = 12)	Normal: 91.7%; High: 8.3%
Antiplatelet antibodies (n = 8)	Normal: 50%; High: 50%
Homocysteine (n = 8)	Normal: 50%; High: 50%

^a^ EXTEM: extrinsic pathway (tissue factor) clotting time, ^b^ EXTEM A10: amplitude of formed clot 10 min after formation, ^c^ FIBTEM A10: fibrin-dependent clot formation, amplitude of the formed clot at 10 min, ^d^ INTEM CT: intrinsic pathway clotting time.

 Anti-PF4 antibody measurement through immunoassays and functional assays were positive in 86.2%^2,11,22,23,25,26,30-33,41-46,50-54,56-58,61-63,65,67-71^ and 73% of cases, ^22,23,31,32,34,41-43,56,58,64,68,70^ respectively. Details on anti-PF4 antibody assays of case reports on VITT after ChAdOx1 nCov-19 vaccination is illustrated in Table S5.

**Figure 2:**
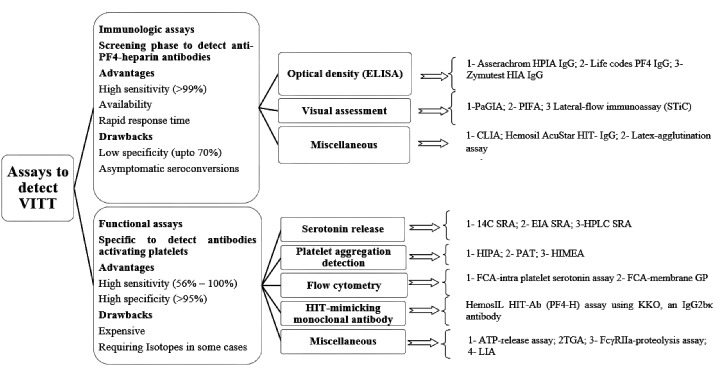
Functional and immunologic assays for detection of anti-PF4 antibodies. Abbreviations:ATP, adenosine triphosphate; 14C, carbon 14; EIA, enzyme immunoassay; FCA, flow-cytometry assay; GP, glycoprotein; HPLC, high-pressure liquid chromatography; HIT, heparin-induced thrombocytopenia; HIMEA, heparin-induced multielectrode aggregometry; HIPA, heparin-induced platelet activation; KKO, murine HIT–like monoclonal antibody; LIA, latex immunoturbidimetric assay; PF4, platelet factor 4; PAT, platelet-aggregation assay; SRA, serotonin-release assay; TGA, thrombin-generation assay; PaGIA, particle-gel immunoassay; PIFA, Particle-immunofiltration assay

 Binding of viral protein and free DNA particles in the vaccine to PF4 and generation of specific antigens are assumed to stimulate the secretion of antibodies against PF4 further. The immune complex of the PF4-IgG antibody and heparin activates platelets via interaction with cellular FcγRIIa receptors on the platelet surface. Subsequent clotting will be developed in combination with thrombocytopenia known as VITT.^39^ VITT resembles HIT in terms of clinical symptoms and pathobiology. Autoimmune or spontaneous HIT syndrome provides anti-PF4 antibodies despite the absence of previous exposure to heparin and includes persisting HIT, spontaneous HIT syndrome, flush heparin HIT, and fondaparinux-associated HIT.^62^ The serological and clinical features of autoimmune HIT are like those seen in VITT. In contrast to HIT, platelet activation in VITT occurs in the presence of PF4 rather than low heparin concentrations. The specific detection of antibodies contributed to ChAdOx1 nCov-19 vaccination should be regarded as a crucial task in confirming suspected VITT. The GTH expert committee highly recommends screening anti-PF4 antibodies if any thromboembolic events or thrombocytopenia observed during 2 weeks upon vaccination.^8^ There are two main approaches to verify the existence of VITT-related antibodies as is demonstrated in Figure 2. The preliminary screening phase is employment of immunoassays to confirm the presence and quantify the level of anti-PF4 antibodies. The further confirmatory step is via utilizing functional assays to detect the presence of platelet –activating antibodies independently of heparin.

 As it has been reported in the results, the immunoassays were used to detect anti-PF4 antibodies in more than 82% of all-evaluated cases and resulted in 85% positive outcomes. Generally considering, immunoassays are categorized in 3 main subgroups namely optical density-based assessments, visual-based assessments as well as miscellaneous (such as CLIA and Latex-agglutination assay). Although screening for anti PF4 antibodies via a particle gel immune assay (ID-PaGIA) was positive in a few cases,^57^ lack of precise sensitivity to recognize anti-PF4 antibodies were observed by PaGIA, and CLIA. While the results of a couple of studies exhibited the acceptable sensitivity of immunoassays such as Immucor^®^ (which employs polyvinyl sulfonate and PF4) to recognize antibody-inducing VITT with up to 100% accuracy, employment of PaGIA or CLIA was not sufficiently precise. A prominent conclusion is indicative of the fact that establishing a diagnosis of VITT may require a combination of diverse tests. CLIA was previously reported to be well sensitive for HIT antibodies while VITT antibodies were not desirably responsive to CLIA which is possibly due to differences between epitopes in VITT and HIT antibodies.^4^

 It is additionally worth noting that, among diverse IgG-specific ELISAs, the sensitivity and specificity has been reported to significantly vary in HIT from that of VITT. For instance, Asserachrom HPIA IgG has demonstrated 91.1 and 100% sensitivity and specificity for diagnosis of VITT while 72 and 93.8% sensitivity and specificity for diagnosis of HIT. Lifecodes PF4 IgG and Zymutest HIA IgG assays provide 94.1 and 77.8 % sensitivity and specificity to detect VITT while supplying more than 99 and 85% respective sensitivity and specificity to quantify HIT. It is worth considering that HemosIL AcuStar HIT-IgG and STic Expert, as frequently applicable rapid tests, with almost complete specificity for detection of both VITT and HIT are poorly sensitive to VITT-related antibodies, up to 5.9 and 4.2%, respectively. The sensitivity of both mentioned assays to detect HIT antibodies is more than 98%.^74^ In conclusion, the immunoassays, including HYPHEN BioMed ZYMUTEST and the Immucor, could be introduced as acceptably sensitive for VITT-relevant antibodies. The results of other comprehensive investigations concluded that for detection of VITT, the ELISA assays from Stago, Hyphen, and LIFECODES were acceptable. The assays from Acustar HIT assay, LIA, Stago STIC, were not reliably sensitive.^75^

 Immunoassays to detect antibodies against the PF4/heparin complex exhibit positive results should be further evaluated via functional tests since all existing tests for HIT diagnosis are not necessarily validated to detect antibodies involved in pathogenesis of VITT. Functional assays are technically complicated procedures to perform routinely. They are classified as serotonin release- based assay, platelet aggregation detection, flow cytometry, HIT-mimicking monoclonal antibody- based assay and miscellaneous (such as Latex immunoturbidimetric assay). In the current review, 63 cases were evaluated through functional assays which platelet-activating antibodies were detected in 73% of the totally assessed cases.

 It is recommended that upon positive immunoassays of PF4-heparin antibodies, a heparin-induced platelet activation (HIPA) assay or serotonin-release assay (SRA) be performed to confirm the diagnosis further. The advantage of HIPA over SRA is visual confirmation of platelet aggregation, unlike SRA, which makes use of radioactive agents.^76^ Simpler technologies are flow cytometry, turbidometry, aggregometry, and assessment of luciferase activity. For functional assays, measured end points, employed technology, accurate selection of platelet-donor, varied types of heparin and between laboratory differences should be regarded as critical variables which have influence on interpretation of achieved results.^77^

 Functional assays can detect platelet-activating antibodies in both typical and autoimmune HIT. If conventional HIPA or SRA tests do not represent autoimmune HIT, a modified HIPA test is suggested which indicates the vaccine-induced prothrombotic immune thrombocytopenia with a different reaction pattern.^8^ Similarly, it has been exhibited that in VITT, the SRA could investigate anti-PF4 IgG in the absence of heparin.^78^

**Figure 3:**
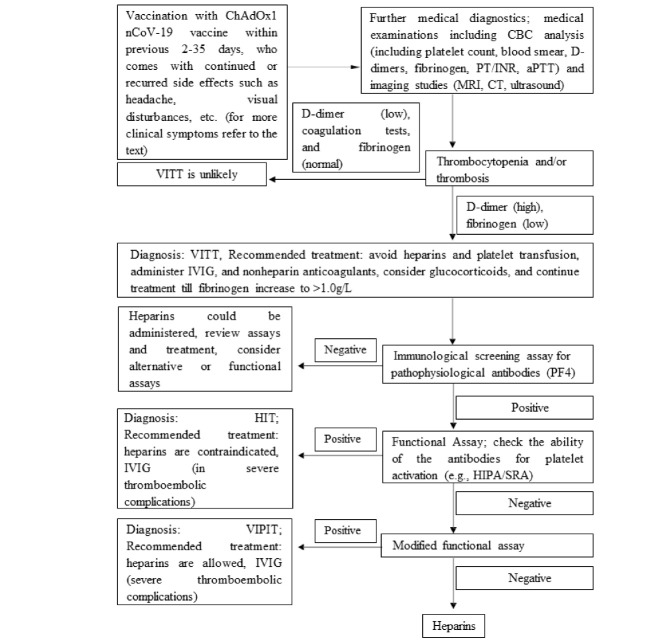
Recommended algorithms for diagnosis and treatments of patients with suspected ChAdOx1 nCoV-19 vaccine-induced TTS, 2 to 35 days after ChAdOx1 nCoV-19 vaccination

 Recommended algorithm for diagnosis and treatments of patients with suspected ChAdOx1 nCoV-19 vaccine-induced TTS, 2 to 35 days after ChAdOx1 nCoV-19 vaccination is outlined in Figure 3. Based on the GTH guideline, in the screening test for PF4/heparin antibodies with a positive result, functional confirmatory tests should be performed. These assays detect antibodies able to activate platelets dependent on (typical HIT) or independent of exogenous heparin (autoimmune HIT).^8^ About 43% of the cases in this systematic review had no previous exposure to heparin and therefore, positive test results in theses cases can establish the diagnosis of other mechanisms including autoimmune HIT.

 As the unknown mechanism and pathophysiological basis of VITTS after the administration of ChAdOx1 nCoV-19 vaccine, careful consideration should be prompted for the treatment. Platelet transfusion can provide further substrate for coagulopathy through antibody-mediated platelet activation mechanism.^22^ A comprehensive understanding of the exact mechanism involved in ChAdOx1 nCov-19 vaccine associated TTS may allow for more targeted and efficient therapeutic measures.

 There was no evidence yet suggesting the exacerbation of the patient condition after heparin administration, however, anticoagulation using a non-heparin anticoagulant including danaparoid, fondaparinux, or Argatroban, or DOACs is recommended. IVIG administration has been shown to be useful in patients with autoimmune HIT as the closest clinical manifestation to VITTS syndrome. Furthermore, plasma exchange with plasma instead of albumin can cause a temporary reduction in the levels of pathologic antibodies leading to amelioration of the coagulopathy in terms of hypofibrinogenemia.^22^

 Gundry and his colleagues used the PLUS cardiac test (GD Biosciences, Inc, Irvine, CA) as a clinically validated examination to measure different protein-based markers leading to the generation of a 5-year risk predicting the score of acute coronary syndrome. Their results showed that the COVID-19 mRNA vaccines could substantially raise endothelium inflammation and T cell infiltration in the cardiac muscle. This might justify the high rate of cardiomyopathy, thrombosis, and different vascular problems observed after mRNA-based vaccination. Accordingly, it is recommended to do the same study with ChAdOx1 nCov-19 induced TTS patients, which can help elucidate the possible TTS mechanisms in these patients.

 The most practical implication of these findings is that a low threshold should be considered to request immunoassays and confirmatory functional assays in cases with unexpected VITT clinical symptoms upon ChAdOx1 nCov-19 vaccination. No individual immunoassay test can detect all feasibly existing VITT cases, and if a single test becomes negative, a further ELISA or a functional assay should be ordered in case of strong clinical features of VITT. It is additionally recommended that upon positive ELISA results, functional assays perform as well.

## Conclusion

 ChAdOx1 nCov-19 vaccine-induced VITTS is an unusual and potentially fatal disorder that necessitates early diagnosis and appropriate therapeutic measures. Accordingly, familiarity with the clinical and laboratory features as well as diagnostic methods of this clotting complication is of great importance for health care providers to reduce the mortality rate in VITT patients. Further investigations on the pathophysiologic and molecular mechanisms of ChAdOx1 nCov-19 vaccine-induced VITTS may improve the diagnostic and therapeutic measures.

## Acknowledgments

 We appreciate the support from the Iran University of Medical Sciences.

## Competing Interests

 The authors declare no conflict of interest.

## Data Availability Statement

 All data generated or analyzed during this study are included in this published article [and/or its supplementary material].

## Ethical Approval

 Not applicable.

## Supplementary Files

Supplementary file 1 contains Table S1a, Table S1b and Tables S2-S5.
