# Supplementary material for: ChAdOx1 nCoV-19 Vaccine and Thrombosis with Thrombocytopenia Syndrome among Adults: A Systematic Review
Source: Adv Pharm Bull. 2023 Apr 29;13(4):723–35. doi: 10.34172/apb.2023.081 (PMC10676559; doi:10.34172/apb.2023.081)
Supplement: Supplementary file 1 — contains Table S1a, Table S1b and Tables S2-S5. [file apb-13-723-s001.pdf]

Adv Pharm Bull, 2023, 13(4), 723-S1.
doi: 10.34172/apb.2023.081
https://apb.tbzmed.ac.ir

Supplementary Tables

Table S1a. Details on patient characteristics, therapeutic measures, and outcome of case reports on VITT after ChAdOx1 nCov-19 vaccination

Author	Patient code	Gender	Age	Ethnicity	Place of measure	1st dose	Medical history (Pre-existing conditions/family history of diseases)	Previous exposure to heparin	Medication on admission (Regular medications)	Smoker	Chief complaint/ initial symptom	Days after vaccination to initiate first symptoms	Therapeutic measures	Medical examination/imaging on admission<br><br>Imaging method and site of thrombosis/hemorrhages	Outcome
Greinacher*	G9-1	F	49	NA	Germany	Yes	*	No	NA	NA	fatigue, myalgia, headache, chills, fever, nausea and epigastric discomfort	5	platelet concentrate-transfer to a tertiary hospital, IV antibiotics, analgesia, LMWH (enoxaparin, SC), Low-dose IV unfractionated heparin, red blood cell and platelet transfusions, prothrombin complex concentrates, recombinant factor VIIa	CT: PVT, peripheral PE, SVT, AT (infrarenal aorta), thrombi in both iliac arteries, CVT, splenic/upper mesenteric vein thrombosis, diffuse gastrointestinal bleeding (reduced perfusion of intestinal wall and pancreas)<br>involved by SVT, ascites	Died
	G9-2	F	35	NA	Germany	NA	*	No	NA	NA	one or more thrombotic problems	10	NA	PE	Alive
	G9-3	F	48	NA	Germany	NA	*	No	NA	NA		8	NA	CVT	Died
	G9-4	F	35	NA	Germany	NA	demyelinating disease, *	No	NA	NA		4	NA	CVT	Died
	G9-5	F	43	NA	Germany	NA	antiphospholipid antibodies ☝, *	No	NA	NA		13	NA	CVT	Alive
	G9-6	F	22	NA	Germany	NA	*	No	NA	NA		7	NA	CVT	Alive
	G9-7	F	36	NA	Germany	NA	*	No	NA	NA		8	NA	CVT	Alive
	G9-8	F	46	NA	Germany	NA	*	No	NA	NA		14	NA	CVT	Alive
	G9-9	M	24	NA	Germany	NA	*	No	NA	NA		16	NA	CVT	Died
Al-Mayhani	A1	F	35	Asian	UK	NA	NA	NA	NA	NA	Day 6: episodic right temporal and periorbital headache,<br>day 11: left face, arm	6	urgent decompressive hemicraniectomy, IVIG, plasmapheresis<br><br>intermediate dose fondaparinux	Non-contrast<br>CT and<br>CTA, CT head:<br>MCA with extensive Ischemia/ haemorrhagic transformation with mass effect,<br>right PVT,	Died

											and leg weakness, right gaze preference and drowsiness			herniation of the brain in decompressive hemicraniectomy	
	A2	F	37	White	UK	NA	NA	NA	NA	NA	Day 12: diffuse headache, left visual field loss, confusion, and left arm weakness.	12	IVIG, two IV pulses of methylprednisolone, plasmapheresis, fondaparinux	CTA/MRI: internal carotid arteries occlusion, left transverse sinus thrombosis, bilateral acute infarcts in a border zone distribution, PE, thromboses of the left transverse and sigmoid sinuses, left jugular, right hepatic and both iliac veins	Alive
	A3	M	43	Asian	UK	NA	NA	NA	NA	NA	Day 21: dysphasia	21	platelet transfusion, IVIG, fondaparinux	CT/MR: acute left frontal and insular infarct corresponding to the anterior cortical territory of the left MCA, with small haemorrhagic transformation. MR/CT venography: no evidence of CVST in primary examination	Alive
Scully<br><br>Additional thrombosis events associated with progression occurred in patients who received platelet transfusions or heparin-based therapy at presentation	Scu-1	F	30	NA	UK	Yes	All patients had no history of medical condition except for 1 case with a DVT history	NA	All patients had no history of use of a medication with a potential to precipitate thrombosis except for one case with OCP use	NA	Mild bruising and petechiae in some cases, secondary cerebral hemorrhage in some cases who had CVT, clinically significant bruising with no other hemorrhagic symptoms in 1 case with no thrombosis, 48-72h: headaches, fevers, and muscle aches in some cases	13	NA	CVT, PVT, PE, Ischemic bowel with infarction	Alive
	Scu-2	F	55	NA	UK	Yes				NA		6	NA	PVT, AAT, ICH	Died
	Scu-3	F	26	NA	UK	Yes				NA		12	NA	CVT	Alive
	Scu-4	F	52	NA	UK	Yes				NA		10	NA	Postmortem: thrombosis in the lungs and intestine, CVT, ICH	Died
	Scu-5	M	38	NA	UK	Yes				NA		14	NA	Extensive bilateral PE with heart strain	Died
	Scu-6	F	49	NA	UK	Yes				NA		15	NA	CVT, IJVT, SAH	Alive
	Scu-7	M	25	NA	UK	Yes				NA		9	NA	CVT	Died
	Scu-8	M	32	NA	UK	Yes				NA		19	NA	CVT	Alive
	Scu-9	F	35	NA	UK	Yes				NA		9	NA	CVT	Alive
	Scu-10	M	77	NA	UK	Yes				NA		8	NA	PE	Alive
	Scu-11	M	66	NA	UK	Yes				NA		12	NA	DVT, adrenal hemorrhage	Alive
	Scu-12	M	34	NA	UK	Yes				NA		14	NA	CVT	Alive
	Scu-13	M	54	NA	UK	Yes				NA		10	NA	PVT, MI	Died

	Scu-14	Female	71	NA	UK	Yes				NA		14	NA	Hemorrhagic symptoms only	Alive
	Scu-15	F	22	NA	UK	Yes				NA		10	NA	CVT, ICH	Alive
	Scu-16	F	39	NA	UK	Yes				NA		10	NA	MCA infarct	Alive
	Scu-17	F	70	NA	UK	Yes				NA		17	NA	PE (saddle embolism) with cardiac arrest, DVT in the leg	Alive
	Scu-18	M	21	NA	UK	Yes				NA		10	NA	MCA infarct	Alive
	Scu-19	F	46	NA	UK	Yes				NA		14	NA	CVT	Alive
	Scu-20	F	32	NA	UK	Yes				NA		12	NA	CVT	Died
	Scu-21	M	48	NA	UK	Yes				NA		14	NA	CVT	Alive
	Scu-22	F	49	NA	UK	Yes				NA		24	NA	PE	Alive
	Scu-23	F	46	NA	UK	Yes				NA		10	NA	CVT	Alive
Schultz	Sch-1	F	37	NA	Norway	Yes	Pollen allergy	No	Contraceptive pill	NA	Day 6: headaches, fever, transient numbness in the right foot and right sided visual disturbance, severe thrombocytopenia, Day 7: headaches Day 8: fever, persistent headaches	7	Dalteparin, platelet transfusions, decompressive craniectomy, initial low dose of LMWH, heparin, 7 platelet units, no other treatments	Cerebral CT/MRI: CSVT in the left transverse and sigmoid sinus, left occipital, temporo-occipital, subarachnoid/left cerebellar hemorrhage, with significant mass effect, cortical veins, left transverse sinus, sigmoid left sinus	Died
	Sch-2	F	42	NA	Norway	Yes	Pollen allergy	No	Contraceptive vaginal ring	NA	Day 7: headaches, Day 10: severe headaches, nausea, vomiting, fluctuating level of consciousness and right sided hemiparesis	7	Hemicraniectomy, Dalteparin, 19 platelet units, methylprednisolone, IVIG	Initial cerebral CT/venography: left sided lobar, heterogenous hemorrhage with fluid levels, contrast defects in the left transverse, sigmoid sinus. MRI venography: CSVT, CoVT. CT: brain herniation, cortical SAH, Cortical veins, left transverse sinus, and left sigmoid sinus	Died
	Sch-3	M	32	NA	Norway	Yes	Asthma	No	None	NA	Day 7: backache, abdominal pain, headaches	7	IVIG, prednisolone. Dalteparin, discharged with warfarin and tapering doses of prednisolone (Reduced dose of LMWH, 2	Normal abdominal ultrasound, thoracoabdominal CT, Contrast-enhanced MRI, abdominal CT portal vein: left hepatic vein,	Alive

													platelet units, prednisolone)	splenic vein, azygos vein, hemiazygos vein, and several basivertebral veins	
	Sch-4	F	39	NA	Norway	Yes	None	No	None	NA	Day 8: abdominal pain and headaches, mild thrombocytopenia, Day 10: increased headaches	8	Dalteparin, prednisolone, IVIG. Discharged with warfarin, prednisolone in tapering doses (Reduced dose of LMWH, no platelet units, prednisolone)	Examination: no neurological deficits, cerebral CT: small cerebellar hemorrhage, MR venography: CSVT in inferior sagittal sinus, vein of Galen/ straight, right transverse/sigmoid sinuses, CT pulmonary angiogram: bilateral segmental PE, abdominal CT: thrombosis in uterine veins	Alive
	Sch-5	F	54	NA	Norway	Yes	HTN	No	Hormone-replacement therapy, antihypertensive agents	NA	Day 6: unwell feeling, numbness of left-sided limbs Day 7: progression of the symptoms: headaches, nausea, left sided weakness (stroke symptoms)	6	Platelet transfusion before admission, methylprednisolone, IVIG, endovascular intervention with thrombectomy after unfractionated heparin, decompressive hemicraniectomy, withdrawn of treatment 2 days later (due to an uncontrollable increase in intracranial pressure), Heparin, 2 platelet units, methylprednisolone	Examination: somnolent (GCS: 14), several skin bruises, left sided hemiparalysis, facial nerve palsy, cerebral CT: right hemispheric parenchymal hemorrhage, repeat cerebral imaging: progression of venous infarction/parenchymal/subarachnoid hemorrhage, CSVT in all major venous sinuses, CT, CT scan with venography: cortical veins, superior sagittal sinus, both transverse sinuses, left sigmoid sinus	Died
Greinacher	G11-1	F	49	NA	Germany and Austria	Yes	previously healthy	No	NA	NA	few days after vaccination: fatigue, myalgia, headache Day 5: chills, fever, nausea, epigastric discomfort; Day 10: epigastric discomfort/nausea	5	Heparin, a platelet concentrate, IV antibiotics, analgesia, 4000- unit of LMWH (enoxaparin) SC, Low dose IV unfractionated heparin, Red cell/platelet transfusions, prothrombin complex concentrates, recombinant factor VIIa	BP: 125/88 mm Hg; HR 65 bpm; T 36.5°C, physical examination: unremarkable, moderate epigastric pain on palpation, CT: CVT, SVT, PVT, PE, AT	Died
	G11-2	F	35	NA	Germany and Austria	NA	No	No	NA	NA	One or more thrombotic complications	6	LMWH, DOACs	PE	Alive
	G11-3	F	48	NA	Germany and Austria	NA	No	No	NA	NA		9	Heparin treatment: Unknown	CVT	NA
	G11-4	F	35	NA	Germany and Austria	NA	CND	No	NA	NA		7	Heparin	CVT	Died

	G11-5	F	43	NA	Germany and Austria	NA	anticardiolipin antibodies, von Willebrand disease, FVL	No	NA	NA		13	Heparin	CVT, SVT, PE, right intraventricular, iliofemoral vein, IVC thrombosis	Alive
	G11-6	F	22	NA	Germany and Austria	NA	No	No	NA	NA		7	Heparin treatment: Unknown	CVT	Alive
	G11-7	F	36	NA	Germany and Austria	NA	No	No	NA	NA		8	Heparin	CVT	Alive
	G11-8	F	46	NA	Germany and Austria	NA	No	No	NA	NA		8	No Heparin treatment	CVT, post-mortem detected: Widespread Microvascular (brain, lungs, kidneys)	Died
	G11-9	F	24	NA	Germany and Austria	NA	No	No	NA	NA		16	No Heparin treatment	CVT, post-mortem detected: Multiple organ thrombi	Died
	G11-10	M	NA	NA	Germany and Austria	NA	No	No	NA	NA		11	No Heparin treatment	CVT, SVT	Died
	G11-11	M	NA	NA	Germany and Austria	NA	Unknown	No	NA	NA		12	No Heparin	post-mortem detected: Cerebral hemorrhage	Died
Bayas	Ba-1	F	55	NA	Germany	Yes	No	NA	NA	No	Day 0 and day 7: marked flu-like symptoms and fever, Day 10: conjunctival congestion, retro-orbital pain, diplopia	0	Dexamethasone (IV), therapeutic heparinization, levetiracetam, lacosamide, anticoagulation switched to phenprocoumon	Examination: binocular diplopia at vertical/right lateral gaze, visual acuity: 0·85 (both eyes), MRI: SOVT, no contrast filling/bilateral high T2 signal intensity of superior ophthalmic vein, ischaemic stroke in left parietal lobe, MCA territory/restricted diffusion	Alive
Blauenfeldt	Bl-1	F	60	Danish	Denmark	Yes	Hashimoto thyroiditis, HTN	NA	losartan, simvastatin, levothyroxine	NA	Day 1: headaches, Day 7: strong, persistent abdominal pain, Day 8: left-sided weakness and eye deviation to the right	1	Hydrocortisone (substitution therapy), cefuroxime, seven pools of platelet concentrate, hemicraniectomy, postoperative Dalteparin	UA: positive for blood CT abdomen: bilateral adrenal hemorrhages, subcapsular renal hematoma, MRI: diffusion restriction/ completed infarction in entire area, right MCA, midline shift, edema of right hemisphere, no new ischemic/hemorrhagic lesions, CT of aorta: excluded dissection, malignant media infarction	Died
Mehta	Meh-1	M	32	White	UK	Yes	No	NA	None	Ex	thunderclap headache, left-sided incoordination and hemiparesis	9	No specific haematological or immunological treatments (neurological condition deteriorated rapidly), repeated doses of mannitol and hypertonic	Rapid GCS fall, seizures, significant cortical oedema, haemorrhagic progression, signs of significant cerebral oedema, midline shift, cerebellar herniation, volumetric coronal non-	Died

													saline, intubation, ventilation	contrast CT, volumetric axial CT venogram, volumetric axial and sagittal non-contrast CT: superior sagittal sinus thrombosis, cortical veins thrombosis: diffuse; prominently involving right superficial anastomotic vein, Clot burden (Heavy): significant venous expansion, SAH: Mainly cortical, Intraparenchymal haemorrhage: Extensive (venous distribution)	
	Meh-2	M	25	White	UK	Yes	primary sclerosing cholangitis (PSC) and migraines, no family history	NA	Ursodeoxycholic acid, Budesonide, Sumatriptan, Amitriptyline	Yes	worsening headache, photophobia, neck stiffness, visual disturbances, non-blanching petechial rash on lower limbs, bleeding gums, left hemiparesis and hemisensory loss, focal motor seizures	6	Unfractionated heparin, dexamethasone, IVIG, platelet transfusions	volumetric coronal and axial non-contrast CT, volumetric sagittal and axial CT venogram: Superior sagittal sinus, Cortical veins involved: diffuse, Clot burden: Heavy; significant venous expansion, SAH: cortical and basal cisterns, intraparenchymal haemorrhage: extensive (venous distribution)	Died
Castelli	Cas-1	M	50	Caucasian	Italy	Yes	volunteer blood donor (normal previous routine blood tests), heterozygous MTHFR mutation	NA	NA	NA	Day 7: severe headache, Day 11: slight deviation of the right buccal rim, loss of strength in the right lower limb, unstable walking, slight visual impairment	7	fibrinogen concentrate, platelet (4 units), bilateral decompressive craniectomy	Examination: apyretic, BP 150/80 mmHg, HR 80/min, SpO2 99%, GCS 15, pain 8/10, GCS fall, right hemiplegia, localization of painful stimulus to left, no execution of orders nor verbal production, isochoric, isocyclic pupils/vomiting,brain CT: intra-parenchymal haemorrhage in left hemisphere, CT angiography: multiple bleeding spots in parenchymal haemorrhage, lack of opacification of left transverse/sigmoid sinuses, thrombosis of venous sinuses, increase in haemorrhagic focus, initial transtentorial herniation of left temporal uncus/shift of	Died

														midline to right, diffuse thrombosis of cortical veins	
D'Agostino	D-1	F	54	NA	Italy	NA	Meniere's disease	NA	None	NA	Acute cerebrovascular accident	12	transfer to hemodynamics room, POBA of right coronary artery, without stent implantation, with restoration of distal flow, with persistence of extensive thrombosis of vessel with a theoretic indication for administration of intracoronary antiplatelet agents (Aggrastat), given hematological/neurological status, such therapy was not performed, progressive worsening of neurological state, until a GCS 6 during the procedure, transfer to ICU	Examination: GCS 13, ecocolordoppler, left side signs, normothermic/ normoconformed lower limbs, preserved femoral/popliteal/posterior tibial wrist bilaterally, ECG: MI, ecocolordoppler: DVT, tests excluded sepsis, various infections, malignancy, vascular diseases, toxic/immunological reactions, no severe trauma, brain CT, brain angio-CT, thorax angio-CT, Brain MRI, abdomen angio-CT, brain CT: DIC, multiple subacute intra-axial hemorrhages in atypical locations: right frontal/temporal lobes, ipsilateral hemorrhagic subarachnoid suffusion, suspicion of Labbè/superior longitudinal sinus thrombosis, a non-occlusive thrombosis of Galen vein, floating thrombus in aortic arch, multiple contrast filling defect with multi-vessel involvement: left upper lobe segmental branches, left interlobar artery, right middle lobe segmental branches, right interlobar artery, acute basilar thrombosis, superior sagittal sinus thrombosis, delineation of hyperacute ischemic lesions in vascular territory of right posterior cerebral artery, perforating pontine branches, filling defects at left portal branch, level of right suprahepatic vein, adrenal hemorrhage, blood in pelvis, diffuse ischemic hypodensity: right occipito-temporal, superior cerebellar regions, right thalamic, internal capsula regions, pons, mesencephalon, conditioning edema-based	Died

														mass effect, contralateral shift of midline structures	
Esba	E-1	F	61	NA	Saudi Arabia	NA	DM, HTN, DLP, high liver enzymes	NA	NA (refer to comorbidities)	No	Day 14: left ear pain, headache, dizziness, nausea, tinnitus, few days later: disorientation, confused, complained of eye pain, impaired vision	14	Enoxaparin	brain CT, pan CT: CVST, complete thrombosis of the left transverse sinus extending to the left sigmoid sinus/jugular vein, pan CT: no malignancies	Alive
	E-2	M	40	NA	Saudi Arabia	Yes	No	NA	No	Yes	Day 14: severe headache, nausea, Day 16: worsening headache	14	Enoxaparin	brain CT: CVST	Alive
Franchini	F-1	M	50	White	Italy	Yes	voluntary periodic blood donor (normal lab blood tests) no medical/family history, never suffered from COVID-19, heterozygous of MTHFR mutation	NA	NA	NA	Day 7: worsening headache, Day 11: unconsciousness	7	urgent neurosurgery, RBC (9U), platelet apheresis (4U), fibrinogen concentrate infusion	Brain CT, CT angiography of intracranial circle vessels: intra-parenchymal hemorrhage in left cerebral hemisphere, multiple small bleeding spots in the context of the left parenchymal hemorrhage, lack of opacification of the left transverse/sigmoid sinuses (CVST)	Died
Garnier	Gar-1	F	26	NA	France	NA	NA	NA	NA	NA	Day 0: nausea, headache, Day 8: acute stroke, right hemiplegia, aphasia, persistent nausea, headache	0	Dual thrombo-aspiration using ADAPT technique, i.e., without stent retriever, led to recanalization of left MCA after a first pass, corticosteroids, plasmatic exchange, anticoagulants	Examination: right hemiplegia and aphasia Initial angiography, final angiography, CT, MRI: proximal left MCA occlusion, thrombolysis in cerebral infarction scale, DIC, segmental PE, portal thrombosis extending to the splenomesenteric trunk/ileal veins, (eupneic/no abdominal pain), global arterialization of liver parenchyma with central hypoattenuating areas, that became isoattenuating on portal venous phase, venous mesenteric ischemia, minor sylvian/border zone infarcts, mild hemorrhagic changes, gripping difficulties, minor phasic	Alive

														troubles, recanalization based on thrombolysis in cerebral infarction scale, transient hepatic attenuation differences due to hepatic blood flow alterations	
Geeraerts	Ge-1	NA	NA	NA	France	NA	NA	NA	NA	NA	NA	NA	Heparin, corticosteroids, IVIG in one patient, decompressive craniectomy	severe CVT	Died
	Ge-2	NA	NA	NA	France	NA	NA	NA	NA	NA	NA	NA		severe CVT	Died
Gras Champel	GC-1	M	41	NA	France	NA	No	NA	well tolerated oral estroprogestative contraception in 4 patients	NA	NA	17	NA	Radiologic: CVT, splanchnic thrombosis, other thrombosis, DIC	Died
	GC-2	M	63	NA	France	NA	No	NA		NA		11	NA	Radiologic: CVT, Splanchnic thrombosis, other thrombosis, DIC	Died
	GC-3	F	21	NA	France	NA	No	NA		NA		15	NA	Radiologic: CVT, Splanchnic thrombosis, other thrombosis	Alive
	GC-4	F	69	NA	France	NA	No	NA		NA		12	NA	Radiologic: CVT, Other Thrombosis, DIC	Died
	GC-5	F	26	NA	France	NA	No	NA		NA		9	NA	Radiologic: Splanchnic thrombosis, other thrombosis, DIC	Alive
	GC-6	M	73	NA	France	NA	No	NA		NA		12	NA	Radiologic: Splanchnic thrombosis, other thrombosis	Alive
	GC-7	F	61	NA	France	NA	No	NA		NA		13	NA	Radiologic: Splanchnic thrombosis, other thrombosis, DIC	Died
	GC-8	F	38	NA	France	NA	No	NA		NA		8	NA	Radiologic: CVT, Splanchnic thrombosis, DIC	Died
	GC-9	F	74	NA	France	NA	No	NA		NA		15	NA	Radiologic: Other thrombosis, DIC	Alive
	GC-10	M	23	NA	France	NA	No	NA		NA		9	NA	Radiologic: CVT, DIC	Alive
	GC-11	F	44	NA	France	NA	No	NA		NA		9	NA	Radiologic: CVT, DIC	Alive
	GC-12	M	60	NA	France	NA	No	NA		NA		11	NA	Radiologic: CVT	Alive
	GC-13	M	60	NA	France	NA	No	NA		NA		11	NA	Radiologic: Splanchnic thrombosis	Died
	GC-14	M	67	NA	France	NA	No	NA		NA		8	NA	Radiologic: Splanchnic thrombosis, DIC	Died
	GC-15	F	67	NA	France	NA	No	NA		NA		11	NA	Radiologic: DIC	Alive
	GC-16	F	73	NA	France	NA	No	NA		NA		10	NA	Radiologic: DIC	Alive
	GC-17	F	24	NA	France	NA	No	NA		NA		35	NA	Radiologic: CVT, Other thrombosis	Alive
	GC-18	M	51	NA	France	NA	No	NA		NA		6	NA	Radiologic: CVT	Alive
	GC-19	F	53	NA	France	NA	No	NA		NA		18	NA	Radiologic: CVT	Alive

	GC-20	M	54	NA	France	NA	No	NA		NA		25	NA	Radiologic: Splanchnic thrombosis	Alive
	GC-21	M	56	NA	France	NA	No	NA		NA		2	NA	Radiologic: Splanchnic thrombosis	Alive
	GC-22	F	61	NA	France	NA	No	NA		NA		17	NA	Radiologic: Splanchnic thrombosis	Alive
	GC-23	M	68	NA	France	NA	No	NA		NA		11	NA	Radiologic: Splanchnic thrombosis	Alive
	GC-24	M	73	NA	France	NA	No	NA		NA		30	NA	Radiologic: Splanchnic thrombosis	Alive
	GC-25	M	24	NA	France	NA	No	NA		NA		7	NA	Radiologic: Splanchnic thrombosis	Died
	GC-26	M	61	NA	France	NA	No	NA		NA		2	NA	Radiologic: Splanchnic thrombosis	Alive
	GC-27	F	58	NA	France	NA	No	NA		NA		8	NA	Radiologic: Splanchnic thrombosis	Alive
Jones	J-1	M	63	NA	UK	Yes	Obesity (BMI: 41.8 kg/m2), HTN	No	Anti-HTN	No	Day 20: cool, pale. painful left leg, decreased motor function, new onset of paresthesia of the left leg and foot, severe shortness of breath	20	therapeutic anticoagulation with IV UFH, emergency surgical thrombectomy of popliteal artery/all tibial arteries, tPA into tibial arteries intraoperatively, several transfusions of platelets/RBC, IV heparin, platelet transfusion, replaced unfractionated heparin with fondaparinux SC, IVIG (2 consecutive days, Gammunex), aspiration thrombectomy	Examination: hypoxic (O2Sat 85%), decreased motor, function Doppler, CT angiogram, echocardiogram, arteriograms: absent arterial Doppler signals (Rutherford IIB acute limb ischemia), left popliteal artery occlusion with no visible tibial vessels, bilateral segmental pulmonary artery thrombi, thrombus adherent to the wall of infrarenal aorta, large amount of thrombus in popliteal artery/all tibial arteries, partial rethrombosis of tibial circulation	Alive
Ramdeny	Ram-1	M	54	NA	UK	NA	rare congenital limb malformation with strong family history of a rare congenital limb deformity (mother, maternal grand father)	NA	NA	NA	Day 14: worsening headache, bruising, unilateral right calf swelling, thrombophlebitis of the right leg	14	IVIG, anticoagulation, danaparoid, DOAC	CT, ultrasonography: extensive CVST, PVT, thrombophlebitis of right leg	Alive
Wolf	W-1	F	22	NA	Germany	Yes	BMI: 26 kg/m2	NA	None	No	Day 0: shivering, fever, headaches for 2 days<br>Day 4: new frontally accentuated headaches,	0	Endovascular rheolysis, levetiracetam, enoxaparin, dabigatran	MRI, Digital subtraction angiography (DSA): blood in subarachnoid space adjacent to falx cerebri on both sides, superior sagittal sinus, left-hand transverse sinus, thrombosed sigmoid sinus, occlusion of	Alive

											Day 7: self-limited generalized epileptic seizure			ascending cerebral veins, and mentioned sinuses	
	W-2	F	46	NA	Germany	Yes	No	NA	None	No	Day 8: severe headaches, Day 13: focal neurologic symptoms with mild aphasia, hemianopia to the right, somnolence	8	Endovascular rheolysis, enoxaparin, danaparoid, dabigatran	MRI, DSA: thrombotic occlusion of superior sagittal sinus/left-hand transverse sinus/sigmoid sinus, acute intracerebral hematoma	Alive
	W-3	F	36	NA	Germany	Yes	No	NA	None	No	Day 7: severe headaches, fever, headache, Day 7-17: headaches, Day 17: acute somnolence, right-hand hemiparesis	7	Danaparoid, endovascular rheolysis, enoxaparin, dabigatran	MRI, DSA: thrombotic occlusion of straight sinus/a non-occlusive thrombus in superior sagittal sinus, congestive edema of both thalami (more in left side)	Alive
Xie	X-1	F	23	NA	UK	NA	No	NA	NA	NA	Presented with chest pain, breathlessness	<7	Apixaban. Intubation/ventilation, plasma exchange, IV methylprednisolone, heparin infusion	CT, Echo cardiogram, brain MRI: PE, RV thrombus, splenic vein thrombus, bilateral adrenal haemorrhage	Alive
Muster	Mu-1	F	51	NA	Austria	NA	Obesity (BMI: 31 kg/m2)	NA	NA	NA	dyspnoea, fatigue, cough	8	LMWH, dexamethasone, glucocorticoid therapy, dabigatran	Examination: afebrile, O2sat 98%, BP 150/90 mm Hg, HR 98 bpm, CT pulmonary angiography, MR venography, CT venography: central PE without right ventricular dysfunction, venous thrombus in left internal iliac vein, common iliac vein, IVC	Alive
Aladdin	Ala-1	F	36	NA	Saudi Arabia	Yes	DM	NA	DM medications	NA	left-sided convulsions (5min), weakness in the left arm, fever, vomiting, severe headache	14	Enoxaparin, antibiotics, antivirals, transfer to ICU, intubated, mechanically ventilated, ionotropic support, massive blood transfusion, hemodialysis	Examination: conscious, oriented, HR 117 bpm, GCS 15/15, unremarkable cranial nerves, left upper limb weakness, normal power of right upper/bilateral lower Limbs, deep tendon reflexes: brisk on left (Babinski sign), normal sensory/coordination examinations, brain CT, CT-venogram, CT abdomen/pelvis: superior sagittal thrombosis/thickened	Died

														cortical veins/bilateral hypodensites in parietal lobes, florid DIC, acute kidney injury, multiple new bifrontal/biparietal hypodensites, extensive dural venous sinus thrombosis of superior sagittal sinus/its cortical tributaries/proximal left transverse sinus, extensive PVT/superior mesenteric vein thrombosis/potential splenic/hepatic infarction	
Althaus	Alt-1	F	47	NA	Germany	Yes	No	No	NA	NA	neurological or hematological symptoms	7	endovascular rheolysis (1 case). All surviving patients: anticoagulation, Four patients: IVIG, non-heparin anticoagulation	non-enhanced CT, digital subtraction angiography, angiography after mechanical recanalization: CVST	Died
	Alt-2	F	48	NA	Germany	Yes	NA	No	NA	NA		6		Clinical/labfindings, CT, ultrasound imaging, autopsy: CVST, PE	Died
	Alt-3	M	24	NA	Germany	Yes	FVL mutation	No	NA	NA		10		Clinical/labfindings, CT, ultrasound imaging, autopsy: bleeding, multiple thrombosis	Died
	Alt-4	M	53	NA	Germany	Yes	No	No	NA	NA		9		Clinical/labfindings, CT, ultrasound imaging: DVT, PE	Alive
	Alt-5	F	47	NA	Germany	Yes	No	No	NA	NA		7		Clinical/labfindings, CT, ultrasound imaging: CVST	Alive
	Alt-6	M	32	NA	Germany	Yes	No	No	NA	NA		20		Clinical/labfindings, CT, ultrasound imaging: PE	Alive
	Alt-7 (previosly reported in wolf study-patient code: W-3)	F	36	NA	Germany	Yes	No	No	NA	NA		17		Clinical/labfindings, CT, ultrasound imaging: CVST	Alive
	Alt-8	F	29	NA	Germany	Yes	No	No	contra-ception	NA		7		Clinical/labfindings, CT, ultrasound imaging, repeated cerebral imaging: CVST	Alive
Bano	Ban-1	F	61	NA	UK	Yes	Asthma, HTN, obesity	No	hormone replacement therapy for 20yrs, indapamide	NA	Progressive dyspnoea, pain and swelling in the right leg	13	platelets, LMWH, anticoagulation switched to fondaparinux, further platelet transfusion withheld, IVIG, dexamethasone, switching anticoagulation to rivaroxaban	Examination: two-level Wells Score, CT pulmonary angiogram: bilateral PE (right heart strain), right main pulmonary artery extending into upper, middle/lower pulmonary arteries/left segmental pulmonary arteries	Alive

	Ban-2	F	53	NA	UK	Yes	Fibromyalgia	No	None	NA	headache and facial weakness, weakness of the right arm and leg (hemiparesis)	11	intubation, platelets, urgent neurosurgical intervention, multiple platelet transfusion	Head CT: extensive intracerebral haemorrhage, CT venogram: CVST, repeat scans: further bleeding, signs of ICP, superior sagittal sinus, right sigmoid, right transverse sinus	Died
	Ban-3	M	55	NA	UK	Yes	No	No	None	NA	Headache, dysphasia, right arm weakness, discoordination	8	Alteplase, multiple Platelet transfusion, Dexamethasone, argatroban, Cryoprecipitate, emergency decompressive craniectomy and ICP bolt insertion under the cover of platelet transfusion	Head CT, Repeat brain imaging: CVST, cortical veins, superior sagittal sinus, left transverse, left sigmoid sinus, left internal jugular vein, new SAH (midline shift/compression of midbrain)	Died
Bonato	Bon-1	F	26	Italian	Italy	Yes	No	No	combined (estrogen-progestogen) contraceptives	NA	Headache, right-sided weakness, visual disturbances	14	Fondaparinux, transfer to ICU, blood collection at different time-points for lab work-up, IVIG, dexamethasone, anticoagulation (argatroban), fondaparinux replacement with oral vitamin K antagonist	Examination: severe right-sided weakness, no visual field defects, CT: hyperdense rectus sinus and vein of Galen, MRI venography: multifocal venous thrombosis, bilateral occlusion of parietal cortical veins, straight sinus, vein of Galen, internal cerebral veins, inferior sagittal sinus, transverse sinuses, right parietal/left frontoparietal lobes with an extensive venous infarction/hemorrhagic transformation, follow-up CT scan: rectus sinus/vein of Galen: normal density with oedema in brain tissue on both hemispheres, Follow-up MRI venography: restored venous flow in rectus sinus/vein of Galen; right internal cerebral vein, bilateral frontoparietal cortical veins occluded, large intraparenchimal venous infarction	Alive
Bourguignon	Bour-1	F	72	NA	Canada	NA	No	NA	NA	NA	left limb pain and claudication	7	UFH, surgical embolectomy, argatroban, IVIG, oral apixaban	suprarenal aortic thrombus, occlusion of left superficial and deep femoral arteries, thromboses of celiac/right peroneal arteries	Alive
	Bour-2	M	63	NA	Canada	NA	No	NA	NA	NA	Day 18: cramping left leg, Day 22:	18	Tinzaparin, surgical embolectomy, switch from heparin to	CT angiography: acute arterial thrombosis in	Alive

											acute dyspnea, Day 23: cold and painful left leg cold		Fondaparinux, IVIG	left leg, extensive PE, lower-limb ultrasonography: nonocclusive right popliteal DVT, residual distal lower-limb thrombosis, distal foot ischemic necrosis (awaiting amputation)	
	Bour-3	M	69	NA	Canada	NA	non–insulin-dependent DM, HTN, OSA, prostate cancer, no thrombosis history	Yes	Aspirin	NA	Headache, confusion, progressive left-sided weakness	12	fondaparinux, IVIG, switch to rivaroxaban, therapeutic plasma exchange	right MCA stroke, hemorrhagic transformation, additional thromboses: right internal carotid artery, right cerebral transverse/sigmoid sinuses, right internal jugular vein, hepatic vein, distal lower-limb vein; PE, no new clinically evident thromboses, hemiplegia	Alive
Choi	Cho-1	M	33	Korean	Korea	Yes	No	NA	NA	NA	Day 0: fever, headache, Day 9: Headache, vomiting, Day 12: sudden onset of a tingling sensation in the right arm and mental change, drowsiness, neurological symptoms, dysarthria, right hemiparesis	0	Transfer to neurosurgical department/ICU, FFP, Platelet Concentrate, IVIG, methylprednisolone, Endovascular mechanical thrombectomy, plasma exchange	Examination: afebrile, slight HTN (155/90 mmHg), non-enhanced brain CT: subcortical hematoma in left parietal lobe/adjacent SAH, hemorrhagic venous infarction, MR venogram: left sigmoid sinus/ internal jugular vein visualized, remaining dural venous sinus not visualized, extensive CVT, venography: transverse sinus/superior sagittal sinus, extensive CVT	Died
Cliff-Patel	CP-1	M	33	NA	UK	Yes	NA	No	NA	NA	back pain, haematuria, headache, right lower leg pain	21	treated for pyelonephritis, IV, Argatroban, IVIG (stopped when platelet counts normalized-in 2–5 days), Warfarin, Argatroban (stopped upon establishment on warfarin)	Examination: bilateral flank/right lumbar tenderness, no clinical signs of DVT, low-grade fever, O2sat 96%, non-contrast CT of urinary tract: inflammatory changes in right kidney (recently passed kidney stone/pyelonephritis), ultrasound of right lower leg: negative for DVT, intracranial CT venogram: negative for VST, no symptoms of PE, CT pulmonary angiogram (CTPA): PE at bifurcation of left pulmonary artery, repeat CT scan of urinary tract with contrast: right	Alive

														renal vein thrombus extending into IVC, renal infarction	
	CP-2	M	28	NA	UK	Yes	NA	No	NA	NA	sudden-onset back pain and lower limb weakness	8		bilateral PEs, left proximal DVT, normal O2Sat, not showing any typical PE symptoms	Alive
	CP-3	M	61	NA	UK	Yes	NA	No	NA	NA	exertional dyspnoea and pleuritic chest pain	10		CTPA: extensive bilateral PEs	Alive
Gangi	Gan-1	M	47	NA	UK	Yes	HTN, Diverticulitis, no prior history of thrombosis	No	NA (refer to pre-existing conditions)	NA	Chest pain	7	Fondaparinux, Argatroban (during TPE), DOAC, IVIG, Steroids, TPE, clopidogrel, percutaneous coronary intervention	posterior-inferior ST-elevation MI, diagnostic angiogram: thrombosis in proximal circumflex/posterior descending arteries, no significant atheroma, PE, coronary artery and left atrial appendage thrombosis, unknown to have porto-mesenteric thrombosis	Alive
	Gan-2	M	28	NA	UK	Yes	Cardiomyopathy Secondary polycythemia, no prior history of thrombosis	No	NA (refer to pre-existing conditions)	NA	Severe headache, blurred vision, vomiting	3-4	Fondaparinux, IVIG	Non-contrast head CT: hyperdensity involving superior sagittal sinus/bilateral transverse sinuses, CT venography confirmed thrombotic disease within this distribution, CVT, unknown to have Porto-mesenteric Thrombosis and PE	Alive
	Gan-3	F	21	NA	UK	Yes	Left hip arthroscopy, no prior history of thrombosis	No	NA	NA	Pleuritic chest pain, hemoptysis shortness of breath	26	Fondaparinux, DOAC, IVIG, steroids	CTPA: PE, right heart strain and pulmonary infarcts, abdominal US/CT venography: not demonstrate portal vein, hepatic vein, or cerebral venous thrombosis, echocardiogram: right ventricular impairment/tricuspid regurgitation	Alive
	Gan-4	M	48	NA	UK	Yes	Depression, no prior history of thrombosis	No	NA (refer to pre-existing conditions)	NA	Headache retro-orbital pain, pleuritic chest pain, abdominal pain	3	Fondaparinux, Argatroban (during TPE), Warfarin, IVIG, Steroids, TPE	CT venography: thrombosis in right transverse sinus/right jugular vein, CT abdomen/pelvis: extensive occlusive thrombi in main portal vein, right/left portal vein branches, superior mesenteric vein, splenic vein, CT: acute thrombus in right renal infarct/right internal iliac artery, CVT, Porto-mesenteric	Alive

														Thrombosis, unknown to have PE, right internal iliac artery thrombus	
	Gan-5	M	54	NA	UK	Yes	Guillain Barre (April 2019), Covid-19 infection (2020), no prior history of thrombosis	Yes	NA (refer to pre-existing conditions)	NA	Headache	11	Fondaparinux, Warfarin, IVIG, Steroids, TPE	Seizures, CT: asymmetrical hyperdensity within left transverse, sigmoid and straight sinuses, CVT, CT venography: extensive filling defects in left transverse, sigmoid, straight sinuses, left jugular vein, CT of chest/abdomen/pelvis: no further thrombosis, CT (after seizure): left temporal cortical venous hemorrhage	Alive
	Gan-6	F	26	NA	UK	Yes	Asthma Depression PCOS Current smoker, no prior history of thrombosis	No	OCP (refer to pre-existing conditions)	Yes	Headache, photophobia, nausea	11	Transfer to tertiary neurological center, LMWH, fondaparinux, Warfarin, IVIG, Steroids	Head CT: hyperdensity of inferior sagittal/transverse sinuses, CT venography: thrombus in straight sinus, bilateral transverse sinuses/right internal jugular vein, abdominal US (due to raised alanine transaminase): intrahepatic main/right portal vein thrombosis, suspected cavernous transformation. CVT, Porto-mesenteric Thrombosis	Alive
Gattringer	Gatt-1	F	39	NA	Austria	Yes	No	NA	NA	NA	severe holocephalic headache	6	Danaparoid, dexamethasone, IVIG, switch to Argatroban	Examination: normal., Brain CT/venography/CT pulmonary angiography/ compression ultrasound (legs): unremarkable, Brain MRI: left sigmoid/transverse sinus thrombosis without brain parenchymal involvement	Alive
	Gatt-2	F	24	NA	Austria	Yes	No	NA	NA	NA	severe persisting headache	8	IVIG, dexamethasone, argatroban, dabigatran	Examination: normal, Brain MRI: thrombosis of two cortical veins/related small frontal right juxtacortical haemorrhage	Alive
Gessler	Gess-1	F	47	NA	Germany	Yes	No	NA	None	NA	Progressive headaches	7	IVIg, corticosteroids, platelets in all cases perioperatively, Intraoperatively, severe bleeding/venous stasis (challenge to surgeon) immaculate hemostasis, effort to avoid injury to brain, In all three cases: use of artificial hemostyptics/further transfusions controlled	rapid neurological deterioration, head CT/venograms: large-scale sinus thrombosis/ICH signs of herniation, no other venous thrombosis	Died
	Gess-2	F	44	NA	Germany	Yes	No	NA	None	NA	Progressive headaches	12			Died

													intraoperative bleeding, surgical decompression of affected hemisphere via DC, All patients: transfer to Neuro-intensive care unit postoperatively, Argatroban.<br>1 case: mechanical venous thrombectomy (postoperative increase in CVT), Postoperative/postinterventional imaging: no demonstrated improvement of CVT, progression of ICH/brain damage		
Graf	Gra-1	M	29	NA	Germany	Yes	NA	No	NA	NA	Day 9: headache, abdominal pain,<br>Day 12: emesis, abdominal cramps,<br>Day 14: severe headache, hematemesis	9	Transfer to department of neurology (no neurological deficits at that time point), IVIG, Argatroban antiepileptic drugs, transverse sinus: recanalized, as did portal/mesenteric veins	Gastroscopy: diffuse mucosal bleeding, no neurological deficits, MRI: complete thrombosis of left transverse/sigmoid sinus/left proximal jugular vein, no involvement of parenchyma/no bleeding due to congestion of sinus veins, abdominal CT angiography: extensive thrombosis of mesenteric/portal vein, profuse bleeding of stomach, CCT: new left temporo-parietal intracranial hemorrhage	Alive
Guan	Gua-1	M	59	East Asian	Taiwan	NA	No	NA	NA	NA	Headache, nausea, thunderclap headache, pain on the left side of neck	5	Apixaban, IVIG	T: 35.4 °C, BP: 129/90 mmHg, HR: 67bpm, RR: 180/min, examination: no neurological deficit, normal extra-ocular movement/visual power/without photophobia/phonophobia/ meningeal signs, non-enhanced CT: hyperdensity of sinus, Cord sign, dense vein sign at left transverse/sigmoid sinuses, CT venogram: CVST at left transverse sinus/sigmoid sinuses, thrombosis of left internal jugular vein	Alive
Huang	H-1	M	34	Asian	Taiwan	Yes	without COVID-19 history	No	None	NA	Day 0: fever,<br>Day 5: abdominal pain,<br>Day 7:	0	Dabigatran, IVIG	Examination: 88 kg, no signs of neurological deficits, upper respiratory infection, or photophobia, brain CT: lacunar infarct in right semicentral ovale	Alive

											persistent and severe headache			without hemorrhagic transformation, CT of abdomen: no intra-abdominal vessel thrombosis/hemorrhage	
Ikenberg	Ik-1	F	early 30s	NA	Germany	Yes	No	No	None	NA	Day 0: myalgia, holocephalic headache, chills, day 7: isolated headache, Day 10: persisting headaches	0	Argatroban, IVIG	Examination: no relevant findings in hypercoagulable state work-up, Day 7: neurological examination: normal, no meningism/petechia, cerebral MRI: without pathological finding/CVST, Day 10: Examination: discrete gait ataxia, progressive amnestic difficulties, discrete amnesic aphasia, MRI: CVST of left transverse/sigmoidal sinus, left-temporal/left-cerebellar intracerebral haemorrhage, MRI: CVST, regressive cerebellar haemorrhage, slightly progressive temporal perifocal oedema, small irregularities at bottom of left transverse sinus in contrast-enhanced T1w image, a high sensitivity for (small) abnormalities in cerebral veins/sinuses	Alive
Jacob	Ja-1	F	39	NA	UK	Yes	Migraines, BRCA2 carrier	No	None	No	Day 7 and 8: feeling unwell, unable to complete usual 25min walk, painful leg, Day 9: left-sided weakness, confusion, headache, nausea	7	Aspirin, transfer to Hyper Acute Stroke Unit, IVIG, methylprednisolone, transfer to Neurosciences Intensive Care Unit, argatroban, switch to fondaparinux, decompressive hemicraniectomy, 1U platelets, bone flap removal, placed in a SC abdominal pouch to allow for autologous cranioplasty, CT head: stable appearances of haemorrhagic focus, extubated on post-operative day 2, transfer to stroke ward for ongoing rehabilitation on post-operative day 8	Examination: GCS E4V4M6, dense left-sided hemiparesis, positive NIHSS Score of 15, CT: large volume of hypodensity corresponding to MCA, early resultant mass-effect with 6mm midline shift, CT angiogram: complete occlusion of right M1 segment of MCA, transthoracic echocardiogram: no thrombus, CT angiogram of neck (day 2 of admission): large intraluminal thrombus extending superiorly from origin of right internal carotid artery (4 cm), moderate to severe stenosis, repeat CT brain (day 2 of admission): increasing mass effect, interval CT: increase	Alive

														in degree of midline shift, evidence of haemorrhagic transformation of infarcted region	
Khuhapinant	Kh-1	F	26	Asian	Thailand	Yes	No	NA	NA	NA	severe headache, multiple discrete reddish spots at both lower legs without bleeding gums or epistaxis	3	IVIG, apixaban, dexamethasone	Examination: mildly pale conjunctiva, petechiae at both legs, MRI, angiography, venography of Brain: normal without evidence of thrombosis, CT angiography of pulmonary arteries: no PE, CT of abdomen: unremarkable, no evidence of thrombosis	Alive
Mauriello	Ma-1	F	48	Caucasian	Italy	Yes	allergy to penicillin and a previous episode of thrombocytopenia in 2016	NA	NA	NA	progressive headache, back pain, moderate right lower limb pain, and disseminated ecchymosis (needed hospitalization on day >18 for 7 days)	1	LMWH, transfer to neurology intensive care unit, dabigatran, rivaroxaban, methylprednisolone, decompressive craniectomy	Examinations: apyretic, good hemodynamic compensation, absence of abnormal sounds at respiratory auscultation, soft abdomen, normal neurological objectivity, BP: 214/125mmHg, CTA: right internal jugular vein thrombosis, right transverse sinus thrombosis, massive hemorrhagic focus on right hemisphere, pulmonary angio-CT: thrombo-embolic in pulmonary artery, no densitometric changes in arenchyma, venous echo-color doppler of lower limbs: absence of previous/concurrent deep venous obstructions, emergency skull CT: massive right temporo-occipital intraparenchymal hemorrhage (to ventricular system/midline shift), CT angiography: intra-/extra-cranial circulation: thrombotic phenomena of sigmoid transverse sinus/right internal jugular vein, massive cerebral hemorrhage, purulent abscess, right frontotemporo-parietal lobes, nucleus of right base, midline shift/wedging of cerebellar tonsils, internal/external haemocephalus, bilateral confluent foci of bronchopneumonia associated to right apical	Died

														pulmonary infarction of both lungs, TEM of lung: confirmed frequent occurrence of vascular septic thrombi, microvascular fibrin deposition, long standing petechiae, paradoxical effect in lung, diffuse pulmonary thrombosis	
De Michele	Mi-1	F	57	NA	Italy	Yes	mild hypothyroidism, previously treated breast cancer (surgically, now in follow-up care) with normal mammography and breast ultrasound	No	NA	NA	hemiplegia, right gaze deviation, dysarthria, left neglect, caused by right MCA occlusion	9	mechanical thrombectomy, platelet transfusion, a second successful endovascular treatment, Decompressive craniectomy, betamethasone, IVIG, fondaparinux, control thorax CT: widespread ground-glass attenuation (severe acute respiratory distress syndrome)	Brain MR: right MCA occlusion, MCA occlusion, malignant infarct (ischemia in whole MCA, space-occupying cerebral edema, rapid neurological deterioration), re-occlusion of right MCA, total body CT: extensive pulmonary artery/portal vein thrombosis, no evidence of underlying malignancy, TTE: structurally normal heart, transcranial color Doppler ultrasonography with bubble test: negative for right-to-left shunt	Alive
	Mi-2	F	55	NA	Italy	Yes	mild hypothyroidism	No	NA	NA	abdominal pain	7	Orotracheal intubation, transfer to ED, IVIG, dexamethasone	Abdominal ultrasound: normal, transient episode of aphasia/right hemiparesis, generalized seizures, coma, Brain CT scan, angio-CT, perfusion CT: occlusion of right internal carotid artery terminus, left MCA, extensive ischemic cores, severe bilateral hypoperfusion, without treatable penumbra, TTE: normal. total body CT: extensive portal vein thrombosis, occlusion of left intrahepatic branches, left lower lobe subsegmental pulmonary arteries thrombosis, brain CT: bilateral malignant MCA infarct, uncal herniation	Died
Panovska-Stavridis	PS-1	F	29	Caucasian	Macedonia	Yes	No	NA	None	NA	Day 0: fever, Day 9: flu-like symptoms and fever, Day 10: severe headache, left	9	IVIG, broad-spectrum antibiotics, direct oral anti-Xa inhibitor, Rivaroxaban, prednisolone	Examination: left eyeball swelling, proptosis, limited ocular motility, diplopia at vertical gaze, head CT head: normal, contrast-enhanced MRI: central filling defects, hyper T2 signal in left superior	Alive

											orbital swelling, blurred left eye vision			ophthalmic vein (SOV) thrombosis, MRI: thrombosis	
Patriquin	Pat-1	F	45	NA	Canada	NA	No	NA	NA	NA	Back pain, hypotension, reduced LOC, adrenal shock, left renal vein thrombosis and infarct, bilateral adrenal thrombosis and hemorrhage, common iliac thrombosis, subsegmental PE, right vertebral artery occlusion and left internal carotid artery thrombosis	11	TPE (full plasma), Argatroban, rituximab, fondaparinux, prednisone	iliac vein thrombosis, left renal infarct, bilateral adrenal hemorrhage, subsegmental PE, right vertebral artery, left internal carotid artery thromboses, CT and MRI-brain: dural venous sinus thromboses, transverse, sigmoid, superior sagittal sinuses, repeat CT: superior sagittal sinus thrombosis, right-sided cortical vein, Repeat CT: extension of superior sagittal sinus thrombosis	Alive
	Pat-2	F	46	NA	Canada	NA	No	NA	NA	NA	Headache, photophobia, nausea/vomiting, fever and chills, dural venous thrombosis : right transverse/sigmoid sinuses, internal jugular vein/along superior sagittal sinus, CVST; transverse, sigmoid, superior sagittal sinuses	10	TPE (half Plasma), half albumin, Argatroban, fondaparinux, IVIG, prednisone, apixaban	Examination: no neurologic deficits, CT/MRI-brain: dural venous sinus thromboses, transverse, sigmoid, superior sagittal Sinuses, CT: stable thrombosis with possible minimal clot retraction	Alive
	Pat-3	F	48	NA	Canada	NA	Previously treated breast cancer, Resected brain metastasis, previous whole-brain radiotherapy, secondary	NA	tamoxifen, pravastatin, citalopram, and levetiracetam	NA	Left calf pain, Cold/numb limb, cyanotic toes, paresis unable to extend/flex foot, left subclavian artery	16	Heparin, transfer to Vascular Medicine service, TPE (full plasma), Argatroban, above knee amputation, no further thromboses, rivaroxaban,	imaging: left subclavian artery thrombus, thoracic/abdominal aortic thrombus, total occlusion of right internal iliac artery, multiple thrombi in left lower limb arteries, brain imaging: normal, no sign of cancer, repeat CT: new arterial thrombi in both	Alive

							seizures, previous metastatic breast cancer				thrombus, thoracic/ abdominal aortic thrombus, right internal iliac artery total occlusion, multiple thrombi in left lower limb arteries, Takotsubo cardiomyopathy			legs, progression of abdominal aortic thrombosis, no venous thrombi, CT after last TPE: slightly more prominent thrombus in right internal iliac artery, no other significant changes	
Paul	Pal-1	M	middle aged	NA	India	Yes	NA	NA	NA	NA	severe abdominal pain, increased bruising, hematuria, episodes of transient ischemic attacks	5	steroid pulse (methyl prednisolone-5 days), oral steroids, daily plasma exchange [FFP], methylprednisolone pulse, intubation and mechanical ventilation, anti-epileptic agents, rituximab, LMWH, ecosprin, Antithrombotic/anticoagulation therapy was deferred and managed with conservative measures	Neuroimaging studies, EEG: normal, recurrent seizure, altered sensorium episodes, worsening hemolytic anemia and renal dysfunction, sonogram of right forearm: possible multiple deep muscular hematomas in fore-arm/surrounding edema	Alive
Ramessur	R-1	M	73	NA	UK	Yes	Atrial fibrillation with ischaemic cardiomyopathy	NA	several longstanding medications, including apixaban	NA	Day 0: unwell, fever, headache, Day 3: left shin erythema and blistering, with rapidly ulceration	0	topical clobetasol propionate 0.05%, neomycin sulfate and nystatin ointment, compression bandaging treatment, second dose: switched to Pfizer	Examination: two superficial ulcers (necrotic base/violaceous edge) on lateral aspect of left shin, punch biopsy from edge of ulcer: microthrombi in blood vessels, ischaemic epidermis/fat necrosis of subcutaneous tissue	Alive
Soleimani	Sol-1	M	34	NA	UK	NA	Previously lithium-treated bipolar disorder	No	NA	NA	Day 4: constant, holocephalic headache, Day 14: drowsiness, agitation, right sided weakness, Day 12: photophobia, vomiting	4	Intubation, transfer to ICU, platelets, plasma exchange, IVIG, argatroban, decompressive hemicraniectomy, external ventricular drain, antibiotics, apixaban, off ventilation with a tracheostomy	Examination: GCS 10, equal/reactive pupils, dense right sided hemiparesis, multiple discrete petechiae on legs, bruise on left shoulder, brain CT: superior sagittal sinus hyperdensity intraparenchymal haemorrhage in left perirolandic region, CT venogram: CVST in the superior sagittal sinus extending to both transverse venous sinuses, and thrombosis in the left vein	Alive

														of Trolard, whole body CT: right lower lobe segmental PE, repeat CT brain: worsened cerebral oedema with right sided midline shift/early uncal herniation. right lower lobe segmental PE	
	Sol-2	F	59	NA	UK	NA	Previous lumbar discectomy	No	NA	NA	headache, jaw pain, left-hand weakness, subconjunctival haemorrhage, abdominal pain	11	Aspirin, clopidogrel, levetiracetam, IVIG, methylprednisolone, transfer to ICU for intubation/ventilation, platelets, uncomplicated hemicraniectomy and intracerebral clot evacuation using imaging guidance, cryoprecipitate, surgery, argatroban	Examination: left pronator drift, initial non-contrast CT head: normal, MRI head: loss of flow void in anterior aspect of superior sagittal sinus, susceptibility artefact on gradient echo, cortical vein thrombosis with adjacent cortical haemorrhage, small area of restricted diffusion around precentral gyrus, suspicious for haemorrhagic infarction, MR venogram: thrombosis in superior sagittal/right transverse sinuses, substantial progression of right haemorrhagic infarction to a frontal haematoma associated with midline shift, repeat non-contrast CT after intubation: worsening cerebral oedema secondary to haemorrhage with associated midline shift, post-operative CT: satisfactory evacuation of right intraparenchymal clot, whole body CT: right lower lobe PE, thrombosed right hepatic vein (impaired liver function/abdominal pain)	Alive
	Sol-3	F	39	NA	UK	Yes	No	No	NA	NA	right calf pain, cold sensation of limb, left temporal headache, severe headache, photophobia, phonophobia, nausea, petechial rash on legs and abdomen	10	transfer to local stroke unit, IVIG, fondaparinux, Argatroban, prednisolone, plasma exchange, apixaban	Examination: mild photophobia, cranial nerve/motor/sensory examination: normal, Examination: mild photophobia, CT venogram: left transverse/sigmoid sinus thrombosis, sagittal sinus/thrombosed left jugular vein	Alive
Sørensen	Sor-1	F	30	NA	Denmark	Yes	Migraines	No	OCP	NA	Day 8: headache,	8	Tinzaparin, fibrinogen substitution,	Unremarkable examination, BMI: 22 kg/m2, thrombelastography:	Alive

											general malaise<br>Day 11: persistent headache, ecchymosis		fondaparinux, rivaroxaban	normal clot initiation, decreased clot amplification/strength, analysis of cerebrospinal fluid: meningitis rule-out, noncontrast head CT: normal, MRI/venogram: rule out of intracranial pathology, including CVST, Duplex ultrasonography of lower extremities/CT pulmonary angiography: normal, duplex ultrasonography of abdomen/confirmatory CT abdominal angiography: PVT, repeated ultrasonography: no progression of PVT, cerebral CT venography: newly developed CVST, no signs of cerebral venous stasis, infarction, or bleeding, follow-up CT venography: regression of CVST/PVT	
Suresh	Sur-1	m	27	NA	UK	Yes	No	NA	NA	NA	intermittent headaches, eye floaters, vomiting	2	Dabigatran, IVIG, idarucizumab, Prednisolone, high-dose steroids, PPI, intubation, emergency decompressive craniotomy, external ventricular drain	Head CT: normal, CT venogram: CVST, repeat head CT: acute parenchymal haemorrhage in right parietal lobe	Died
Tejpal	Tej-1	M	61	NA	Canada	Yes	HTN, DLP, CAD, OSA, previous total hip arthroplasty, prior prostate resection, spinal stenosis,<br>up to date with cancer screening	No	telmisartan, rosuvastatin, bisoprolol, aspirin, clopidogrel, pantoprazole, and nabilone	NA	Day 20: back pain<br>Day 26: mechanical back pain<br>Day 29: shortness of breath, hemoptysis, presyncope	20	Dalteparin, apixaban, IVIG	BP: 128/79, HR: 90 bpm, O2Sat: 94%, no calf pain/edema, Electrocardiogram: new T-wave inversions in anterior leads, Chest CT: massive bilateral PE/saddle embolus, Brain CT venogram: normal, bilateral doppler ultrasounds of lower extremities: acute right leg DVT, occlusion of right middle/distal popliteal vein, peroneal/posterior tibial veins	Alive
Tiede	Ti-1	F	63	NA	Germany	Yes	NA	No	NA	NA	headache, somnolence, dysphasia, right sided hemiparesis, arterial hypertension	11	Heparin<br>Eculizumab	CVST, progressive signs of TMA: Coombs-negative hemolytic anemia, abundant schistocytes, renal failure. Imaging: Left transverse/sigmoid sinus thrombosis. Left temporal bleeding	Alive

	Ti-2	F	67	NA	Germany	Yes	NA	No	NA	NA	Headache	8	Argatroban<br>IVIG	Arterial cerebral embolism, cortical infarctions, aortic arch thrombi	Alive
	Ti-3	F	41	NA	Germany	Yes	NA	No	NA	NA	Headache, diplopia	5	Argatroban	Headache/visual disturbance, Imaging on admission: No pathology. TIA	Alive
	Ti-4	F	61	NA	Germany	Yes	NA	No	NA	NA	Fatigue	9	Argatroban, IVIG, Alteplase (day 6), Eculizumab (day 7)	Imaging: no pathology on admission.Day 6: SVT post admission	Alive
	Ti-5	F	61	NA	Germany	Yes	NA	No	NA	NA	Headache, dysarthria, left sided hemiplegia, conjugated gaze palsy	9	Argatroban<br>IVIG	Arterial cerebral thrombosis, popliteal artery thrombosis, right internal carotid/MCA (M1) thrombosis, right MCA territory infarction with hemorrhagic transformation	Alive
Turi	Tu-1	F	57	NA	Italy	Yes	No	NA	NA	NA	Day 2: fever, arthromyalgia, headache, (persistence till 6th day) Day 11: f lu-like symptoms, purpuric lesions on both legs	2	IVIG, methylprednisolone, prednisone, gabapentin, morphine, fondaparinux, dexamethasone, dabigatran, acetylsalicylic acid.	CT: no vascular or parenchymal alteration, Contrast-enhanced cerebral, chest/abdomen CT: large thrombosis in abdominal aorta, Contrast CT of chest/abdomen: PE, PVT, splenic artery thrombosis, splenic infarction, parietal thrombus in thoracic aorta, increased size of thrombus in abdominal aorta	NA
Varona	Var-1	M	47	NA	Spain	NA	No	NA	NA	NA	bilateral segmentary pulmonary embolism (without hemodynamic compromise)	10	LMWH, intravenous immunoglobulins, fondaparinux, hydrocortisone	bilateral segmentary pulmonary embolism (without hemodynamic compromise), cranial CT/MRI: CVT in several locations, cranial MRI: partial revascularization of superior sagittal cerebral venous sinus, abdominal MR: bilateral adrenal nodular enlargement with hyperintense peripheral halo and hypointense center, corresponding to ongoing subacute bilateral adrenal hemorrhage, non-massive PE, CVT/primary adrenal insufficiency due to bilateral adrenal hemorrhage in setting of VITT	Alive
Vayne	Vay-1	F	43	NA	Singapore	NA	NA	No	NA	NA	CVT,	10	NA	CVT,	NA
	Vay-2	F	69	NA	Singapore	NA	NA	No	NA	NA	CVT, Splanchnic VT	9	NA	CVT, Splanchnic VT	NA

	Vay-3	F	38	NA	Singapore	NA	NA	No	NA	NA	CVT, Splanchnic VT	14	NA	CVT, Splanchnic VT	NA
	Vay-4	F	24	NA	Singapore	NA	NA	No	NA	NA	CVT, Splanchnic VT	18	NA	CVT, Splanchnic VT	NA
	Vay-5	F	73	NA	Singapore	NA	NA	No	NA	NA	Ischemia stroke, DVT	10	NA	Ischemia stroke, DVT	NA
	Vay-6	M	23	NA	Singapore	NA	NA	No	NA	NA	CVT	13	NA	CVT	NA
	Vay-7	M	63	NA	Singapore	NA	NA	No	NA	NA		14	NA	None	NA
	Vay-8	F	21	NA	Singapore	NA	NA	No	NA	NA	CVT, Splanchnic VT, DVT, PE	9	NA	CVT, Splanchnic VT, DVT, PE	NA
	Vay-9	F	60	NA	Singapore	NA	NA	No	NA	NA	Splanchnic VT, PE, aortic thrombosis	9	NA	Splanchnic VT, PE, aortic thrombosis	NA
Wang	Wan-1	F	41	NA	Taiwan	Yes	No	NA	NA	NA	fever, headache, pain, swelling erythema on bilateral palms, erythromelalgia	0	IVIG, DOAC, steroid	brain MRI: focal T1 intermediate-signal-intensity/T2 hyper-signal-intensity lesion in posterior superior sagittal sinus, CVST Chest CT: PE at left pulmonary artery/segmental branches	Alive
Wiedmann	Wi-1	F	34	NA	Norway	Yes	Pollen allergy	No	Contraceptive vaginal ring	NA	headaches, left-sided limb weakness, dysarthria	7	NA	Examination: drowsy, dysphasic, left sided hemiparalysis, vertical gaze deviation, Cerebral CT: large right-sided parenchymal/SAH, increased parenchymal hemorrhage,mass effect/herniation. Autopsy: edematous brain, sparse SAH, large hemorrhagic infarction in right hemisphere, thrombi in both transverse sinuses, scattered petechial/ flame-shaped hemorrhages on the skin, peritoneal membranes, mucosal surfaces. No thrombi in peripheral veins/extracerebral organs	Died
Zanferrari	Z-1	F	40	NA	Italy	Yes	Previous regular delivery, previous spontaneous abortions (5 years ago and a week before	NA	NA	NA	Day 0: fever, headache, diffuse joint pain Day 7: headache Day 10: worsening of headache,	0	Enoxaparin, fondaparinux, IVIG	Normal neurological examination, Brain CT: hyperdensity of the left transversal sinus without parenchymal lesions, MRI: extended thrombosis; left-side sigmoidal/transversal sinuses, rectus/inferior longitudinal sinuses	Alive

							vaccination with AZ1222)				nausea, vomiting			without parenchymal damages, Chest X-ray, thoracic CT, electrocardiogram, echocardiogram, cardiological evaluation: negative, Brain CT: left-side temporal-occipital hypodensity with haemorrhagic infarctions	

*Two patients in Greinacher9 had an underlying coagulation disorder (1 patient, von Willebrand disease; 1 patient, “unspecified coagulation disorder”)

** Additional thrombosis events associated with progression occurred in patients who received platelet transfusions or heparin-based therapy at presentation. with no history of a medical condition or use of a medication likely to precipitate thrombosis, except for 1 patient who had a history of deep venous thrombosis and 1 patient who was known to be taking the combined oral contraceptive pill

***There was no particular history or risk factor apart from long term well tolerated oral estroprogestative contraception in 4 patients.

**** 10 patients (patient codes: G11-2 to G11-11) were found to have one or more thrombotic complications beginning 5 to 16 days after vaccination with ChAdOx1 nCov-19. The numbers in this part, represent onset (day) of symptoms.

***** Mild bruising and petechiae were evident in some patients. Secondary cerebral hemorrhage was noted in some patients who had cerebral venous thrombosis. The 1 patient who did not present with thrombosis had clinically significant bruising but no other hemorrhagic manifestations. The numbers in this part, represent onset (day) of symptoms. headaches, fevers, and muscle aches have occurred after vaccination for 48 to 72 hours in some patients.

****** an additional eight patients were identied who developed one or more thrombotic complications beginning four to 16 days following vaccination with AZD1222. The numbers in this part, represent onset (day) of symptoms.

******* The numbers in this part, represent onset (day) of symptoms, however, the type of initial symptom has not mentioned in this study.

Table S1b. Summary of patient characteristics, therapeutic measures, and outcome of case reports on VITT after ChAdOx1 nCov-19 vaccination (total number of cases=167, NAD: no data available)

1st dose	Past medical history (PMH)	Smoker/non-smoker	Medication on admission	Previous exposure to heparin (PEH)	Chief complaint/ initial symptom	Medical examination, imaging method, site of thrombosis/hemorrhages	Therapeutic measures	Outcome
NAD (n=75), 1st dose (n=92)	NAD (n=27), no remarkable PMH (n=100), remarkable PMH (n=40: von Willebrand disease, unspecified coagulation disorder, demyelinating disease, previous DVT, pollen allergy, asthma, HTN, CND, anticardiolipin/antiphospholipid antibodies, FVL, Hashimoto thyroiditis, PSC, migraines, heterozygous MTHFR mutation, Meniere's disease, DM, DLP, high liver enzymes, obesity, overweight, personal and family history of rare congenital limb deformity, fibromyalgia, OSA, prostate cancer, diverticulitis, cardiomyopathy, secondary polycythemia, depression, previous Guillain Barre, previous COVID-19 infection, PCOS, BRCA2 carrier, allergy to penicillin and a previous episode of thrombocytopenia, hypothyroidism, previously treated breast cancer (non-metastatic/metastatic), previously resected brain metastasis and whole brain radiotherapy, secondary seizures, atrial fibrillation with ischaemic cardiomyopathy, previously lithium-treated bipolar disorder, previous lumbar discectomy, CAD, previous total hip arthroplasty, previous prostate resection, spinal stenosis, previous spontaneous abortions)	NAD (n=156), Non-smoker (n=7), Smoker (n=3), Ex-smoker (n=1)	NAD (n=81), no regular medications (n=38), regular medication (n=48: contraceptives, hormone-replacement therapy, antihypertensive, lipid-lowering medications, thyroid medications, ursodeoxycholic acid, budesonide, sumatriptan, amitriptyline, anti-diabetics, aspirin, anti-depressives, tamoxifen, citalopram/levetiracetam, apixaban, clopidogrel, bisoprolol, pantoprazole, nabilone)	No PEH (n=72), PEH (n=2), NAD (n=93)	NAD (n=29), reported symptoms (n=138: flu-like symptoms (1.45%), fatigue (2.9%), cough (0.72%), arthromyalgia/myalgia (>4.35%), backache (5.07%), neckache (0.72%), neck stiffness (0.72%), headache (>44.2%), chills (6.52%), fever (>10.87%), nausea (12.32%), vomiting (8.7%), malaise/unwell feeling (3.62%), somnolence/drowsiness (5.07%), confusion (2.17%), hematemesis (0.72%), epigastric discomfort (1.45%), abdominal pain/cramps (6.52%), hematuria (0.72%), bleeding of his gums (0.72%), jaw pain (0.72%), slight deviation of the right buccal rim (0.72%), diffuse joint pain (0.72%), agitation (0.72%), tingling in arm (0.72%), discoordination/weakness/numbness/hemiparesis/cyanotic toes (19.6%), shin erythema/blistering/ulceration (1.45%), claudication/cramping/cool/painful/swelling limb (8.7%), eye pain (0.72%), conjunctival congestion (0.72%), retro-orbital pain (0.72%), left orbital swelling (0.72%), diplopia (1.45%), eye deviation to the right (2.17%), visual disturbance (5.07%), eye floaters (0.72%), subconjunctival hemorrhage (0.72%), hemianopia (0.72%), conjugated gaze palsy (0.72%), photophobia (3.62%), phonophobia (0.72%), ear pain (0.72%), tinnitus (0.72%), adrenal shock (0.72%), hemisensory loss (0.72%), seizures (2.17%), unstable walking (0.72%), dizziness/disorientation (1.45%), stroke/acute cerebrovascular accident (2.9%), hemiplegia (2.17%), decreased motor function (0.72%), chest pain (3.62%), hemoptysis (1.45%), reduced level of consciousness (LOC) (2.17%), aphasia/dysphasia (5.07%), dysarthria (2.9%), neurologic symptoms (2.17%), left neglect (0.72%), MCA occlusion (0.72%), hematological symptoms (0.72%), thrombocytopenia (1.45%), arterial hypertension (0.72%), hypotension (0.72%), facial weakness (1.45%), presyncope (0.72%), dyspnoea (5.8%), hemorrhage/thrombotic problems (22.46%), secondary cerebral hemorrhage (9.42%), infarct/ischemic attacks (1.45%), Takotsubo cardiomyopathy (0.72%), multiple discrete reddish spots at both lower legs (0.72%), ecchymosis (1.45%), bruising (2.17%), petechiae (>2.17%), purpuric lesions (0.72%), thrombophlebitis of the leg (0.72%))	CVST/CVT (n=79), herniation of the brain (n=8), occipital, temporo-occipital, frontal, juxtacortical, cerebellar, intraparenchymal/hemispheric/parenchymal hemorrhage/subarachnoid hemorrhage (SAH) (n=71), occlusion/thrombosis of internal carotid arteries (n=6), SVT (n=53) (PVT, mesenteric, splenic, ileal, or hepatic veins), PE (n=38), transverse sinus/cerebral thrombosis (n=35), sigmoid sinus thrombosis (n=24), superior/inferior sagittal sinus thrombosis (n=21), DIC (n=15), IJVT (n=13), MCA infarct (ischemia/hemorrhagic transformation) (n=12), parenchymal/sparse/cortical/subarachnoid hemorrhage (SAH) (n=11), multiple thrombosis (n=10), aortic arch thrombi/acute/aortic thrombosis (AAT) including infrarenal/abdominal aorta (n=9), DVT (n=10), CoVT (n=11), ICH (n=7), adrenal hemorrhage (n=7), azygos vein thrombosis (n=1), hemiazygos vein thrombosis (n=1), basivertebral/right vertebral veins (n=2), pulmonary infarct (n=2), middle/lower/left/right/bilateral segmental pulmonary artery thrombi (n=2), SOVT (n=2), splenic infarction (n=3), hepatic infarction (n=1), thrombus in infrarenal/thoracic aorta (n=4), intraventricular thrombosis (n=1), iliofemoral vein thrombosis (n=1), TIA (n=1), IVC thrombosis (n=3), renal infarction (n=3), thrombi in iliac arteries/veins (n=5), thrombosis in the left vein of Trolard (n=1), occlusion of straight sinus (n=4), revascularization of superior sagittal cerebral venous sinus (n=1), sagittal sinus hemorrhage (n=1), vein of Galen thrombosis (n=3), cortical infarctions (n=1), thrombolysis in cerebral infarction scale (n=1), superficial anastomotic vein thrombosis (n=1), border zone infarcts (n=2), semicentral ovale without hemorrhagic transformation (n=1), substantial progression of right hemorrhagic infarction to a frontal hematoma (n=1), intraparenchymal/hemorrhagic venous infarction (n=1), Labbè/superior longitudinal sinus thrombosis (n=1), arterial cerebral thrombosis/embolism (n=3), thrombosis of internal cerebral veins (n=1), popliteal artery/vein thrombosis (n=3), dural venous sinus thromboses (n=3), transverse sinus hemorrhage (n=1), thrombosis in the lungs and intestine (n=1, detected postmortem),	NAD (n=68) Reported measures (n=99: IVIG, LMWH; dalteparin, enoxaparin, danaparoid, heparin, tinzaparin, DOACs; dabigatran, apixaban, rivaroxaban, argatroban, aspirin, ecosprin, phenprocoumon , fondaparinux, oral vitamin K antagonist, warfarin, clopidogrel, TPE, albumin, hemodialysis, RBC/platelet transfusion, fibrinogen concentrate, prothrombin complex concentrates, rVIIa, decompressive hemi/craniectomy, ICP bolt insertion, endovascular intervention+thrombectomy, percutaneous coronary intervention, endovascular rheolysis, bone flap removal, neurosurgery, dual thrombo-aspiration (ADAPT), plasmapheresis, FFP, cryoprecipitate, corticosteroids; methylprednisolone, prednisolone, dexamethasone, betamethasone, hydrocortisone, IV antibiotics; cefuroxime, topical clobetasol propionate, neomycin sulfate, nystatin ointment + compression bandaging, antivirals, analgesia, levetiracetam, lacosamide, gabapentin, mannitol, hypertonic saline, PPI, rituximab, idarucizumab, intubation, Eculizumab, ventilation, POBA, aspiration thrombectomy, tPA, alteplase, ionotropic support, surgical embolectomy, amputation, external ventricular drain, off ventilation + a tracheostomy, intracerebral clot evacuation, second dose switch to Pfizer)	NAD (n=11), Death (n=49), full recovery (n=107)

hemorrhagic symptoms only (no thrombosis) (n=1), ischemic bowel with infarction (n=1), peroneal/posterior tibial veins (n=1), thrombus in all tibial arteries (n=1), left superficial and deep femoral arteries (n=1), limb thrombosis/necrosis (n=5), multifocal venous thrombosis (n=1), left/right/bilateral occlusion of parietal cortical veins (n=9), MI (n=3), cardiac arrest (n=1), rectus/inferior longitudinal sinuses (n=2), thrombosis of venous sinuses (n=1), TMA/widespread microvascular (brain, lungs, kidneys) (n=2), microthrombi in blood vessels of shin (n=1), renal hematoma/suprarenal aortic thrombosis (n=2), right renal vein thrombus (n=1), acute intracerebral hematoma (n=1), subcortical hematoma in left parietal lobe (n=1), multiple organ thrombi/ multi-vessel involvement (n=2), large hemorrhagic infarction in right hemisphere (n=2), ischemia stroke (n=2), scattered petechial/flame-shaped hemorrhages on the skin, peritoneal membranes, mucosal surfaces (n=1), uterine veins thrombosis (n=1), acute basilar thrombosis (n=1), occlusion of internal/ascending cerebral veins (n=2), cerebral artery ischemic lesions, perforating pontine branches (n=1), ischemic hypodensity involving right occipito-temporal, superior cerebellar regions, right thalamic, internal capsula regions, pons/mesencephalon (n=1), blood in pelvis (n=1), arterialization of liver parenchyma (n=1), Rutherford IIB acute limb ischemia (n=1), thrombophlebitis (n=1), oedema in brain tissue (n=10), RV thrombus (n=1), acute kidney injury (n=1), ICP (n=1), thromboses of celiac/right peroneal arteries (n=1), thrombosis in proximal circumflex/posterior descending arteries (n=1), coronary artery and left atrial appendage thrombosis (n=1), GI bleeding (n=2), left-temporal/left-cerebellar intracerebral haemorrhage (n=1), purulent abscess, right frontotemporo-parietal lobes nucleus of right base, midline shift/wedging of cerebellar tonsils, internal/external haemocephalus (n=1), microvascular fibrin deposition (n=1), thrombosis of left subclavian artery (n=1), deep muscular hematomas (n=1). no evidence of thrombosis by imaging (n=1)

Table S2. Details of common laboratory blood tests and SARS-CoV-2 screening of case reports on VITT after ChAdOx1 nCov-19 vaccination.

Author	Patient code	Hemoglobin (g/dL) [ꟼ]	White cells (10^9/L) [¥]	Platelets (10^9/L) [£]	Prothrombin time (INR) [ɸ]	Activated partial thromboplastin time (ratio)/aPTT/PTT[€]	Thrombin time (seconds)[ᵖ]	Fibrinogen (mg/dL) [ʒ]	D-dimer (ng/mL) [₵]	Antithrombin[Ω]	C reactive protein (mg/dl)[ƚ]	SARS-CoV-2 screening
Greinacher*	G9-1	12.3	6.6	18	1.4	34	NA	NA	35000	NA	8.8	PCR Negative
	G9-2	NA	NA	100	NA	NA	NA	NA	NA	NA	NA	NA
	G9-3	NA	NA	31	NA	NA	NA	NA	NA	NA	NA	NA
	G9-4	NA	NA	9	NA	NA	NA	NA	NA	NA	NA	NA
	G9-5	NA	NA	23	NA	NA	NA	NA	NA	NA	NA	NA
	G9-6	NA	NA	75	NA	NA	NA	NA	NA	NA	NA	NA
	G9-7	NA	NA	29	NA	NA	NA	NA	NA	NA	NA	NA
	G9-8	NA	NA	60	NA	NA	NA	NA	NA	NA	NA	NA
	G9-9	NA	NA	11	NA	NA	NA	NA	NA	NA	NA	NA
Al-Mayhani	A1	NA	NA	46	NA	NA	NA	NA	11220	NA	NA	NA
	A2	NA	NA	9	NA	NA	NA	NA	34000	NA	NA	NA
	A3	NA	NA	48	NA	NA	NA	NA	24000	NA	NA	NA
Scully	Scu-1	NA	NA	27	1.1	35.0	NA	250	16280	NA	NA	PCR Negative **
	Scu-2	NA	NA	11	13.1	1	NA	110	26689	NA	NA	PCR Negative **
	Scu-3	NA	NA	64	1.1	1.1	NA	320	>5000	NA	NA	PCR Negative **
	Scu-4	NA	NA	31	15.0	35.0	NA	120	37250	NA	NA	PCR Negative **
	Scu-5	NA	NA	16	12.8	30.8	NA	120	45229	NA	NA	PCR Negative **
	Scu-6	NA	NA	14	1.4	36.0	NA	130	39049	NA	NA	PCR Negative **
	Scu-7	NA	NA	19	1.2	1.1	NA	130	NA	NA	NA	PCR Negative **
	Scu-8	NA	NA	87	14.1	26.7	NA	170	NA	NA	NA	PCR Negative **
	Scu-9	NA	NA	65	13.2	28.7	NA	220	10316	NA	NA	PCR Negative **
	Scu-10	NA	NA	NA	13.1	23.0	NA	260	6018	NA	NA	PCR Negative **
	Scu-11	NA	NA	34	NA	NA	NA	210	10388	NA	NA	PCR Negative **
	Scu-12	NA	NA	23	14.8	22.0	NA	70	37000	NA	NA	PCR Negative **
	Scu-13	NA	NA	71	13.5	32.7	NA	120	80000	NA	NA	PCR Negative **

	Scu-14	NA	NA	17	1.4	1.3	NA	80	>20000	NA	NA	PCR Negative **
	Scu-15	NA	NA	100	11.1	23.6	NA	300	>10000	NA	NA	PCR Negative **
	Scu-16	NA	NA	57	1.2	0.9	NA	440 (derived)	>50000	NA	NA	PCR Negative **
	Scu-17	NA	NA	28	1.1	1.4	NA	380 (derived)	>5000	NA	NA	PCR Negative **
	Scu-18	NA	NA	113	1.3	0.8	NA	100	22903	NA	NA	PCR Negative **
	Scu-19	NA	NA	7	1.1	1.1	NA	110	31301	NA	NA	PCR Negative **
	Scu-20	NA	NA	98	1.5	1.7	NA	<40	6574	NA	NA	PCR Negative **
	Scu-21	NA	NA	16	1.2	1.0	NA	120	62342	NA	NA	PCR Negative **
	Scu-22	NA	NA	61	1.3	1.0	NA	450	71859	NA	NA	PCR Negative **
	Scu-23	NA	NA	36	1.4	1.4	NA	70	>20000	NA	NA	PCR Negative **
Schultz	Sch-1 37	12.1	NA	22	1.2	25	NA	210	>35000	76 (negative)	moderately elevated	****
	Sch-2 42	12.1	NA	14	1.0	31	NA	80	>35000	100 (negative)	Normal	Negative
	Sch-3 32	NA	NA	10	1.1	25	NA	230	>35000	negative	moderately elevate	Negative
	Sch-4 39	12.6	NA	70	1.3	25	NA	120	13000	126 (negative)	Normal	Negative
	Sch-5 54	9.6	NA	19	1.1	29	NA	120	>35000	90 (negative)	moderately elevated	Negative
Greinacher	G11-1	12.3	6.6	13	1.40	41.6	NA	78	142000	NA	Elevated	Negative
	G11-2	NA	NA	107	1.12	29.0	NA	568	1800	NA	NA	NA
	G11-3	NA	NA	60	NA	NA	NA	NA	13000	NA	NA	NA
	G11-4	NA	NA	9	1.66	46.6	NA	NA	NA	NA	NA	NA
	G11-5	NA	NA	23	1.25	64.8	NA	173	NA	NA	NA	NA
	G11-6	NA	NA	75	1.05	23.0	NA	NA	2600	NA	NA	NA
	G11-7	NA	NA	29	1.34	45.0	NA	210	>33000	NA	NA	NA
	G11-8	NA	NA	16	NA	NA	NA	NA	NA	NA	NA	NA
	G11-9	NA	NA	13	1.70	46.1	NA	40	21000	NA	NA	NA
	G11-10	NA	NA	8	NA	NA	NA	80	>35000	NA	NA	NA
	G11-11	NA	NA	NA because of death	NA	NA	NA	NA	NA	NA	NA	NA
Bayas	Ba-1	NA	NA	30	NA	NA	NA	NA	NA	NA	NA	NA
Blauenfeldt	Bl-1	14.0	11.1	118	1.0	NA	NA	NA	NA	NA	0.26	NA
Mehta	Meh-1	Normal (146 x10^9/L)	NA	30	NA	NA	NA	140	NA	NA	4.7	Negative

	Meh-2	Normal (148 x10^9/L)	NA	19	NA	NA	NA	130	NA	NA	0.4	Negative
Castelli	Cas-1	NA	NA	20	NA	NA	NA	98	>10,000	NA	0.76	Negative
D'Agostino	D-1	8.7	NA	thrombocytopenia	51%	41	NA	Normal	Elevated	NA	NA	NA
Esba	E-1	NA	NA	143	NA	NA	NA	NA	NA	NA	NA	NA
	E-2	NA	NA	128	NA	NA	NA	NA	NA	NA	NA	NA
Franchini	F-1	14.6	10.87	15	1.19	0.88	23.5	98	>10,000	101	1.76	Negative
Garnier	Gar-1	NA	NA	major thrombocytopaenia	NA	NA	NA	hypofibrinogenemia	NA	NA	15.8[r]	NA
Geeraerts	Ge-1	NA	NA	Thrombocytopaenia	NA	NA	NA	NA	NA	NA	NA	NA
	Ge-2	NA	NA	Thrombocytopaenia	NA	NA	NA	NA	NA	NA	NA	NA
Gras Champel[J]	GC-1	NA	NA	Thrombocytopenia [J]	NA	NA	NA	NA	NA	NA	NA	NA
	GC-2	NA	NA	Thrombocytopenia [J]	NA	NA	NA	NA	NA	NA	NA	NA
	GC-3	NA	NA	Thrombocytopenia [J]	NA	NA	NA	NA	NA	NA	NA	NA
	GC-4	NA	NA	Thrombocytopenia [J]	NA	NA	NA	NA	NA	NA	NA	NA
	GC-5	NA	NA	Thrombocytopenia [J]	NA	NA	NA	NA	NA	NA	NA	NA
	GC-6	NA	NA	Thrombocytopenia [J]	NA	NA	NA	NA	NA	NA	NA	NA
	GC-7	NA	NA	Thrombocytopenia [J]	NA	NA	NA	NA	NA	NA	NA	NA
	GC-8	NA	NA	Thrombocytopenia [J]	NA	NA	NA	NA	NA	NA	NA	NA
	GC-9	NA	NA	Thrombocytopenia [J]	NA	NA	NA	NA	NA	NA	NA	NA
	GC-10	NA	NA	Thrombocytopenia [J]	NA	NA	NA	NA	NA	NA	NA	NA
	GC-11	NA	NA	Thrombocytopenia [J]	NA	NA	NA	NA	NA	NA	NA	NA
	GC-12	NA	NA	Thrombocytopenia [J]	NA	NA	NA	NA	NA	NA	NA	NA
	GC-13	NA	NA	Thrombocytopenia [J]	NA	NA	NA	NA	NA	NA	NA	NA
	GC-14	NA	NA	Thrombocytopenia [J]	NA	NA	NA	NA	NA	NA	NA	NA
	GC-15	NA	NA	Thrombocytopenia [J]	NA	NA	NA	NA	NA	NA	NA	NA
	GC-16	NA	NA	Thrombocytopenia [J]	NA	NA	NA	NA	NA	NA	NA	NA
	GC-17	NA	NA	Normal [J]	NA	NA	NA	NA	NA	NA	NA	NA
	GC-18	NA	NA	Normal [J]	NA	NA	NA	NA	NA	NA	NA	NA
	GC-19	NA	NA	Normal [J]	NA	NA	NA	NA	NA	NA	NA	NA
	GC-20	NA	NA	Normal [J]	NA	NA	NA	NA	NA	NA	NA	NA
	GC-21	NA	NA	Normal [J]	NA	NA	NA	NA	NA	NA	NA	NA
	GC-22	NA	NA	Normal [J]	NA	NA	NA	NA	NA	NA	NA	NA
	GC-23	NA	NA	Normal [J]	NA	NA	NA	NA	NA	NA	NA	NA
	GC-24	NA	NA	Normal [J]	NA	NA	NA	NA	NA	NA	NA	NA
	GC-25	NA	NA	Normal [J]	NA	NA	NA	NA	NA	NA	NA	NA
	GC-26	NA	NA	Normal [J]	NA	NA	NA	NA	NA	NA	NA	NA
	GC-27	NA	NA	Normal [J]	NA	NA	NA	NA	NA	NA	NA	NA
Jones	J-1	NA	NA	36	1.3	NA	NA	140	> 10,0000	NA	NA	Negative
Ramdeny	Ram-1	NA	NA	34	NA	NA	NA	NA	60,000	NA	NA	NA
Wolf	W-1	NA	NA	75	NA	NA	NA	NA	2590	NA	NA	Negative
	W-2	NA	NA	60	NA	NA	NA	NA	22800	NA	NA	Negative
	W-3	NA	NA	92	NA	NA	NA	NA	2120	NA	NA	SARS-CoV-2-XPCR was negative
Xie	X-1	Normal	NA	73	Normal clotting	Normal clotting	Normal clotting	NA	17548	NA	NA	Negative
Muster	Mu-1	NA	NA	37	Normal	Normal	NA	Normal	34000	NA	4.2	Negative
Aladdin	Ala-1	10.4	18.7	94	PT: 45s INR: 4.1	98	NA	0.6	>35000	NA	NA	Negative
Althaus	Alt-1	NA	NA	10	1.30	23	NA	128	> 35000	NA	NA	NA
	Alt-2	NA	NA	40	1.16	22.9	NA	NA	NA	NA	NA	NA
	Alt-3	NA	NA	22	1.20	42	NA	109	NA	NA	NA	NA

	Alt-4	NA	NA	8	1.01	25	NA	126	>35000	NA	NA	NA
	Alt-5	NA	NA	56	1.25	35	NA	263	9000	NA	NA	NA
	Alt-6	NA	NA	71	NA	NA	NA	NA	NA	NA	NA	NA
	Alt-7	NA	NA	92	1.19	22	NA	NA	13000	NA	NA	NA
	Alt-8	NA	NA	53	1.00	23	NA	274	32000	NA	NA	NA
Bano	Ban-1	NA	NA	25	1.0	1.1	NA	1.26	9376	NA	NA	Negative
	Ban-2	NA	NA	24	1.2	0.9	NA	1.90	5620	NA	NA	Negative
	Ban-3	NA	NA	21	1.1	0.97	NA	1.33	47881	NA	NA	Negative
Bonato	Bon-1	NA	NA	134	Normal	Normal	NA	Normal	12204	Normal	NA	NA
Bourguignon	Bour-1	NA	NA	39	NA	NA	NA	237	2240	NA	NA	NA
	Bour-2	NA	NA	36	NA	NA	NA	140	> 10,000	NA	NA	NA
	Bour-3	NA	NA	35	NA	NA	NA	200	H> 20,000	NA	NA	NA
Choi	Cho-1	17.8	7.40	14	14	28.0	NA	77	> 35200	110.7%	0.342	Negative
Cliff-Patel	CP-1	NA	Mildly elevated	Thrombocytopenia	NA	NA	NA	NA	>20,000	NA	NA	NA
	CP-2	NA	NA	Thrombocytopenia	NA	NA	NA	NA	Elevated	NA	NA	NA
	CP-3	NA	NA	Thrombocytopenia	NA	NA	NA	NA	Elevated	NA	NA	NA
Gangi	Gan-1	NA	NA	8	Normal	Normal	NA	150	5370	NA	NA	Negative
	Gan-2	NA	NA	37	Normal	Normal	NA	210	5690	NA	NA	Negative
	Gan-3	NA	NA	111	Normal	Normal	NA	360	19500	NA	NA	Negative
	Gan-4	NA	NA	18	Normal	Normal	NA	10	66000	NA	NA	Negative
	Gan-5	NA	NA	117	Normal	Normal	NA	210	5420	NA	NA	Negative
	Gan-6	NA	NA	64	Normal	Normal	NA	NA	NA	NA	NA	Negative
Gattringer	Gatt-1	NA	NA	84	NA	NA	NA	236	14200	NA	0.13	Positive
	Gatt-2	NA	NA	29	NA	NA	NA	95	>33000	NA	1.67	Negative
Gessler	Gess-1	NA	NA	9	PT: 10.7s 1.3	23.0	20.9	128	35200	NA	NA	NA
	Gess-2	NA	NA	24	PT: NA INR: 1.44	28	NA	110	35000	NA	NA	NA
Graf	Gra-1	NA	NA	32	NA	50	NA	NA	65700	NA	NA	NA
Guan	Gua-1	NA	NA	99	NA	NA	NA	NA	>20000	NA	NA	NA
Huang	H-1	NA	NA	34	12.8 s	31.9 s	12.8	60	>10000	NA	NA	NA
Ikenberg	Ik-1	NA	NA	37	NA	NA	NA	NA	12859	NA	8.1	Negative
Jacob	Ja-1	NA	NA	66	NA	NA	NA	NA	>5000	NA	NA	Negative
Khuhapinant	Kh-1	9.7	3.66	22	11.9s	25.8	NA	173.8	9452	NA	NA	Negative
Mauriello	Ma-1	NA	NA	32	NA	NA	NA	NA	> 10000	NA	NA	NA
De Michele	Mi-1	5.4	NA	44	Normal	Normal	Normal	NA	High	NA	Normal	Negative
	Mi-2	Normal	Normal	133	Normal	Normal	Normal	NA	5441	NA	Normal	Negative
Panovska-Stavridis	PS-1	NA	NA	18	Normal	Normal	Normal	250	35712	NA	14.2	Negative
Patriquin	Pat-1	13.2	NA	53	1.3	40.1	NA	322	>35200	NA	NA	NA
	Pat-2	15.1	NA	16	1.3	31	NA	120	>44000	NA	NA	NA
	Pat-3	14.6	NA	37	1.2	24	NA	100	>9999	NA	NA	NA
Paul	Pal-1	<12	NA	< 50	NA	NA	NA	NA	NA	NA	NA	Negative
Ramessur	R-1	NA	NA	112	Normal	Normal	Normal	Normal	Normal	Normal	Normal	Negative
Soleimani	Sol-1	15.2	11.15	23	PT: 14.8 INR: 1.5	23.9	NA	70	37293	NA	NA	Negative
	Sol-2	NA	Lymphopenia	21	PT: 14.5 INR: 1.2	24.4	NA	200	35588	NA	NA	Negative
	Sol-3	13.2	12.9	255 (then dropping to 86 and 63)	PT: 15.1 INR: 1.1	33.2	NA	320	20289	NA	Normal	Negative
Sørensen	Sor-1	13.4	11.0	57	INR: 1	38	NA	78	20000	0.99	3.6	Negative
Suresh	Sur-1	15.5	12.0	90	12.9	27.5	NA	194	34071	NA	0.147	NA
Tejpal	Tej-1	NA	NA	134	1.2	29	NA	345	6000	NA	NA	NA
Tiede	Ti-1	NA	NA	27	NA	NA	NA	NA	> 35200	NA	NA	Negative

	Ti-2	NA	NA	40	NA	NA	NA	NA	>035200	NA	NA	Negative
	Ti-3	NA	NA	105	NA	NA	NA	NA	22400	NA	NA	Negative
	Ti-4	NA	NA	12	NA	NA	NA	NA	>35200	NA	NA	Negative
	Ti-5	NA	NA	62	NA	NA	NA	NA	>35200	NA	NA	Negative
Turi	Tu-1	14.9	7.3	120	NA	NA	NA	458	1310	Normal	419.9	NA
Varona	Var-1	NA	NA	51	NA	NA	NA	NA	20506	NA	NA	NA
Vayne	Vay-1	NA	NA	29	NA	NA	NA	179	>20000	NA	NA	NA
	Vay-2	NA	NA	34	NA	NA	NA	200	>4000	NA	NA	NA
	Vay-3	NA	NA	35	NA	NA	NA	80	>4000	NA	NA	NA
	Vay-4	NA	NA	303	NA	NA	NA	380	4000	NA	NA	NA
	Vay-5	NA	NA	22	NA	NA	NA	45	>20000	NA	NA	NA
	Vay-6	NA	NA	25	NA	NA	NA	150	>20000	NA	NA	NA
	Vay-7	NA	NA	13	NA	NA	NA	334	NA	NA	NA	NA
	Vay-8	NA	NA	61	NA	NA	NA	200	NA	NA	NA	NA
	Vay-9	NA	NA	9	NA	NA	NA	60	105000	NA	NA	NA
Wang	Wan-1	NA	NA	36	1.08	NA	NA	213	> 10 000	NA	NA	Negative
Wiedmann	Wi-1	13.6	NA	33	INR: 1	28	NA	190	16200	NA	1	Negative
Zanferrari	Z-1	NA	NA	43	NA	24.9	NA	Negative	27546	Negative	NA	Negative

J Fourteen of the 27 cases met the level 1 criteria of the Brighton collaboration, with a median (range) trough platelet count of 25 G/L (9—61)

r measured after ADAPT technique

Ɑ] Ref: 12.0-16.0 g/dL (Greinacher—2021), 11.8–15.3 g/dL (Blauenfeldt-2021), 133–167 x10^9/L (Mehta-2021), 13.5–17.5 g/dl (Franchini—2021), 13.0–18.0 g/dl (Choi, 2021), 11.7-15.3 g/dl (Wiedmann, 2021), 11.8-15.3 g/dl (Sørensen, 2021), 12-16 g/dl (Turi, 2021)

¥ Ref: 4000 - 10000 per mm^3 (Greinacher—2021), 3500–10,000 per mcL (Blauenfeldt-2021), 4.40–11.0 *10^9/L (Franchini—2021), 4.0–10.0 /mm3 (Choi, 2021), 3.-8.8*10^9/L (Sørensen, 2021), 4.8-10.8*10^3/mcL (Turi, 2021)

£ Ref: 150,000–350,000 per mm3 (Greinacher—2021), 150–400x 10^9/L (Al-Mayhani-2021), 165,000–400,000 per µl (Blauenfeldt-2021), 150-400 cells×10^9/liter (scully-2021, Mehta-2021), 150–400 10^9/L (Castelli-2021, Franchini—2021), 150–400 × 10^9/L (Jones-2021), 150-450x10^9/L (Althaus, 2021), 150–450*10^9 (Bano, 2021), 140-450/mm^3, 140-440*10^9 (Gattringer, 2021), 150–370 and 150-450*10^9 (Gessler, 2021), 150-450K (Mauriello, 2021), 145-390*10^9/l (Wiedmann, 2021), 150–450*10^9/L (Ramessur, 2021), 150-400*10^9/L (Soleimani, 2021), 145-390*10^9/L (Sørensen, 2021), 150–400*10^9/L (Wang, 2021), 130-400 (Turi, 2021)

ɸ Ref: 10-12 sec, <1.1 INR, as ratio: 1.0 -1.2 (Scully 2021), 0.9-1.1 (Greinacher 2021), <1.2 (Blauenfeldt-2021), 0.88–1.12 INR (Franchini—2021), 0.9-1.1 INR (Jones-2021), 0.80–1.20 peak (Bano, 2021), 10.1–14.0 sec (Choi, 2021), PT 7.6-9.8s (Gessler, 2021), PT: 9.8–12.9 s (Khuhapinant, 2021), INR: 0.9-1.2 (Wiedmann, 2021), INR: CASE 1: 0.9 - 1.1, CASE 2: 0.9 - 1.2, CASE 3: < 1.2 (Patriquin, 2021), PT: 9-12 sec, INR: 9-5 IU (Soleimani, 2021), 0.9–1.1 (Wang, 2021)

€ Ref: 25.0-37.0 sec, APTT ratio 0.8 to 1.2 (Scully 2021), 22-38 sec (Blauenfeldt-2021), <35 sec (Greinacher—2021), 0.82–1.18 (Franchini—2021), >40s (Althaus, 2021), 0.80–1.17 peak (Bano, 2021), 23.4–30.3 sec (Choi, 2021), 25-35 and 27-37s (Gessler, 2021), 21.8–30.2 s (Khuhapinant, 2021), 22-30 (Wiedmann, 2021), CASE 1: 18.5 - 29.9, CASE 2: 19 – 28, CASE 3: 22.1 - 29.4 sec (Patriquin, 2021), 20-30 sec (Soleimani, 2021), 25-37s (Sørensen, 2021)

p Ref: 16-20 sec (Franchini—2021), < 20.5 (Gessler, 2021)

ℑ Ref: 170–374 mg/dl (Blauenfeldt-2021), 200–400 mg/dl (Greinacher—2021), 1.9-4.0 g/L (Schultz-2021), 1.5-4.0 g/L (Scully 2021), 1.5–4.5 g/L (Mehta-2021), 150–450 mg/dl (Castelli-2021), 150–450 mg/dl (Franchini—2021), 1.6–4.1 g/L (Jones-2021), 170-410 mg/dL (Althaus, 2021), 1.9–4.8 g/L (Bano, 2021), 160-420 mg/dL (Bourguignon, 2021), 180-415 mg/dL (Choi, 2021), 210-400 mg/dL (Gattringer, 2021), 180-355 mg/dL (Gessler, 2021), 1.9-4.0 (Wiedmann, 2021), 150 – 420, 190 – 450, 230 – 460 mg/dL (Patriquin, 2021), 1.5-4.0 g/L (Soleimani, 2021), 180-350 mg/dl (Sørensen, 2021), 200–400 mg/dl (Wang, 2021), 180-400 mg/dl (Turi, 2021)

ℭ Ref: 0-550 mcg/L (Al-Mayhani-2021), <500 ng FEU/ml (Blauenfeldt-2021), <0.5 mg/L (Greinacher—2021), 0-550 FEU (Scully-2021), < 500 ng/ml (Castelli-2021, Franchini—2021), ≤ 50 ng/mL fibrinogene quivalent units (Jones-2021), <0.5 mg/L (Muster-2021), <0.5 mcg/mL (Althaus, 2021), 0–350 ng/mL (Bano, 2021), <500 mcg/mL (Bonato, 2021), <0.50 mg/L (Bourguignon, 2021), 0–0.55 mg/L (Choi, 2021), <0.50 mg/l (Cliff-Patel, 2021), 0.5 mg/L (Gattringer, 2021), 0-0.5 mg/L (Gessler, 2021), <500 µg/L FEU (Ikenberg, 2021), 0–243 mg/L (Jacob, 2021), < 500 ng/mL (Khuhapinant, 2021), < 0.005 mg/ml (Mauriello, 2021), 0-500 mcg/L (Patriquin, 2021), 0-500 mg/L (Soleimani, 2021), 0.5 mg/L FEU (Sørensen, 2021), 500) ng/ml (Wang, 2021), 0.0-0.5 mg/L (Turi, 2021)

Ω Ref: 0.80–1.20 IU/ml (Blauenfeldt-2021), 75–125 % (Franchini—2021), 60–120% (Choi, 2021), 0.83-1.15 *10^3 IU/L (Sørensen, 2021)

† Ref: <0.8 mg/dl (Blauenfeldt-2021), <0.5 mg/dl (Greinacher—2021), <5 mg/L (Mehta-2021), < 5 mg/L (Castelli-2021, Franchini—2021), <0.5 mg/L (Muster-2021), 0–5 mg/L (Choi, 2021), <0.5mg/L (Gattringer, 2021), <4 mg/l (Wiedmann, 2021), <10 mg/l (Sørensen, 2021), 0.0-5.0 mg/L (Turi, 2021)

** The 10 patients with samples available for testing had a negative SARS-CoV-2 serologic test for antibodies to nucleocapsid protein, a finding that ruled out recent exposure to SARS-CoV-2. In all 10 patients, levels of antibodies to spike protein and RBD of SARS-CoV-2 were within the range seen in recipients of one dose of the ChAdOx1 nCoV-19 vaccine, and levels of antibodies to seasonal coronaviruses were within the range seen in recipients of the ChAdOx1 nCoV-19 vaccine and in the general population. Levels of functional antibodies that inhibit the interaction of ACE2-receptor protein with spike protein and RBD of SARS-CoV-2 were also within the range seen in recipients of one dose of the ChAdOx1 nCoV-19 vaccine (Goldblatt D: personal communication).

**** SARS-CoV-2 antibody test results: Nucleocapsid protein was negative for all patients, and Spike protein was positive for all patients

Table S3. Details of less common laboratory tests of case reports on VITT after ChAdOx1 nCov-19 vaccination.

Patient code	Haptoglobin (mg/dl)ᵽ	RBC (10^12/L)ᵗ	Neutrophil #	Lymphocyte (per µl) §	Monocyte (per µl) ¶	Eosinophil (per µl) æ	Aminotransferase (U/L)ᵵ	GGT** (U/L) ∮	ALP°°°	Sodium (mmol/L) Ǽ	Potassium (mmol/L) ƕ	NU °°	LDH °(IU/L)𝔄	Lactate (mmol/L) 𝕭	Total bilirubin ⁿ	Clotting factor ᴷ	Folic acid (ng/mL)ᑫ	Vit B12 (pg/mL)ᵍ	Pro-calcitonin (µg/L) 𝕯	Cr (mg/dl) ᴻ±±	TP ±	Albumin	PNH □□□	Septic screeen □□	AKI□	Ferritin	Interleukin	Cholesterol HDL RATIO	UREA
G9-1	NA	NAs	NA	NA	NA	NA	AST 33 ALT 46	141	NA	NA	NA	NA	NA	0.9	NA	NA	NA	NA	NA	NA	NA	NA	NA	NA	NA	NA	NA	NA	NA
G9-2	NA	NA	NA	NA	NA	NA	NA	NA	NA	NA	NA	NA	NA	NA	NA	NA	NA	NA	NA	NA	NA	NA	NA	NA	NA	NA	NA	NA	NA
G9-3	NA	NA	NA	NA	NA	NA	NA	NA	NA	NA	NA	NA	NA	NA	NA	NA	NA	NA	NA	NA	NA	NA	NA	NA	NA	NA	NA	NA	NA
G9-4	NA	NA	NA	NA	NA	NA	NA	NA	NA	NA	NA	NA	NA	NA	NA	NA	NA	NA	NA	NA	NA	NA	NA	NA	NA	NA	NA	NA	NA
G9-5	NA	NA	NA	NA	NA	NA	NA	NA	NA	NA	NA	NA	NA	NA	NA	NA	NA	NA	NA	NA	NA	NA	NA	NA	NA	NA	NA	NA	NA
G9-6	NA	NA	NA	NA	NA	NA	NA	NA	NA	NA	NA	NA	NA	NA	NA	NA	NA	NA	NA	NA	NA	NA	NA	NA	NA	NA	NA	NA	NA
G9-7	NA	NA	NA	NA	NA	NA	NA	NA	NA	NA	NA	NA	NA	NA	NA	NA	NA	NA	NA	NA	NA	NA	NA	NA	NA	NA	NA	NA	NA
G9-8	NA	NA	NA	NA	NA	NA	NA	NA	NA	NA	NA	NA	NA	NA	NA	NA	NA	NA	NA	NA	NA	NA	NA	NA	NA	NA	NA	NA	NA
G9-9	NA	NA	NA	NA	NA	NA	NA	NA	NA	NA	NA	NA	NA	NA	NA	NA	NA	NA	NA	NA	NA	NA	NA	NA	NA	NA	NA	NA	NA
A1	NA	NA	NA	NA	NA	NA	NA	NA	NA	NA	NA	NA	NA	NA	NA	NA	NA	NA	NA	NA	NA	NA	NA	NA	NA	NA	NA	NA	NA
A2	NA	NA	NA	NA	NA	NA	NA	NA	NA	NA	NA	NA	NA	NA	NA	NA	NA	NA	NA	NA	NA	NA	NA	NA	NA	NA	NA	NA	NA
A3	NA	NA	NA	NA	NA	NA	NA	NA	NA	NA	NA	NA	NA	NA	NA	NA	NA	NA	NA	NA	NA	NA	NA	NA	NA	NA	NA	NA	NA
Scu-1	NA	NA	NA	NA	NA	NA	NA	NA	NA	NA	NA	NA	NA	NA	NA	NA	NA	NA	NA	NA	NA	NA	NA	NA	NA	NA	NA	NA	NA
Scu-2	NA	NA	NA	NA	NA	NA	NA	NA	NA	NA	NA	NA	NA	NA	NA	NA	NA	NA	NA	NA	NA	NA	NA	NA	NA	NA	NA	NA	NA
Scu-3	NA	NA	NA	NA	NA	NA	NA	NA	NA	NA	NA	NA	NA	NA	NA	NA	NA	NA	NA	NA	NA	NA	NA	NA	NA	NA	NA	NA	NA
Scu-4	NA	NA	NA	NA	NA	NA	NA	NA	NA	NA	NA	NA	NA	NA	NA	NA	NA	NA	NA	NA	NA	NA	NA	NA	NA	NA	NA	NA	NA
Scu-5	NA	NA	NA	NA	NA	NA	NA	NA	NA	NA	NA	NA	NA	NA	NA	NA	NA	NA	NA	NA	NA	NA	NA	NA	NA	NA	NA	NA	NA
Scu-6	NA	NA	NA	NA	NA	NA	NA	NA	NA	NA	NA	NA	NA	NA	NA	NA	NA	NA	NA	NA	NA	NA	NA	NA	NA	NA	NA	NA	NA
Scu-7	NA	NA	NA	NA	NA	NA	NA	NA	NA	NA	NA	NA	NA	NA	NA	NA	NA	NA	NA	NA	NA	NA	NA	NA	NA	NA	NA	NA	NA
Scu-8	NA	NA	NA	NA	NA	NA	NA	NA	NA	NA	NA	NA	NA	NA	NA	NA	NA	NA	NA	NA	NA	NA	NA	NA	NA	NA	NA	NA	NA
Scu-9	NA	NA	NA	NA	NA	NA	NA	NA	NA	NA	NA	NA	NA	NA	NA	NA	NA	NA	NA	NA	NA	NA	NA	NA	NA	NA	NA	NA	NA
Scu-10	NA	NA	NA	NA	NA	NA	NA	NA	NA	NA	NA	NA	NA	NA	NA	NA	NA	NA	NA	NA	NA	NA	NA	NA	NA	NA	NA	NA	NA

Scu-11	NA	NA	NA	NA	NA	NA	NA	NA	NA	NA	NA	NA	NA	NA	NA	NA	NA	NA	NA	NA	NA	NA	NA	NA	NA	NA	NA	NA	NA
Scu-12	NA	NA	NA	NA	NA	NA	NA	NA	NA	NA	NA	NA	NA	NA	NA	NA	NA	NA	NA	NA	NA	NA	NA	NA	NA	NA	NA	NA	NA
Scu-13	NA	NA	NA	NA	NA	NA	NA	NA	NA	NA	NA	NA	NA	NA	NA	NA	NA	NA	NA	NA	NA	NA	NA	NA	NA	NA	NA	NA	NA
Scu-14	NA	NA	NA	NA	NA	NA	NA	NA	NA	NA	NA	NA	NA	NA	NA	NA	NA	NA	NA	NA	NA	NA	NA	NA	NA	NA	NA	NA	NA
Scu-15	NA	NA	NA	NA	NA	NA	NA	NA	NA	NA	NA	NA	NA	NA	NA	NA	NA	NA	NA	NA	NA	NA	NA	NA	NA	NA	NA	NA	NA
Scu-16	NA	NA	NA	NA	NA	NA	NA	NA	NA	NA	NA	NA	NA	NA	NA	NA	NA	NA	NA	NA	NA	NA	NA	NA	NA	NA	NA	NA	NA
Scu-17	NA	NA	NA	NA	NA	NA	NA	NA	NA	NA	NA	NA	NA	NA	NA	NA	NA	NA	NA	NA	NA	NA	NA	NA	NA	NA	NA	NA	NA
Scu-18	NA	NA	NA	NA	NA	NA	NA	NA	NA	NA	NA	NA	NA	NA	NA	NA	NA	NA	NA	NA	NA	NA	NA	NA	NA	NA	NA	NA	NA
Scu-19	NA	NA	NA	NA	NA	NA	NA	NA	NA	NA	NA	NA	NA	NA	NA	NA	NA	NA	NA	NA	NA	NA	NA	NA	NA	NA	NA	NA	NA
Scu-20	NA	NA	NA	NA	NA	NA	NA	NA	NA	NA	NA	NA	NA	NA	NA	NA	NA	NA	NA	NA	NA	NA	NA	NA	NA	NA	NA	NA	NA
Scu-21	NA	NA	NA	NA	NA	NA	NA	NA	NA	NA	NA	NA	NA	NA	NA	NA	NA	NA	NA	NA	NA	NA	NA	NA	NA	NA	NA	NA	NA
Scu-22	NA	NA	NA	NA	NA	NA	NA	NA	NA	NA	NA	NA	NA	NA	NA	NA	NA	NA	NA	NA	NA	NA	NA	NA	NA	NA	NA	NA	NA
Scu-23	NA	NA	NA	NA	NA	NA	NA	NA	NA	NA	NA	NA	NA	NA	NA	NA	NA	NA	NA	NA	NA	NA	NA	NA	NA	NA	NA	NA	NA
Sch-1	NA	NA	NA	NA	NA	NA	NA	NA	NA	NA	NA	NA	NA	NA	NA	NA	NA	NA	NA	NA	NA	NA	NA	NA	NA	NA	NA	NA	NA
Sch-2	NA	NA	NA	NA	NA	NA	NA	NA	NA	NA	NA	NA	NA	NA	NA	NA	NA	NA	NA	NA	NA	NA	NA	NA	NA	NA	NA	NA	NA
Sch-3	NA	NA	NA	NA	NA	NA	NA	NA	NA	NA	NA	NA	NA	NA	NA	NA	NA	NA	NA	NA	NA	NA	NA	NA	NA	NA	NA	NA	NA
Sch-4	NA	NA	NA	NA	NA	NA	NA	NA	NA	NA	NA	NA	NA	NA	NA	NA	NA	NA	NA	NA	NA	NA	NA	NA	NA	NA	NA	NA	NA
Sch-5	NA	NA	NA	NA	NA	NA	NA	NA	NA	NA	NA	NA	NA	NA	NA	NA	NA	NA	NA	NA	NA	NA	NA	NA	NA	NA	NA	NA	NA
G11-1	NA	NA	NA	NA	NA	NA	AST[a] 33 ALT[a] 46	Elevated	NA	NA	NA	NA	NA	0.9	NA	NA	NA	NA	NA	NA	NA	NA	NA	NA	NA	NA	NA	NA	NA
G11-2	NA	NA	NA	NA	NA	NA	NA	NA	NA	NA	NA	NA	NA	NA	NA	NA	NA	NA	NA	NA	NA	NA	NA	NA	NA	NA	NA	NA	NA
G11-3	NA	NA	NA	NA	NA	NA	NA	NA	NA	NA	NA	NA	NA	NA	NA	NA	NA	NA	NA	NA	NA	NA	NA	NA	NA	NA	NA	NA	NA
G11-4	NA	NA	NA	NA	NA	NA	NA	NA	NA	NA	NA	NA	NA	NA	NA	NA	NA	NA	NA	NA	NA	NA	NA	NA	NA	NA	NA	NA	NA
G11-5	NA	NA	NA	NA	NA	NA	NA	NA	NA	NA	NA	NA	NA	NA	NA	NA	NA	NA	NA	NA	NA	NA	NA	NA	NA	NA	NA	NA	NA
G11-6	NA	NA	NA	NA	NA	NA	NA	NA	NA	NA	NA	NA	NA	NA	NA	NA	NA	NA	NA	NA	NA	NA	NA	NA	NA	NA	NA	NA	NA
G11-7	NA	NA	NA	NA	NA	NA	NA	NA	NA	NA	NA	NA	NA	NA	NA	NA	NA	NA	NA	NA	NA	NA	NA	NA	NA	NA	NA	NA	NA
G11-8	NA	NA	NA	NA	NA	NA	NA	NA	NA	NA	NA	NA	NA	NA	NA	NA	NA	NA	NA	NA	NA	NA	NA	NA	NA	NA	NA	NA	NA
G11-9	NA	NA	NA	NA	NA	NA	NA	NA	NA	NA	NA	NA	NA	NA	NA	NA	NA	NA	NA	NA	NA	NA	NA	NA	NA	NA	NA	NA	NA

G11-10	NA	NA	NA	NA	NA	NA	NA	NA	NA	NA	NA	NA	NA	NA	NA	NA	NA	NA	NA	NA	NA	NA	NA	NA	NA	NA	NA	NA	NA
G11-11	NA	NA	NA	NA	NA	NA	NA	NA	NA	NA	NA	NA	NA	NA	NA	NA	NA	NA	NA	NA	NA	NA	NA	NA	NA	NA	NA	NA	NA
Ba1	NA	NA	NA	NA	NA	NA	NA	NA	NA	NA	NA	NA	NA	NA	NA	NA	NA	NA	NA	NA	NA	NA	NA	NA	NA	NA	NA	NA	NA
Bl-1	NA	NA	9500	860	630	10	NA	NA	NA	138	3.1	NA	NA	2	NA	NA	NA	NA	Normal	0.77	NA	NA	NA	NA	NA	NA	NA	NA	NA
Meh-1	NA	NA	NA	NA	NA	NA	NA	NA	NA	NA	NA	NA	NA	NA	9	Normal	NA	NA	NA	0.8	NA	NA	NA	NA	NA	NA	NA	NA	NA
Meh-2	NA	NA	NA	NA	NA	NA	NA	NA	NA	NA	NA	NA	192	NA	9	Normal	NA	NA	NA	0.66	NA	NA	Negative	NA	NA	NA	NA	NA	NA
Cas-1	NA	NA	NA	NA	NA	NA	NA	NA	NA	NA	NA	NA	NA	NA	NA	XIII 35% [f]	0.9	NA	NA	NA	NA	NA	NA	NA	NA	NA	NA	NA	NA
D-1	NA	NA	NA	NA	NA	NA	NA	NA	NA	NA	NA	NA	NA	NA	NA	Neg	NA	NA	NA	NA	NA	NA	NA	NA	NA	NA	NA	NA	NA
E-1	NA	NA	NA	NA	NA	NA	NA	NA	NA	NA	NA	NA	NA	NA	NA	NA	NA	NA	NA	NA	NA	NA	NA	NA	NA	NA	NA	NA	NA
E-2	NA	NA	NA	NA	NA	NA	NA	NA	NA	NA	NA	NA	NA	NA	NA	NA	NA	NA	NA	NA	NA	NA	NA	NA	NA	NA	NA	NA	NA
F-1	NA	5.18	NA	NA	NA	NA	ALT 16	NA	NA	NA	NA	NA	337	NA	0.57	II: 71, IX: >150, V: 71, VII: 68, VIII: 114, X: 69, XI: 103, XII:99, XIII: 35	0.9	246	NA	NA	NA	NA	NA	NA	NA	NA	NA	NA	NA
Gar-1	NA	NA	NA	NA	NA	NA	NA	NA	NA	NA	NA	NA	NA	NA	NA	NA	NA	NA	NA	NA	NA	NA	NA	NA	NA	NA	NA	NA	NA
Ge-1	Normal	Normal	NA	NA	NA	NA	NA	NA	NA	NA	NA	NA	NA	NA	Normal	NA	NA	NA	NA	NA	NA	NA	ruled out	NA	NA	NA	NA	NA	NA
Ge-2	Normal	Normal	NA	NA	NA	NA	NA	NA	NA	NA	NA	NA	NA	NA	Normal	NA	NA	NA	NA	NA	NA	NA	ruled out	NA	NA	NA	NA	NA	NA
GC-1	NA	NA	NA	NA	NA	NA	NA	NA	NA	NA	NA	NA	NA	NA	NA	NA	NA	NA	NA	NA	NA	NA	NA	NA	NA	NA	NA	NA	NA
GC-2	NA	NA	NA	NA	NA	NA	NA	NA	NA	NA	NA	NA	NA	NA	NA	NA	NA	NA	NA	NA	NA	NA	NA	NA	NA	NA	NA	NA	NA
GC-3	NA	NA	NA	NA	NA	NA	NA	NA	NA	NA	NA	NA	NA	NA	NA	NA	NA	NA	NA	NA	NA	NA	NA	NA	NA	NA	NA	NA	NA
GC-4	NA	NA	NA	NA	NA	NA	NA	NA	NA	NA	NA	NA	NA	NA	NA	NA	NA	NA	NA	NA	NA	NA	NA	NA	NA	NA	NA	NA	NA
GC-5	NA	NA	NA	NA	NA	NA	NA	NA	NA	NA	NA	NA	NA	NA	NA	NA	NA	NA	NA	NA	NA	NA	NA	NA	NA	NA	NA	NA	NA
GC-6	NA	NA	NA	NA	NA	NA	NA	NA	NA	NA	NA	NA	NA	NA	NA	NA	NA	NA	NA	NA	NA	NA	NA	NA	NA	NA	NA	NA	NA

GC-7	NA	NA	NA	NA	NA	NA	NA	NA	NA	NA	NA	NA	NA	NA	NA	NA	NA	NA	NA	NA	NA	NA	NA	NA	NA	NA	NA	NA	NA
GC-8	NA	NA	NA	NA	NA	NA	NA	NA	NA	NA	NA	NA	NA	NA	NA	NA	NA	NA	NA	NA	NA	NA	NA	NA	NA	NA	NA	NA	NA
GC-9	NA	NA	NA	NA	NA	NA	NA	NA	NA	NA	NA	NA	NA	NA	NA	NA	NA	NA	NA	NA	NA	NA	NA	NA	NA	NA	NA	NA	NA
GC-10	NA	NA	NA	NA	NA	NA	NA	NA	NA	NA	NA	NA	NA	NA	NA	NA	NA	NA	NA	NA	NA	NA	NA	NA	NA	NA	NA	NA	NA
GC-11	NA	NA	NA	NA	NA	NA	NA	NA	NA	NA	NA	NA	NA	NA	NA	NA	NA	NA	NA	NA	NA	NA	NA	NA	NA	NA	NA	NA	NA
GC-12	NA	NA	NA	NA	NA	NA	NA	NA	NA	NA	NA	NA	NA	NA	NA	NA	NA	NA	NA	NA	NA	NA	NA	NA	NA	NA	NA	NA	NA
GC-13	NA	NA	NA	NA	NA	NA	NA	NA	NA	NA	NA	NA	NA	NA	NA	NA	NA	NA	NA	NA	NA	NA	NA	NA	NA	NA	NA	NA	NA
GC-14	NA	NA	NA	NA	NA	NA	NA	NA	NA	NA	NA	NA	NA	NA	NA	NA	NA	NA	NA	NA	NA	NA	NA	NA	NA	NA	NA	NA	NA
GC-15	NA	NA	NA	NA	NA	NA	NA	NA	NA	NA	NA	NA	NA	NA	NA	NA	NA	NA	NA	NA	NA	NA	NA	NA	NA	NA	NA	NA	NA
GC-16	NA	NA	NA	NA	NA	NA	NA	NA	NA	NA	NA	NA	NA	NA	NA	NA	NA	NA	NA	NA	NA	NA	NA	NA	NA	NA	NA	NA	NA
GC-17	NA	NA	NA	NA	NA	NA	NA	NA	NA	NA	NA	NA	NA	NA	NA	NA	NA	NA	NA	NA	NA	NA	NA	NA	NA	NA	NA	NA	NA
GC-18	NA	NA	NA	NA	NA	NA	NA	NA	NA	NA	NA	NA	NA	NA	NA	NA	NA	NA	NA	NA	NA	NA	NA	NA	NA	NA	NA	NA	NA
GC-19	NA	NA	NA	NA	NA	NA	NA	NA	NA	NA	NA	NA	NA	NA	NA	NA	NA	NA	NA	NA	NA	NA	NA	NA	NA	NA	NA	NA	NA
GC-20	NA	NA	NA	NA	NA	NA	NA	NA	NA	NA	NA	NA	NA	NA	NA	NA	NA	NA	NA	NA	NA	NA	NA	NA	NA	NA	NA	NA	NA
GC-21	NA	NA	NA	NA	NA	NA	NA	NA	NA	NA	NA	NA	NA	NA	NA	NA	NA	NA	NA	NA	NA	NA	NA	NA	NA	NA	NA	NA	NA
GC-22	NA	NA	NA	NA	NA	NA	NA	NA	NA	NA	NA	NA	NA	NA	NA	NA	NA	NA	NA	NA	NA	NA	NA	NA	NA	NA	NA	NA	NA
GC-23	NA	NA	NA	NA	NA	NA	NA	NA	NA	NA	NA	NA	NA	NA	NA	NA	NA	NA	NA	NA	NA	NA	NA	NA	NA	NA	NA	NA	NA
GC-24	NA	NA	NA	NA	NA	NA	NA	NA	NA	NA	NA	NA	NA	NA	NA	NA	NA	NA	NA	NA	NA	NA	NA	NA	NA	NA	NA	NA	NA
GC-25	NA	NA	NA	NA	NA	NA	NA	NA	NA	NA	NA	NA	NA	NA	NA	NA	NA	NA	NA	NA	NA	NA	NA	NA	NA	NA	NA	NA	NA
GC-26	NA	NA	NA	NA	NA	NA	NA	NA	NA	NA	NA	NA	NA	NA	NA	NA	NA	NA	NA	NA	NA	NA	NA	NA	NA	NA	NA	NA	NA
GC-27	NA	NA	NA	NA	NA	NA	NA	NA	NA	NA	NA	NA	NA	NA	NA	NA	NA	NA	NA	NA	NA	NA	NA	NA	NA	NA	NA	NA	NA
J-1	NA	NA	NA	NA	NA	NA	NA	NA	NA	NA	NA	NA	NA	NA	NA	NA	NA	NA	NA	NA	NA	NA	NA	NA	NA	NA	NA	NA	NA
Ram-1	NA	NA	NA	NA	NA	NA	NA	NA	NA	NA	NA	NA	NA	NA	NA	NA	NA	NA	NA	NA	NA	NA	NA	NA	NA	NA	NA	NA	NA
W-1	NA	NA	NA	NA	NA	NA	NA	NA	NA	NA	NA	NA	NA	NA	NA	NA	NA	NA	NA	NA	NA	NA	NA	NA	NA	NA	NA	NA	NA
W-2	NA	NA	NA	NA	NA	NA	NA	NA	NA	NA	NA	NA	NA	NA	NA	NA	NA	NA	NA	NA	NA	NA	NA	NA	NA	NA	NA	NA	NA
W-3	NA	NA	NA	NA	NA	NA	NA	NA	NA	NA	NA	NA	NA	NA	NA	NA	NA	NA	NA	NA	NA	NA	NA	NA	NA	NA	NA	NA	NA
X-1	NA	NA	NA	NA	NA	NA	NA	NA	NA	NA	NA	NA	NA	NA	NA	NA	NA	NA	NA	NA	NA	NA	NA	NA	NA	NA	NA	NA	NA

Mu-1	NA	NA	NA	NA	NA	NA	AST normal	NA	NA	NA	NA	NA	NA	NA	NA	Normal	NA	NA	NA	Normal	NA	NA	NA	NA	NA	NA	NA	NA	NA
Al-1	NA	NA	Increased WBC at 18.7 £10"9/L (mainly neutrophils)	NA	NA	NA	Slightly elevated ALT	Slightly elevated	NA	NA	NA	NA	Slightly elevated	NA	NA	NA	NA	NA	NA	high creatinine at 254 umol/L	NA	NA	NA	Negative	NA	NA	NA	NA	NA
Alt-1	NA	NA	NA	NA	NA	NA	NA	NA	NA	NA	NA	NA	NA	NA	NA	NA	NA	NA	NA	NA	NA	NA	NA	NA	NA	NA	NA	NA	NA
Alt-2	NA	NA	NA	NA	NA	NA	NA	NA	NA	NA	NA	NA	NA	NA	NA	NA	NA	NA	NA	Normal 0.5 mg/dL	NA	NA	NA	NA	NA	NA	NA	NA	NA
Alt-3	NA	NA	NA	NA	NA	NA	NA	NA	NA	NA	NA	NA	NA	NA	NA	NA	NA	NA	NA	Normal 0.8 mg/dL	NA	NA	NA	NA	NA	NA	NA	NA	NA
Alt-4	NA	NA	NA	NA	NA	NA	NA	NA	NA	NA	NA	NA	NA	NA	NA	NA	NA	NA	NA	NA	NA	NA	NA	NA	NA	NA	NA	NA	NA
Alt-5	NA	NA	NA	NA	NA	NA	NA	NA	NA	NA	NA	NA	NA	NA	NA	NA	NA	NA	NA	NA	NA	NA	NA	NA	NA	NA	NA	NA	NA
Alt-6	NA	NA	NA	NA	NA	NA	NA	NA	NA	NA	NA	NA	NA	NA	NA	NA	NA	NA	NA	NA	NA	NA	NA	NA	NA	NA	NA	NA	NA
Alt-7	NA	NA	NA	NA	NA	NA	NA	NA	NA	NA	NA	NA	NA	NA	NA	NA	NA	NA	NA	NA	NA	NA	NA	NA	NA	NA	NA	NA	NA
Alt-8	NA	NA	NA	NA	NA	NA	NA	NA	NA	NA	NA	NA	NA	NA	NA	NA	NA	NA	NA	NA	NA	NA	NA	NA	NA	NA	NA	NA	NA
Ban-1	NA	Normal	Normal	Normal	Normal	Normal	NA	NA	NA	NA	NA	NA	NA	NA	NA	NA	NA	NA	NA	NA	NA	NA	NA	NA	NA	NA	NA	NA	NA
Ban-2	NA	NA	NA	NA	NA	NA	NA	NA	NA	NA	NA	NA	NA	NA	NA	NA	NA	NA	NA	NA	NA	NA	NA	NA	NA	NA	NA	NA	NA
Ban-3	NA	NA	NA	NA	NA	NA	NA	NA	NA	NA	NA	NA	NA	NA	NA	NA	NA	NA	NA	NA	NA	NA	NA	NA	NA	NA	NA	NA	NA
Bon-1	NA	NA	NA	NA	NA	NA	NA	NA	NA	NA	NA	NA	NA	NA	NA	NA	NA	NA	NA	NA	NA	NA	NA	NA	NA	NA	NA	NA	NA
Bour-1	NA	NA	NA	NA	NA	NA	NA	NA	NA	NA	NA	NA	NA	NA	NA	NA	NA	NA	NA	NA	NA	NA	NA	NA	NA	NA	NA	NA	NA
Bour-2	NA	NA	NA	NA	NA	NA	NA	NA	NA	NA	NA	NA	NA	NA	NA	NA	NA	NA	NA	NA	NA	NA	NA	NA	NA	NA	NA	NA	NA
Bour-3	NA	NA	NA	NA	NA	NA	NA	NA	NA	NA	NA	NA	NA	NA	NA	NA	NA	NA	NA	NA	NA	NA	NA	NA	NA	NA	NA	NA	NA
Cho-1	NA	NA	WBC	NA	NA	NA	Aspartate	NA	85 U/L	NA	NA	12.5	203	NA	0.91 mg/dL	NA	NA	NA	NA	0.97 mg/dL	7.3	4.3	NA	NA	NA	NA	NA	NA	NA

			7.40 per mm³<br>Ref: 4-10				14 U/L<br>REF 0-40<br>REF Alanin 15 U/L<br>0-40		REF 40-129			Mg/dL<br>Ref: 6-20	U/L<br>REF 135-225		REF 0-1.4					ref<br>0.7–1.2	g/dL ref<br>6.6–8.7	g/dL<br>ref 3.4-4.8							
Cp-1	NA	NA	mildly increased WBC	NA	NA	NA	NA	NA	NA	NA	NA	NA	NA	NA	NA	NA	NA	NA	NA	NA	NA	NA	NA	NA	YES	NA	NA	NA	NA
Cp-2	NA	NA	NA	NA	NA	NA	NA	NA	NA	NA	NA	NA	NA	NA	NA	NA	NA	NA	NA	NA	NA	NA	NA	NA	NA	NA	NA	NA	NA
Cp-3	NA	NA	NA	NA	NA	NA	NA	NA	NA	NA	NA	NA	NA	NA	NA	NA	NA	NA	NA	NA	NA	NA	NA	NA	NA	NA	NA	NA	NA
Gan-1	NA	NA	NA	NA	NA	NA	NA	NA	NA	NA	NA	NA	NA	NA	NA	NA	NA	NA	NA	NA	NA	NA	NA	NA	NA	NA	NA	NA	NA
Gan-2	NA	NA	NA	NA	NA	NA	NA	NA	NA	NA	NA	NA	NA	NA	NA	NA	NA	NA	NA	NA	NA	NA	NA	NA	NA	NA	NA	NA	NA
Gan-3	NA	NA	NA	NA	NA	NA	NA	NA	NA	NA	NA	NA	NA	NA	NA	NA	NA	NA	NA	NA	NA	NA	NA	NA	NA	NA	NA	NA	NA
Gan-4	NA	NA	NA	NA	NA	NA	NA	NA	NA	NA	NA	NA	NA	NA	NA	NA	NA	NA	NA	NA	NA	NA	NA	NA	NA	NA	NA	NA	NA
Gan-5	NA	NA	NA	NA	NA	NA	NA	NA	NA	NA	NA	NA	NA	NA	NA	NA	NA	NA	NA	NA	NA	NA	NA	NA	NA	NA	NA	NA	NA
Gan-6	NA	NA	NA	NA	NA	NA	NA	NA	NA	NA	NA	NA	NA	NA	NA	NA	NA	NA	NA	NA	NA	NA	NA	NA	NA	NA	NA	NA	NA
Gatt-1	NA	NA	NA	NA	NA	NA	NA	NA	NA	NA	NA	NA	NA	NA	NA	Coagulation factor 13<br>119<br>>70%	NA	NA	NA	NA	NA	NA	NA	NA	NA	83<br>(9–140 ng/mL)	12 (0–7.0 pg/mL)	NA	NA
Gatt-2	NA	NA	NA	NA	NA	NA	NA	NA	NA	NA	NA	NA	NA	NA	NA	Coagulation factor 13<br>25<br>Day 2<br>>70%	NA	NA	NA	NA	NA	NA	NA	NA	NA	80<br>(9–140 ng/mL)	11<br>(0–7.0 pg/mL)	NA	NA
Gess-1	NA	NA	NA	NA	NA	NA	NA	NA	NA	NA	NA	NA	NA	NA	NA	NA	NA	NA	NA	NA	NA	NA	NA	NA	NA	NA	NA	NA	NA

Gess-2	NA	NA	NA	NA	NA	NA	NA	NA	NA	NA	NA	NA	NA	NA	NA	NA	NA	NA	NA	NA	NA	NA	NA	NA	NA	NA	NA	NA	NA
Gra-1	NA	NA	NA	NA	NA	NA	NA	NA	NA	NA	NA	NA	NA	Normal	NA	NA	NA	NA	NA	NA	NA	NA	NA	NA	NA	NA	NA	NA	NA
Gua-1	NA	NA	NA	NA	NA	NA	NA	NA	NA	NA	NA	NA	NA	NA	NA	NA	NA	NA	NA	NA	NA	NA	NA	NA	NA	NA	NA	NA	NA
H-1	NA	NA	NA	NA	NA	NA	NA	NA	NA	NA	NA	NA	NA	NA	NA	NA	NA	NA	NA	NA	NA	NA	NA	NA	NA	NA	NA	NA	NA
Ik-1	NA	NA	NA	NA	NA	NA	NA	NA	NA	NA	NA	NA	NA	NA	NA	NA	NA	NA	NA	NA	NA	NA	NA	NA	NA	NA	NA	NA	NA
Ja-1	NA	NA	NA	NA	NA	NA	NA	NA	NA	NA	NA	NA	NA	NA	hyperbilirubinemia of 50 mmol/L.	NA	NA	NA	NA	NA	NA	NA	NA	NA	NA	NA	NA	4.9<br>REF: 1-4<br>HDL cholesterol<br>1.11 mmol/L<br>Ref 0.91–2.21 mmol/L	NA
Kh-1	NA	NA	3.66 x 109 /L<br>neutrophil 75%	lymphocyte 18%	monocyte 4.7 %	eosinophil 2% basophil 0.3 %)	NA	NA	NA	NA	NA	NA	NA	NA	NA	NA	NA	NA	NA	NA	NA	NA	NA	NA	NA	NA	NA	NA	NA
Ma-1	NA	NA	NA	NA	NA	NA	NA	NA	NA	NA	NA	NA	NA	NA	NA	NA	NA	NA	NA	NA	NA	NA	NA	NA	NA	NA	NA	NA	NA
Mi-1	NA	NA	NA	NA	NA	NA	NA	NA	NA	NA	NA	NA	NA	NA	NA	NA	NA	NA	NA	NA	NA	NA	NA	NA	NA	NA	NA	NA	NA
Mi-2	NA	NA	NA	NA	NA	NA	NA	NA	NA	NA	NA	NA	NA	NA	NA	NA	NA	NA	NA	NA	NA	NA	NA	NA	NA	NA	NA	NA	NA
Ps-1	NA	NA	NA	NA	NA	NA	NA	NA	NA	NA	NA	NA	NA	NA	NA	NA	NA	NA	NA	NA	NA	NA	NA	NA	NA	NA	NA	NA	NA
Pat-1	NA	NA	NA	NA	NA	NA	NA	NA	NA	NA	NA	NA	NA	NA	NA	NA	NA	NA	NA	NA	NA	NA	NA	NA	NA	NA	NA	NA	NA
Pat-2	NA	NA	NA	NA	NA	NA	NA	NA	NA	NA	NA	NA	NA	NA	NA	NA	NA	NA	NA	NA	NA	NA	NA	NA	NA	NA	NA	NA	NA
Pat-3	NA	NA	NA	NA	NA	NA	NA	NA	NA	NA	NA	NA	NA	NA	NA	NA	NA	NA	NA	NA	NA	NA	NA	NA	NA	NA	NA	NA	NA
Pal-1	NA	anemia with	NA	NA	NA	NA	NA	NA	NA	NA	NA	NA	Increased	NA	Increased	NA	NA	NA	NA	Increased	NA	NA	NA	NA	NA	NA	NA	NA	NA

		increased number of reticulocyte																											
R-1	NA	NA	Normal	Normal	Normal	Normal	Normal	Normal	Normal	NA	NA	NA	Normal	NA	NA	NA	NA	NA	NA	NA	NA	NA	NA	NA	Negative	NA	NA	NA	NA
Sol-1	NA	NA	NA	NA	NA	NA	NA	NA	NA	NA	NA	NA	NA	NA	NA	NA	NA	NA	NA	NA	NA	NA	NA	NA	NA	NA	NA	NA	NA
Sol-2	NA	NA	NA	NA	NA	NA	alanine amino transferase (144 IU/L)<br><br>DERANGED	NA	NA	NA	NA	NA	NA	NA	raised bilirubin (26 mmol/L)<br><br>DERANGED	NA	NA	NA	NA	NA	NA	NA	NA	NA	NA	NA	NA	NA	NA
Sol-3	NA	NA	NA	NA	NA	NA	NA	NA	NA	NA	NA	NA	NA	NA	NA	NA	NA	NA	NA	NA	NA	NA	NA	NA	NA	NA	NA	NA	Normal
Sør-1	NA	NA	*10^9/L<br><br>Ref: 1.6-5.9<br><br>6.25	*10^9/L<br><br>Ref: 1-3.5<br><br>3.42	*10^9/L<br><br>Ref: 0.2-0.8<br><br>0.98	*10^9/L<br><br>Ref: 0.01-0.5<br><br>0.27<br><br>Basophil *10^9/L<br><br>Ref: 0.01 0.1<br><br>0.05	marginally increased alanine amino transferase<br><br>ALT, U/L (ref., 10-45)<br><br>29-134	NA	NA	NA	NA	NA	NA	NA	NA	consumption coagulopathy<br><br>Clotting factors II, VII, X (ref>. 0.60)<br><br>0.91	NA	NA	NA	NA	NA	NA	NA	NA	NA	NA	NA	NA	NA

Sur-1	NA	NA	NA	NA	NA	NA	NA	NA	ALP (U/L)<br>87	Sodium (mmol/L)<br>140	Potassium (mmol/L)<br>3.6	NA	NA	NA	Bilirubin (µmol/L)<br>7	NA	NA	NA	NA	Creatinine (µmol/L)<br>65	NA	NA	NA	NA	NA	NA	NA	NA	Urea (mmol/L)<br>5<br>Normal
Tej-1	NA	NA	NA	NA	NA	NA	NA	NA	NA	NA	NA	NA	NA	NA	NA	NA	NA	NA	NA	NA	NA	NA	NA	NA	NA	NA	NA	NA	NA
Ti-1	Haptoglobin (g/l)<br>0.3-2<br><0.1	NA	NA	NA	NA	NA	NA	NA	NA	NA	NA	NA	LDH (U/l) < 247<br>1083	NA	NA	NA	NA	NA	NA	45-84 (µmol/l)<br>42 day 1<br>189 day 15	NA	NA	NA	NA	NA	NA	NA	NA	NA
Ti-2	NA	NA	NA	NA	NA	NA	NA	NA	NA	NA	NA	NA	LDH (U/l) < 247<br>247	NA	NA	NA	NA	NA	NA	45-84 (µmol/l)<br>69 day 1<br>71 day 15	NA	NA	NA	NA	NA	NA	NA	mild hypercholesterinemia	NA
Ti-3	NA	NA	NA	NA	NA	NA	NA	NA	NA	NA	NA	NA	LDH (U/l) < 247<br>198	NA	NA	NA	NA	NA	NA	45-84 (µmol/l)<br>69 day 1	NA	NA	NA	NA	NA	NA	NA	NA	NA
Ti-4	NA	NA	NA	NA	NA	NA	NA	NA	NA	NA	NA	NA	LDH	NA	NA	NA	NA	NA	NA	NA	NA	NA	NA	NA	NA	NA	NA	NA	NA

													(U/ l) < 24 7 35 3																
Ti-5	NA	NA	NA	NA	NA	NA	NA	NA	N A	NA	NA	NA	LD H (U/ l) < 24 7 36 0 DA Y 3	NA	NA	NA	NA	NA	NA	NA	NA	NA	NA	NA	NA	NA	NA	NA	NA
Tu-1	NA	NA	NA	NA	NA	NA	aspU/ L 5-34 30 At day 6 Ala U/L 0-55 25 at day 6	NA	N A	NA	NA	NA	U/ L 12 5- 22 0 22 7 at day 6	NA	NA	NA	NA	NA	NA	NA	NA	NA	NA	NA	NA	NA	NA	NA	NA
Var-1	NA	NA	NA	NA	NA	NA	NA	NA	N A	NA	NA	NA	NA	NA	NA	NA	NA	NA	NA	NA	NA	NA	NA	NA	NA	NA	NA	NA	NA
Var-1	NA	NA	NA	NA	NA	NA	NA	NA	N A	NA	NA	NA	NA	NA	NA	NA	NA	NA	NA	NA	NA	NA	NA	NA	NA	NA	NA	NA	NA
Var-2	NA	NA	NA	NA	NA	NA	NA	NA	N A	NA	NA	NA	NA	NA	NA	NA	NA	NA	NA	NA	NA	NA	NA	NA	NA	NA	NA	NA	NA
Var-3	NA	NA	NA	NA	NA	NA	NA	NA	N A	NA	NA	NA	NA	NA	NA	NA	NA	NA	NA	NA	NA	NA	NA	NA	NA	NA	NA	NA	NA
Var-4	NA	NA	NA	NA	NA	NA	NA	NA	N A	NA	NA	NA	NA	NA	NA	NA	NA	NA	NA	NA	NA	NA	NA	NA	NA	NA	NA	NA	NA
Var-5	NA	NA	NA	NA	NA	NA	NA	NA	N A	NA	NA	NA	NA	NA	NA	NA	NA	NA	NA	NA	NA	NA	NA	NA	NA	NA	NA	NA	NA
Var-6	NA	NA	NA	NA	NA	NA	NA	NA	N A	NA	NA	NA	NA	NA	NA	NA	NA	NA	NA	NA	NA	NA	NA	NA	NA	NA	NA	NA	NA
Var-7	NA	NA	NA	NA	NA	NA	NA	NA	N A	NA	NA	NA	NA	NA	NA	NA	NA	NA	NA	NA	NA	NA	NA	NA	NA	NA	NA	NA	NA
Var-8	NA	NA	NA	NA	NA	NA	NA	NA	N A	NA	NA	NA	NA	NA	NA	NA	NA	NA	NA	NA	NA	NA	NA	NA	NA	NA	NA	NA	NA
Var-9	NA	NA	NA	NA	NA	NA	NA	NA	N A	NA	NA	NA	NA	NA	NA	NA	NA	NA	NA	NA	NA	NA	NA	NA	NA	NA	NA	NA	NA

Wan-1	NA	NA	NA	NA	NA	NA	NA	NA	NA	NA	NA	NA	NA	NA	NA	NA	NA	NA	NA	NA	NA	NA	NA	NA	NA	NA	NA	NA	NA
Wi-1	NA	NA	NA	NA	NA	NA	NA	NA	NA	NA	NA	NA	NA	NA	NA	NA	NA	NA	NA	NA	NA	NA	NA	NA	NA	NA	NA	NA	NA
Z-1	NA	NA	NA	NA	NA	NA	NA	NA	NA	NA	NA	NA	NA	NA	NA	NA	NA	NA	NA	NA	NA	NA	NA	NA	NA	NA	NA	NA	NA

**Gamma glutamyl transferase, °°°Alaklin phosphatas, °° Nitrogen Urea, ° Lactate Dehydrogenase, ±± Creatinine (mg/dl)[Ξ], ± Total protein, □□□ Paroxysmal nocturnal haemoglobinuria, □□ Septic screen of blood, urine, and respiratory cultures, □ Acute kidney injury, ᵽ Ref: 50–210 mg/dL (Blauenfeldt-2021), t Ref: 4.50–5.90 *10^12/L (Franchini-2021), # Ref: 2000–7000 per mcL (Blauenfeldt-2021), § Ref: 1300–3500 per mcL (Blauenfeldt-2021), ¶ Ref: 200–700 per mcL (Blauenfeldt-2021), æ Ref: < 500 per mcL (Blauenfeldt-2021), Ƀ Ref: < 35 U/liter (Greinacher—2021), 5-45 IU/L (Franchini—2021), ∮ Ref: < 40 (Greinacher—2021), Ǽ Ref: 137–145 mmol/L (Blauenfeldt-2021), Ꮟ Ref: 3.5–4.6 mmol/L (Blauenfeldt-2021), 𝕬 Ref: <250 U/liter (Greinacher—2021), <240 U/L (Mehta-2021), 150–450 (Franchini—2021), 𝕭 Ref: < 1.6 mmol/liter (Greinacher—2021), 4.5–22.5 mg/dl (equal to 0.5 to 2.5 mmol/liter) (Blauenfeldt-2021), 𝕯 Ref: < 0.5 mcg/L (Blauenfeldt-2021), Ξ Ref: 0.51–1.02 mg/dl (Blauenfeldt-2021), 45–120 µmol/L (Mehta-2021), a glutamate pyruvate transaminase (GPT); Alanine Aminotransferase (ALT/GPT), Aspartate Aminotransferase (AST/GOT), n; Ref: 3–20 µmol/L (Mehta-2021). 0.20-1.00 (Franchini—2021), f Ref: 70–150% (Castelli-2021), K Ref: II, IX, V, VII, VIII, X: 50–150 %, XI, XII: 70-120 %, XIII: 70-150% (Franchini—2021), q Ref: 3.9–26.8 ng/ml (Castelli-2021, Franchini—2021), g Ref: 197–771 pg/ml (Franchini—2021)

Table S4. Details of thrombophilia marker assays in case reports on VITT after ChAdOx1 nCov-19 vaccination.

Patient code	EXTEM CT[α]	EXTEM A10 [β]	FIBTEM A10 [χ]	INTEM CT[δ]	Fibrin degradation product	Protein C activity ♦	Protein S, free antigen♣	VWF [L]	Thrombophilia mutations (Factor V Leiden, Prothrombin G20210A, MTHFR, JAK2)	Antiphospholipid antibodies♠	A, anti-thyroid peroxidase, Myeloperoxidase anti-neutrophil cytoplasmic anti-bodies (ANCA), rheumatoid factor, and antinuclear antibodies, extractable nuclear antigen, dsDNA, anti-globulin test	Neutrophil DNA extracellular traps (NETs)	Schistocytes (%)[o]	Blood film	Immunoglobulins	Complement	Cryoglobulins	ADAMTS13[ƒ]	Antiplatelet antibodies[⊥]	Homocysteine (μmol/L)[w]
G9-1	NA	NA	NA	NA	NA	NA	NA	+++	NA	++++	NA	NA	NA	NA	NA	NA	NA	NA	NA	NA
G9-2	NA	NA	NA	NA	NA	NA	NA	+++	NA	++++	NA	NA	NA	NA	NA	NA	NA	NA	NA	NA
G9-3	NA	NA	NA	NA	NA	NA	NA	+++	NA	++++	NA	NA	NA	NA	NA	NA	NA	NA	NA	NA
G9-4	NA	NA	NA	NA	NA	NA	NA	+++	NA	++++	NA	NA	NA	NA	NA	NA	NA	NA	NA	NA
G9-5	NA	NA	NA	NA	NA	NA	NA	+++	NA	++++	NA	NA	NA	NA	NA	NA	NA	NA	NA	NA
G9-6	NA	NA	NA	NA	NA	NA	NA	+++	NA	++++	NA	NA	NA	NA	NA	NA	NA	NA	NA	NA
G9-7	NA	NA	NA	NA	NA	NA	NA	+++	NA	++++	NA	NA	NA	NA	NA	NA	NA	NA	NA	NA
G9-8	NA	NA	NA	NA	NA	NA	NA	+++	NA	++++	NA	NA	NA	NA	NA	NA	NA	NA	NA	NA
G9-9	NA	NA	NA	NA	NA	NA	NA	+++	NA	++++	NA	NA	NA	NA	NA	NA	NA	NA	NA	NA
A1	NA	NA	NA	NA	NA	NA	NA	NA	NA	NA	NA	NA	NA	NA	NA	NA	NA	NA	NA	NA
A2	NA	NA	NA	NA	NA	NA	NA	NA	NA	NA	NA	NA	NA	NA	NA	NA	NA	NA	NA	NA
A3	NA	NA	NA	NA	NA	NA	NA	NA	NA	NA	NA	NA	NA	NA	NA	NA	NA	NA	NA	NA
Scu-1	NA	NA	NA	NA	NA	NA	NA	NA	^^	***	Negative	NA	NA	NA	NA	NA	NA	NA	NA	NA
Scu-2	NA	NA	NA	NA	NA	NA	NA	NA	^^	***	Negative	NA	NA	NA	NA	NA	NA	NA	NA	NA
Scu-3	NA	NA	NA	NA	NA	NA	NA	NA	^^	***	Negative	NA	NA	NA	NA	NA	NA	NA	NA	NA
Scu-4	NA	NA	NA	NA	NA	NA	NA	NA	^^	***	Negative	NA	NA	NA	NA	NA	NA	NA	NA	NA
Scu-5	NA	NA	NA	NA	NA	NA	NA	NA	^^	***	Negative	NA	NA	NA	NA	NA	NA	NA	NA	NA
Scu-6	NA	NA	NA	NA	NA	NA	NA	NA	^^	***	Negative	NA	NA	NA	NA	NA	NA	NA	NA	NA
Scu-7	NA	NA	NA	NA	NA	NA	NA	NA	^^	***	Negative	NA	NA	NA	NA	NA	NA	NA	NA	NA
Scu-8	NA	NA	NA	NA	NA	NA	NA	NA	^^	***	Negative	NA	NA	NA	NA	NA	NA	NA	NA	NA

Scu-9	NA	NA	NA	NA	NA	NA	NA	NA	^^	***	Negative	NA	NA	NA	NA	NA	NA	NA	NA	NA
Scu-10	NA	NA	NA	NA	NA	NA	NA	NA	^^	***	Negative	NA	NA	NA	NA	NA	NA	NA	NA	NA
Scu-11	NA	NA	NA	NA	NA	NA	NA	NA	^^	***	Negative	NA	NA	NA	NA	NA	NA	NA	NA	NA
Scu-12	NA	NA	NA	NA	NA	NA	NA	NA	^^	***	Negative	NA	NA	NA	NA	NA	NA	NA	NA	NA
Scu-13	NA	NA	NA	NA	NA	NA	NA	NA	^^	***	Negative	NA	NA	NA	NA	NA	NA	NA	NA	NA
Scu-14	NA	NA	NA	NA	NA	NA	NA	NA	^^	***	Negative	NA	NA	NA	NA	NA	NA	NA	NA	NA
Scu-15	NA	NA	NA	NA	NA	NA	NA	NA	^^	***	Negative	NA	NA	NA	NA	NA	NA	NA	NA	NA
Scu-16	NA	NA	NA	NA	NA	NA	NA	NA	^^	***	Negative	NA	NA	NA	NA	NA	NA	NA	NA	NA
Scu-17	NA	NA	NA	NA	NA	NA	NA	NA	^^	***	Negative	NA	NA	NA	NA	NA	NA	NA	NA	NA
Scu-18	NA	NA	NA	NA	NA	NA	NA	NA	^^	***	Negative	NA	NA	NA	NA	NA	NA	NA	NA	NA
Scu-19	NA	NA	NA	NA	NA	NA	NA	NA	^^	***	Negative	NA	NA	NA	NA	NA	NA	NA	NA	NA
Scu-20	NA	NA	NA	NA	NA	NA	NA	NA	^^	***	Negative	NA	NA	NA	NA	NA	NA	NA	NA	NA
Scu-21	NA	NA	NA	NA	NA	NA	NA	NA	^^	***	Negative	NA	NA	NA	NA	NA	NA	NA	NA	NA
Scu-22	NA	NA	NA	NA	NA	NA	NA	NA	^^	***	Negative	NA	NA	NA	NA	NA	NA	NA	NA	NA
Scu-23	NA	NA	NA	NA	NA	NA	NA	NA	^^	***	Negative	NA	NA	NA	NA	NA	NA	NA	NA	NA
Sch-1	NA	NA	NA	NA	NA	Negative	Negative	NA	NA	Negative	NA	NA	NA	NA	NA	*****	NA	NA	NA	NA
Sch-2	NA	NA	NA	NA	NA	Negative	Negative	NA	NA	Negative	NA	NA	NA	NA	NA	*****	NA	Normal	NA	NA
Sch-3	NA	NA	NA	NA	NA	Negative	Negative	NA	NA	Positive[+++++]	NA	NA	NA	NA	NA	*****	NA	NA	NA	NA
Sch-4	NA	NA	NA	NA	NA	Negative	Negative	NA	NA	Negative	NA	NA	NA	NA	NA	*****	NA	NA	NA	NA
Sch-5	NA	NA	NA	NA	NA	Negative	Negative	NA	NA	Negative	NA	NA	NA	NA	NA	*****	NA	NA	NA	NA
G11-1	NA	NA	NA	NA	NA	NA	NA	NA	NA	NA	NA	NA	NA	NA	NA	NA	NA	NA	NA	NA
G11-2	NA	NA	NA	NA	NA	NA	NA	NA	NA	NA	NA	NA	NA	NA	NA	NA	NA	NA	NA	NA
G11-3	NA	NA	NA	NA	NA	NA	NA	NA	NA	NA	NA	NA	NA	NA	NA	NA	NA	NA	NA	NA
G11-4	NA	NA	NA	NA	NA	NA	NA	NA	NA	NA	NA	NA	NA	NA	NA	NA	NA	NA	NA	NA

G11-5	NA	NA	NA	NA	NA	NA	NA	von Willebrand disease	factor V Leiden	ACL-Abs	NA	NA	NA	NA	NA	NA	NA	NA	NA	NA
G11-6	NA	NA	NA	NA	NA	NA	NA	NA	NA	NA	NA	NA	NA	NA	NA	NA	NA	NA	NA	NA
G11-7	NA	NA	NA	NA	NA	NA	NA	NA	NA	NA	NA	NA	NA	NA	NA	NA	NA	NA	NA	NA
G11-8	NA	NA	NA	NA	NA	NA	NA	NA	NA	NA	NA	NA	NA	NA	NA	NA	NA	NA	NA	NA
G11-9	NA	NA	NA	NA	NA	NA	NA	NA	NA	NA	NA	NA	NA	NA	NA	NA	NA	NA	NA	NA
G11-10	NA	NA	NA	NA	NA	NA	NA	NA	NA	NA	NA	NA	NA	NA	NA	NA	NA	NA	NA	NA
G11-11	NA	NA	NA	NA	NA	NA	NA	NA	NA	NA	NA	NA	NA	NA	NA	NA	NA	NA	NA	NA
Ba1	NA	NA	NA	NA	NA	NA	NA	NA	NA	Negative	NA	NA	NA	NA	NA	NA	NA	NA	IgG antiplatelet antibodies positive<br><br>IgM antiplatelet antibodies borderline	NA
Bl-1	84	46	14	144	NA	0.70<br><br>Ref: 0.70–1.40 IU/ml	0.68<br><br>Ref: 0.55–1.20	NA	Factor V Leiden mutation: Heterozygous	Negative	Antineutrophilic antibodies: 0 (ref<1)	NA	No definite schistocytes	NA	Normal	Normal ******	Not present	Normal	Absent	NA
Meh-1	NA	NA	NA	NA	NA	NA	NA	-	-	NA	NA	NA	no schistocytes on blood film	Thrombocytopenia without cell fragments	NA	NA	NA	NA	NA	NA

Meh-2	NA	NA	NA	NA	NA	NA	NA	-	Factor V Leiden: heterozygous for the c.1601G > A (p.Arg534Gln) variant	Negative	NA	NA	no schistocytes on blood film	Thrombocytopenia without cell fragments	NA	NA	NA	Normal	NA	NA
Cas-1	NA	NA	NA	NA	NA	NA	NA	NA	Methylenetetrahydrofolate reductase (MTHFR) mutation (C677T): Heterozygous	NA	NA	NA	NA	NA	NA	NA	NA	NA	Negative	16.7
D-1	NA	NA	NA	NA	NA	NA	NA	NA	NA	NA	NA	NA	NA	NA	NA	NA	NA	NA	NA	NA
E-1	NA	NA	NA	NA	NA	NA	NA	NA	NA	NA	NA	NA	NA	NA	NA	NA	NA	NA	NA	NA
E-2	NA	NA	NA	NA	NA	146.4%	54.3%	NA	NA	NA	NA	NA	NA	NA	NA	NA	NA	NA	NA	NA
F-1	NA	NA	NA	NA	NA	73<br><br>Ref: 70-140 %	60<br><br>Ref: 72-123 %	Ag: >120, RCo: >150, CB: >150	Factor V Leiden (G169A): absent, Prothrombin (G20210	Negative	NA	NA	<1	NA	NA	C3: 0.76 g/l (Ref: 0.81-1.57), C4: 0.14 g/L (Ref: 0.13-1.39)	NA	48	Negative	16.7

									A): absent, MTHFR (C677T): Heterozygous, JAK2 : absent											
Gar-1	NA	NA	NA	NA	NA	NA	NA	NA	NA	NA	NA	NA	NA	NA	NA	NA	NA	NA	NA	NA
Ge-1	NA	NA	NA	NA	NA	NA	NA	NA	JAK2 V617F mutation was not present	Negative	NA	NA	NA	NA	NA	NA	NA	NA	NA	NA
Ge-2	NA	NA	NA	NA	NA	NA	NA	NA	JAK2 V617F mutation was not present	Negative	NA	NA	NA	NA	NA	NA	NA	NA	NA	NA
GC-1	NA	NA	NA	NA	NA	NA	NA	NA	NA	NA	NA	NA	NA	NA	NA	NA	NA	NA	NA	NA
GC-2	NA	NA	NA	NA	NA	NA	NA	NA	NA	NA	NA	NA	NA	NA	NA	NA	NA	NA	NA	NA
GC-3	NA	NA	NA	NA	NA	NA	NA	NA	NA	NA	NA	NA	NA	NA	NA	NA	NA	NA	NA	NA
GC-4	NA	NA	NA	NA	NA	NA	NA	NA	NA	NA	NA	NA	NA	NA	NA	NA	NA	NA	NA	NA
GC-5	NA	NA	NA	NA	NA	NA	NA	NA	NA	NA	NA	NA	NA	NA	NA	NA	NA	NA	NA	NA
GC-6	NA	NA	NA	NA	NA	NA	NA	NA	NA	NA	NA	NA	NA	NA	NA	NA	NA	NA	NA	NA
GC-7	NA	NA	NA	NA	NA	NA	NA	NA	NA	NA	NA	NA	NA	NA	NA	NA	NA	NA	NA	NA
GC-8	NA	NA	NA	NA	NA	NA	NA	NA	NA	NA	NA	NA	NA	NA	NA	NA	NA	NA	NA	NA
GC-9	NA	NA	NA	NA	NA	NA	NA	NA	NA	NA	NA	NA	NA	NA	NA	NA	NA	NA	NA	NA
GC-10	NA	NA	NA	NA	NA	NA	NA	NA	NA	NA	NA	NA	NA	NA	NA	NA	NA	NA	NA	NA
GC-11	NA	NA	NA	NA	NA	NA	NA	NA	NA	NA	NA	NA	NA	NA	NA	NA	NA	NA	NA	NA

GC-12	NA	NA	NA	NA	NA	NA	NA	NA	NA	NA	NA	NA	NA	NA	NA	NA	NA	NA	NA	NA
GC-13	NA	NA	NA	NA	NA	NA	NA	NA	NA	NA	NA	NA	NA	NA	NA	NA	NA	NA	NA	NA
GC-14	NA	NA	NA	NA	NA	NA	NA	NA	NA	NA	NA	NA	NA	NA	NA	NA	NA	NA	NA	NA
GC-15	NA	NA	NA	NA	NA	NA	NA	NA	NA	NA	NA	NA	NA	NA	NA	NA	NA	NA	NA	NA
GC-16	NA	NA	NA	NA	NA	NA	NA	NA	NA	NA	NA	NA	NA	NA	NA	NA	NA	NA	NA	NA
GC-17	NA	NA	NA	NA	NA	NA	NA	NA	NA	NA	NA	NA	NA	NA	NA	NA	NA	NA	NA	NA
GC-18	NA	NA	NA	NA	NA	NA	NA	NA	NA	NA	NA	NA	NA	NA	NA	NA	NA	NA	NA	NA
GC-19	NA	NA	NA	NA	NA	NA	NA	NA	NA	NA	NA	NA	NA	NA	NA	NA	NA	NA	NA	NA
GC-20	NA	NA	NA	NA	NA	NA	NA	NA	NA	NA	NA	NA	NA	NA	NA	NA	NA	NA	NA	NA
GC-21	NA	NA	NA	NA	NA	NA	NA	NA	NA	NA	NA	NA	NA	NA	NA	NA	NA	NA	NA	NA
GC-22	NA	NA	NA	NA	NA	NA	NA	NA	NA	NA	NA	NA	NA	NA	NA	NA	NA	NA	NA	NA
GC-23	NA	NA	NA	NA	NA	NA	NA	NA	NA	NA	NA	NA	NA	NA	NA	NA	NA	NA	NA	NA
GC-24	NA	NA	NA	NA	NA	NA	NA	NA	NA	NA	NA	NA	NA	NA	NA	NA	NA	NA	NA	NA
GC-25	NA	NA	NA	NA	NA	NA	NA	NA	NA	NA	NA	NA	NA	NA	NA	NA	NA	NA	NA	NA
GC-26	NA	NA	NA	NA	NA	NA	NA	NA	NA	NA	NA	NA	NA	NA	NA	NA	NA	NA	NA	NA
GC-27	NA	NA	NA	NA	NA	NA	NA	NA	NA	NA	NA	NA	NA	NA	NA	NA	NA	NA	NA	NA
J-1	NA	NA	NA	NA	NA	NA	NA	NA	JAK2 V617F mutation negative	Negative	Negative for extractable nuclear antigen, antinuclear antibody, release factor	NA	NA	NA	NA	NA	NA	NA	NA	NA
Ram-1	NA	NA	NA	NA	NA	NA	NA	NA	NA	NA	NA	NA	NA	Thrombocytopenia without polychromasia, spherocytes and fragments	NA	NA	NA	NA	NA	NA

W-1	NA	NA	NA	NA	NA	Normal	Normal	NA	Prothrombin G20210A: wild type, Factor V R5066 (Leiden): wild type, MTHFR: wild type	Negative	antinuclear antibodies: negative	NA	NA	NA	NA	NA	NA	Normal (76%)	Elevated	19.7
W-2	NA	NA	NA	NA	NA	Normal	Normal	NA	Prothrombin G20210A: wild type, Factor V R5066 (Leiden): wild type, MTHFR: NA	Lupus Negative	NA	NA	NA	NA	NA	NA	NA	Normal (73%)	Elevated	5.5
W-3	NA	NA	NA	NA	NA	Normal	Normal	NA	Prothrombin G20210A: wild type, Factor V R5066 (Leiden):	Negative	Standard tests for thrombophilia were negative	NA	NA	NA	NA	NA	NA	Normal (86%)	Elevated	17.1

									wild type, MTHFR: heterozygote mutation (C667T)											
X-1	NA	NA	NA	NA	NA	NA	NA	NA	NA	Negative	NA	NA	NA	NA	NA	NA	NA	NA	NA	NA
Mu-1	NA	NA	NA	NA	NA	NA	NA	Normal	NA	NA	NA	NA	NA	NA	NA	NA	NA	NA	NA	NA
Al-1	NA	NA	NA	NA	NA	NA	NA	NA	NA	NA	NA	NA	NA	Leukocytosis of neutrophils with polychromasia, anisocytosis, and moderate thrombocytopenia	NA	NA	NA	NA	NA	NA
Alt-1	NA	NA	NA	NA	NA	NA	NA	NA	Negative	NA	NA	NA	NA	NA	NA	NA	NA	NA	NA	NA
Alt-2	NA	NA	NA	NA	NA	NA	NA	NA	NA	NA	NA	NA	NA	NA	NA	NA	NA	NA	NA	NA
Alt-3	NA	NA	NA	NA	NA	NA	NA	NA	Heterozygous FVL mutation	NA	NA	NA	NA	NA	NA	NA	NA	NA	NA	NA
Alt-4	NA	NA	NA	NA	NA	NA	NA	NA	Negative	NA	NA	NA	NA	NA	NA	NA	NA	NA	NA	NA
Alt-5	NA	NA	NA	NA	NA	NA	NA	NA	Negative	NA	NA	NA	NA	NA	NA	NA	NA	NA	NA	NA
Alt-6	NA	NA	NA	NA	NA	NA	NA	NA	Negative	NA	NA	NA	NA	NA	NA	NA	NA	NA	NA	NA
Alt-7 Previously reported in wolf study patient code: W-3	NA	NA	NA	NA	NA	NA	NA	NA	NA	NA	NA	NA	NA	NA	NA	NA	NA	NA	NA	NA

Alt-8	NA	NA	NA	NA	NA	NA	NA	NA	Nega tive	NA	NA	NA	NA	NA	NA	NA	NA	NA	NA	NA
Ban-1	NA	NA	NA	NA	NA	NA	NA	NA	NA	Negative	NA	NA	NA	NA	NA	NA	NA	NA	NA	NA
Ban-2	NA	NA	NA	NA	NA	NA	NA	NA	NA	NA	NA	NA	NA	NA	NA	NA	NA	NA	NA	NA
Ban-3	NA	NA	NA	NA	NA	NA	NA	NA	NA	NA	NA	NA	NA	NA	NA	NA	NA	NA	NA	NA
Bon-1	NA	NA	NA	NA	NA	Normal	Normal	NA	NA	Negative	NA	NA	NA	NA	NA	NA	NA	NA	NA	NA
Bour-1	NA	NA	NA	NA	NA	NA	NA	NA	NA	NA	NA	NA	NA	NA	NA	NA	NA	NA	NA	NA
Bour-2	NA	NA	NA	NA	NA	NA	NA	NA	NA	NA	NA	NA	NA	NA	NA	NA	NA	NA	NA	NA
Bour-3	NA	NA	NA	NA	NA	NA	NA	NA	NA	NA	NA	NA	NA	NA	NA	NA	NA	NA	NA	NA
Cho-1	NA	NA	NA	NA	64.6 Ref: < 5.1 µg/mL	NA	NA	NA	NA	NA	NA	NA	NA	Thrombo cytopenia	NA	NA	NA	NA	NA	NA
Cp-1	NA	NA	NA	NA	NA	NA	NA	NA	NA	NA	NA	NA	NA	NA	NA	NA	NA	NA	NA	NA
Cp-2	NA	NA	NA	NA	NA	NA	NA	NA	NA	NA	NA	NA	NA	NA	NA	NA	NA	NA	NA	NA
Cp-3	NA	NA	NA	NA	NA	NA	NA	NA	NA	NA	NA	NA	NA	NA	NA	NA	NA	NA	NA	NA
Gan-1	NA	NA	NA	NA	NA	NA	NA	NA	NA	NA	NA	NA	NA	No hemolysis or fragments of red cell	NA	NA	NA	NA	NA	NA
Gan-2	NA	NA	NA	NA	NA	NA	NA	NA	NA	NA	NA	NA	NA	No hemolysis or fragments of red cell	NA	NA	NA	NA	NA	NA
Gan-3	NA	NA	NA	NA	NA	NA	NA	NA	NA	NA	NA	NA	NA	No hemolysis or fragments of red cell	NA	NA	NA	NA	NA	NA
Gan-4	NA	NA	NA	NA	NA	NA	NA	NA	NA	NA	NA	NA	NA	No hemolysis or fragments of red cell	NA	NA	NA	NA	NA	NA
Gan-5	NA	NA	NA	NA	NA	NA	NA	NA	NA	NA	NA	NA	NA	No hemolysis or fragments of red cell	NA	NA	NA	NA	NA	NA

Gan-6	NA	NA	NA	NA	NA	NA	NA	NA	NA	NA	NA	NA	NA	No hemolysis or fragments of red cell	NA	NA	NA	NA	NA	NA
Gatt-1	NA	NA	NA	NA	NA	Normal	Normal	NA	No genetic or acquired thrombophilia in extensive laboratory evaluations (eg, factor V Leiden or prothrombin mutation	Negative	NA	NA	NA	NA	NA	NA	NA	NA	NA	NA
Gatt-2	NA	NA	NA	NA	NA	Normal	Normal	NA	No genetic or acquired thrombophilia in extensive laboratory evaluations (eg, factor V Leiden or prothrombin	Negative	NA	NA	NA	NA	NA	NA	NA	NA	NA	NA

									mutation											
Gess-1	NA	NA	NA	NA	NA	NA	NA	NA	NA	NA	NA	NA	NA	NA	NA	NA	NA	NA	NA	NA
Gess-2	NA	NA	NA	NA	NA	NA	NA	NA	NA	NA	NA	NA	NA	NA	NA	NA	NA	NA	NA	NA
Gra-1	NA	NA	NA	NA	NA	NA	NA	NA	NA	NA	NA	NA	NA	NA	NA	NA	NA	NA	NA	NA
Gua-1	NA	NA	NA	NA	NA	NA	NA	NA	NA	NA	NA	NA	NA	NA	NA	NA	NA	NA	NA	NA
H-1	NA	NA	NA	NA	NA	NA	NA	NA	NA	NA	NA	NA	NA	NA	NA	NA	NA	NA	NA	NA
Ik-1	NA	NA	NA	NA	NA	NA	NA	NA	NA	NA	NA	NA	NA	NA	NA	NA	NA	NA	NA	NA
Ja-1	NA	NA	NA	NA	NA	NA	NA	NA	NA	++++++	ANA Negative	NA	NA	NA	+++++++	NA	NA	NA	NA	NA
Kh-1	NA	NA	NA	NA	NA	NA	NA	NA	NA	Negative	NA	NA	NA	NA	++++++++	NA	NA	NA	NA	NA
Ma-1	NA	NA	NA	NA	NA	NA	NA	NA	no significant genetic alterations in genes associated to thrombocytopathies, complementopathies, and platelets disfunction diseases	+++++++++	««	NA	NA	NA	NA	NA	NA	NA	NA	NA
Mi-1	NA	NA	NA	NA	NA	NA	NA	increased circu	Negative	Negative	ANA negative	Observed	NA	Giant platelets	NA	NA	NA	NA	NA	NA

								lating levels of von Willebrand Factor Antigen (VWF:Ag) and an increase in the levels of VWF-Ristocetin Cofactor (VWF:RCo).												
Mi-2	NA	NA	NA	NA	NA	NA	NA	increased circulating levels of von Willebrand Factor Antigen (VWF:Ag) and an increase	Negative	Negative	ANA negative	NA	NA	Platelet anisocytosis	NA	NA	NA	NA	NA	NA

								in the levels of VWF-Ristocetin Cofactor (VWF:RCo).												
Ps-1	NA	NA	NA	NA	NA	Negative	Negative	NA	thrombophilia (Factor V Leiden R506Q mutation, Prothrombin G20210A gene mutation negative	Negative	NA	NA	NA	Normal blood smear	NA	NA	NA	NA	NA	NA
Pat-1	NA	NA	NA	NA	NA	NA	NA	NA	NA	NA	NA	NA	NA	NA	NA	NA	NA	NA	NA	NA
Pat-2	NA	NA	NA	NA	NA	NA	NA	NA	NA	NA	NA	NA	NA	NA	NA	NA	NA	NA	NA	NA
Pat-3	NA	NA	NA	NA	NA	NA	NA	NA	NA	NA	NA	NA	NA	NA	NA	NA	NA	NA	NA	NA
Pal-1	NA	NA	NA	NA	NA	NA	NA	Positive	NA	NA	NA	NA	4-5%	NA	NA	NA	NA	Severe deficiency	Absent	NA
R-1	NA	NA	NA	NA	NA	NA	NA	NA	NA	NA	©©©©	NA	NA	NA	NA	NA	NA	NA	NA	NA
Sol-1	NA	NA	NA	NA	NA	NA	NA	NA	NA	Lupus positive<br><br>ACL and anti-β2 GPr 1 Negative	NA	NA	NA	Normal	NA	NA	NA	Normal	NA	NA

Sol-2	NA	NA	NA	NA	NA	NA	NA	NA	NA	NA	NA	NA	NA	No fragments or platelet clumps	NA	NA	NA	NA	NA	NA
Sol-3	NA	NA	NA	NA	NA	NA	NA	NA	NA	NA	NA	NA	NA	NA	NA	NA	NA	NA	NA	NA
Sør-1	NA	NA	NA	NA	NA	1.20±	.85 ±±	Positive	heterozygosity for the prothrombin G20210A mutation; otherwise. no variants were found<br><br>Factor V Leiden R506Q mutation not detected	ACL IgG/IgM < 2.2/2.5 ±±±<br><br>b-2-GP1 IgG/IgM <0.6/0.9 ±±±±<br><br>Lupus NA	ANA 0.1<br><br>Anti–double-stranded DNA IgG 3.7<br><br>Anti-MPO negative<br><br>Others negative<br><br>■■■	NA	No schistocytes	NA	NA	C3 1.42<br><br>C4 0.345<br><br>▤	NA	1.07 $$$	NA	6.2 $$$$
Sur-1	NA	NA	NA	NA	NA	NA	NA	NA	NA	NA	NA	NA	NA	NA	NA	NA	NA	NA	NA	NA
Tej-1	NA	NA	NA	NA	NA	NA	NA	NA	NA	NA	NA	NA	NA	NA	NA	NA	NA	NA	NA	NA
Ti-1	NA	NA	NA	NA	NA	Negative	Negative	NA	NA	Lupus Negative	NA	NA	Abundant schistocytes	NA	NA	NA	NA	0.92 Normal	NA	NA
Ti-2	NA	NA	NA	NA	NA	NA	NA	NA	NA	NA	NA	NA	0.7 day 1	NA	NA	NA	NA	NA	NA	NA

Ti-3	NA	NA	NA	NA	NA	NA	NA	NA	NA	NA	NA	NA	0 day 1	NA	NA	CH50 <10%<br><br>Day 1<br><br>ȚȚ	NA	NA	NA	NA
Ti-4	NA	NA	NA	NA	NA	NA	NA	NA	NA	NA	NA	NA	Very few day 1	NA	NA	CH50 <10%<br><br>Day 7<br><br>ȚȚ	NA	NA	NA	NA
Ti-5	NA	NA	NA	NA	NA	NA	NA	NA	NA	NA	NA	NA	0.8 day 3	NA	NA	CH50 >60%<br><br>Day 7<br><br>ȚȚ	NA	NA	NA	NA
Tu-1	NA	NA	NA	NA	NA	Negative	Negative	NA	Genetic tests for factor V Leiden, prothrombin and *JAK2* mutations were negative<br><br>paroxysmal nocturnal haemoglobinuria phenotype was not present.	Negative	NA	NA	NA	NA	NA	NA	NA	80.7% Normal	NA	Normal

Var-1	NA	NA	NA	NA	NA	NA	NA	NA	NA	NA	NA	NA	NA	NA	NA	NA	NA	NA	NA	NA
Vay-1	NA	NA	NA	NA	NA	NA	NA	NA	NA	NA	NA	NA	NA	NA	NA	NA	NA	NA	NA	NA
Vay-2	NA	NA	NA	NA	NA	NA	NA	NA	NA	NA	NA	NA	NA	NA	NA	NA	NA	NA	NA	NA
Vay-3	NA	NA	NA	NA	NA	NA	NA	NA	NA	NA	NA	NA	NA	NA	NA	NA	NA	NA	NA	NA
Vay-4	NA	NA	NA	NA	NA	NA	NA	NA	NA	NA	NA	NA	NA	NA	NA	NA	NA	NA	NA	NA
Vay-5	NA	NA	NA	NA	NA	NA	NA	NA	NA	NA	NA	NA	NA	NA	NA	NA	NA	NA	NA	NA
Vay-6	NA	NA	NA	NA	NA	NA	NA	NA	NA	NA	NA	NA	NA	NA	NA	NA	NA	NA	NA	NA
Vay-7	NA	NA	NA	NA	NA	NA	NA	NA	NA	NA	NA	NA	NA	NA	NA	NA	NA	NA	NA	NA
Vay-8	NA	NA	NA	NA	NA	NA	NA	NA	NA	NA	NA	NA	NA	NA	NA	NA	NA	NA	NA	NA
Vay-9	NA	NA	NA	NA	NA	NA	NA	NA	NA	NA	NA	NA	NA	NA	NA	NA	NA	NA	NA	NA
Wan-1	NA	NA	NA	NA	NA	NA	NA	NA	NA	NA	NA	NA	NA	NA	NA	NA	NA	NA	NA	NA
Wi-1	NA	NA	NA	NA	NA	NA	NA	NA	NA	NA	NA	NA	NA	NA	NA	NA	NA	NA	NA	NA
Z-1	NA	NA	NA	NA	NA	Negative	Negative	NA	NA	ACL (IgM-IgG) Negative	Anti-nuclear antibodies Negative<br><br>Extractable nuclear antigen Negative	NA	NA	NA	NA	NA	NA	NA	NA	Negative

*** A test for lupus anticoagulant was positive in 5 of the 10 patients for whom results were available, but in the context of severe coagulopathy and negative tests for anticardiolipin antibodies and anti–β2 -glycoprotein 1b antibodies, these results were considered to be unreliable.
***** Levels of complement proteins (C1q, C4, and C3) and activation products (sC5b-9) were within the normal range in all patients.
****** Complement C3c/C4 was assessed in Blauenfeldt-2021 study
++++ Two patients had pre-existing autoimmune disease. 1 patient, demyelinating disease; 1 patient, antiphospholipid antibodies.
+++++ Antiphospholipid antibodies were detected only in Patient 3, who had a slightly elevated anticardiolipin IgG antibody level of 43 IgG phospholipid (GPL) units
++++++ anticardiolipin G <1.6 U/mL (Ref: 0–20 U/mL); anticardiolipin M 0.5 U/mL (Ref: 0–20 U/mL); Lupus anticoagulant ratio: 1.28 with no Ref (Jacob-2021).
+++++++ Serum electrophoresis: Band in gammaglobulin region – IgG (Lambda) abnormality with monoclonal component of 2.2 g/L (Jacob-2021).
++++++++ NS-1 antigen, dengue IgM, and IgG serologic testing were negative (Khuhapinant-2021).
+++++++++ Anticardiolipin IgG: 50.2 U/mL (Ref: Positive> 20), Anticardiolipin IgM: 1.7 U/mL (Ref: Negative), IgG Beta-2 Glycoprotein 1 Antibodies: 12,3 U/mL (Ref: Negative), IgM Beta-2 Glycoprotein 1 Antibodies: 2,8 U/mL (Ref: Negative) (Mauriello-2021).
«« ENA7, Extractable Nuclear Antigen Antibodies: 17,6 CU (Ref: Negative), Myeloperoxidase: 3,8 CU (Ref: Negative), PR3, Proteinase 3 antineutrophil cytoplasmic: < 2,3 CU (Ref: Negative), dsDNA: 12,9 IU/mL(Ref: Negative) (Mauriello-2021).
α extrinsic pathway (tissue factor) clotting time, Ref: < 74 sec (Blauenfeldt-2021),
β amplitude of formed clot 10 min after formation, Ref: > 48 mm (Blauenfeldt-2021),
χ fibrin-dependent clot formation, amplitude of the formed clot at 10 min. Ref: >8 mm (Blauenfeldt-2021),
δ intrinsic pathway clotting time, Ref: <121 sec (Blauenfeldt-2021),
O Ref: <1% (Franchini-2021), <1% (Tiede-2021).

©©©© antinuclear antibodies, antineutrophil cytoplasmic antibodies were normal (Ramessur-2021).
b Autoimmune screening tested positive for lupus anti-coagulant (LA) with a dilute Russell viper venom time ratio (DRVVTr) of 1.78, but negative for anti-cardiolipin antibodies, anti-b2-glycoprotein, anti-thyroglobulin, anti-thyroid peroxidase, anti-neutrophil cytoplasmic anti-bodies (ANCA), rheumatoid factor, and anti-nuclear anti-bodies.
Θ Ref: < 1 (Blauenfeldt-2021),
ƒ a disintegrin and metalloproteinase with a thrombospondin type 1 motif, member 13, ADAMTS13 activity (FRET assay) (%) Ref: 45-138 (Franchini-2021)
⊥ Antibodies against GP-IIb/IIIa, GP-Ia/IIa, GP-Ib/IX, and GP-IV (Blauenfeldt-2021),
w Ref: < 12 µmol/L (Castelli-2021, Franchini—2021)

■■ positive lupus anticoagulant but did not have anticardiolipin or anti-β2 glycoprotein-1 antibodies (Soleimani-2021).

■■■ Anti-nuclear antibodies and anti-neutrophil cytoplasmatic antibodies were negative Antinuclear IgG (ref., ,0.1). anti-globulin was test negative, Anti–double-stranded DNA IgG, kIU/L (ref., , 10) others Anti-Smith, anti-(U1) snRNP, anti-SSA, anti-SSB, anti-scl-70, anti-Jo1, anti-PR3, anti-glomerular basement membrane, and immunoglobulin G were negative (Sørensen-2021).

▤ Complement screening analysis (Complement System Screen WIESLAB; Euro-Diagnostica, Malm€o, Sweden) demonstrated severely decreased activation of the classic pathway and slightly decreased activation of the lectin pathway, consistent with antibody-mediated consumption of complement proteins. Concentrations of complement C3 and C4 were within normal range; C3, g/L (ref., 0.811-1.570), C4, g/L (ref., 0.129-0.392) (Sørensen-2021).

▤▤ Anti-platelet antibodies were detected against glycoprotein GPIIb- IIIa and GPIa-IIa but were inconclusive regarding human platelet– specific antibodies (Pak Lx Assay; Immucor, Waukesha, WI) (Sørensen-2021).

$$$ ADAMTS13 protein: 1.07 kIU/L (Ref 0.61-1.31) (Sørensen-2021).

$$$$Homocysteine, mmol/L (Ref < 15.0) (Sørensen-2021).

± Protein C *10^3 IU/L (ref. 0.70-1.30) (Sørensen-2021).

±±Protein S*10^3 , IU/L (ref 0.57-1.30) (Sørensen-2021).

±±±Cardiolipin IgG/IgM* 10^3 IU/L (Ref<10.0) (Sørensen-2021).

±±±±b-2-GP1 IgG/IgM, kU/L (Ref 0-10) (Sørensen-2021).

ŢŢ ref. range 32-58%, (Tiede-2021).

+++ two patients had an underlying coagulation disorder (1 patient, von Willebrand disease; 1 patient, “unspecified coagulation disorder”), Grainacher 2021.
^^ No other relevant laboratory tests were positive, including tests for thrombophilia, antinuclear antibodies, extractable nuclear antigen, and antiphospholipid antibodies, Scully 2021.

Table S5. Details of anti-Pf4 antibody assays of case reports on VITT after ChAdOx1 nCov-19 vaccination.

	Immunoassays	Functional assays		
**Patient code**	**Anti-PF4 antibodies**	**Heparin-induced platelet activation (HIPA) assay**	**Serotonin-release assay (SRA)**<br>**14C SRA; EIA SRA ; HPLC SRA**	**Modified HIPA assay**
G9-1	4 patients positive immunoassay OD> 3.00, EIA, (reference value, < 0.50)	4 patient sera activated platelets; regarded as positive	NA	NA
G9-2			NA	NA
G9-3			NA	NA
G9-4			NA	NA
G9-5			NA	NA
G9-6			NA	NA
G9-7			NA	NA
G9-8			NA	NA
G9-9			NA	NA
A1	Asserachrom HPIA IgG assay for anti-PF4 antibodies; positive; (76.1%)	NA	NA	NA
A2	Anti-PF4 antibody assay:positive; (99.7%)	NA	NA	NA
A3	Anti-PF4 antibody assay: positive	NA	NA	NA
Scu-1	HemosIL AcuStar HIT IgG Assay: Negative; Ref: Negative<br>Asserachrom HPIA IgG Assay: 0.776 ; Ref: < 0.238<br>Lifecodes PF4 IgG Assay: ND Ref: <0.4	NA	(HITAlert, Diapharma)<br>Functional HIT Assay: Positive	NA
Scu-2	HemosIL AcuStar HIT IgG Assay: ND<br>Asserachrom HPIA IgG Assay: 1.310<br>Lifecodes PF4 IgG Assay: ND	NA	(HITAlert, Diapharma)<br>Functional HIT Assay: ND	NA
Scu-3	HemosIL AcuStar HIT IgG Assay: ND<br>Asserachrom HPIA IgG Assay: ND<br>Lifecodes PF4 IgG Assay: 2.45	NA	(HITAlert, Diapharma)<br>Functional HIT Assay: ND	NA
Scu-4	HemosIL AcuStar HIT IgG Assay: ND<br>Asserachrom HPIA IgG Assay: ND<br>Lifecodes PF4 IgG Assay: 2.26	NA	(HITAlert, Diapharma)<br>Functional HIT Assay: ND	NA

Scu-5	HemosIL AcuStar HIT IgG Assay: Negative<br>Asserachrom HPIA IgG Assay: ND<br>Lifecodes PF4 IgG Assay: 2.84	NA	(HITAlert, Diapharma)<br>Functional HIT Assay: Negative	NA
Scu-6	HemosIL AcuStar HIT IgG Assay: Negative<br>Asserachrom HPIA IgG Assay: 0.297<br>Lifecodes PF4 IgG Assay: ND	NA	(HITAlert,<br>Diapharma)<br>Functional HIT Assay: ND	NA
Scu-7	HemosIL AcuStar HIT IgG Assay: ND<br>Asserachrom HPIA IgG Assay: 0.297<br>Lifecodes PF4 IgG Assay: ND	NA	(HITAlert, Diapharma)<br>Functional HIT Assay: ND	NA
Scu-8	HemosIL AcuStar HIT IgG Assay: Negative<br>Asserachrom HPIA IgG Assay:1.44<br>Lifecodes PF4 IgG Assay: ND	NA	(HITAlert, Diapharma)<br>Functional HIT Assay: ND	NA
Scu-9	HemosIL AcuStar HIT IgG Assay: Negative<br>Asserachrom HPIA IgG Assay: 1.070<br>Lifecodes PF4 IgG Assay: ND	NA	(HITAlert, Diapharma)<br>Functional HIT Assay: ND	NA
Scu-10	HemosIL AcuStar HIT IgG Assay: Negative<br>Asserachrom HPIA IgG Assay: 1.640<br>Lifecodes PF4 IgG Assay: ND	NA	(HITAlert,Diapharma)<br>Functional HIT Assay: ND	NA
Scu-11	HemosIL AcuStar HIT IgG Assay: Negative<br>Asserachrom HPIA IgG Assay: 1.156<br>Lifecodes PF4 IgG Assay: ND	NA	(HITAlert, Diapharma)<br>Functional HIT Assay: ND	NA
Scu-12	HemosIL AcuStar HIT IgG Assay: Negative<br>Asserachrom HPIA IgG Assay: ND<br>Lifecodes PF4 IgG Assay: Positive	NA	(HITAlert, Diapharma)<br>Functional HIT Assay: ND	NA
Scu-13	HemosIL AcuStar HIT IgG Assay: ND<br>Asserachrom HPIA IgG Assay: ND<br>Lifecodes PF4 IgG Assay: 0.76	NA	(HITAlert, Diapharma)<br>Functional HIT Assay: ND	NA
Scu-14	HemosIL AcuStar HIT IgG Assay: Negative<br>Asserachrom HPIA IgG Assay:ND<br>Lifecodes PF4 IgG Assay: Positive	NA	(HITAlert, Diapharma)<br>Functional HIT Assay: ND	NA

Scu-15	HemosIL AcuStar HIT IgG Assay: ND<br>Asserachrom HPIA IgG Assay: ND<br>Lifecodes PF4 IgG Assay: 1.40	NA	(HITAlert, Diapharma)<br>Functional HIT Assay: ND	NA
Scu-16	HemosIL AcuStar HIT IgG Assay: ND<br>Asserachrom HPIA IgG Assay: ND<br>Lifecodes PF4 IgG Assay: 1.40	NA	(HITAlert, Diapharma)<br>Functional HIT Assay: ND	NA
Scu-17	HemosIL AcuStar HIT IgG Assay: ND<br>Asserachrom HPIA IgG Assay: ND<br>Lifecodes PF4 IgG Assay: Positive	NA	(HITAlert, Diapharma)<br>Functional HIT Assay: ND	NA
Scu-18	HemosIL AcuStar HIT IgG Assay: ND<br>Asserachrom HPIA IgG Assay: ND<br>Lifecodes PF4 IgG Assay: 2.80	NA	(HITAlert, Diapharma)<br>Functional HIT Assay: Positive	NA
Scu-19	HemosIL AcuStar HIT IgG Assay: ND<br>Asserachrom HPIA IgG Assay: ND<br>Lifecodes PF4 IgG Assay: >3.00	NA	(HITAlert, Diapharma)<br>Functional HIT Assay: ND	NA
Scu-20	HemosIL AcuStar HIT IgG Assay: ND<br>Asserachrom HPIA IgG Assay: ND<br>Lifecodes PF4 IgG Assay: 2.17	NA	(HITAlert, Diapharma)<br>Functional HIT Assay: Positive	NA
Scu-21	HemosIL AcuStar HIT IgG Assay: ND<br>Asserachrom HPIA IgG Assay: ND<br>Lifecodes PF4 IgG Assay: 2.45	NA	(HITAlert, Diapharma)<br>Functional HIT Assay: Positive	NA
Scu-22	HemosIL AcuStar HIT IgG Assay: ND<br>Asserachrom HPIA IgG Assay: ND<br>Lifecodes PF4 IgG Assay: >3.00	NA	(HITAlert, Diapharma)<br>Functional HIT Assay: Positive	NA
Scu-23	HemosIL AcuStar HIT IgG Assay: ND<br>Asserachrom HPIA IgG Assay: ND<br>Lifecodes PF4 IgG Assay: Negative	NA	(HITAlert, Diapharma)<br>Functional HIT Assay: Negative	NA
Sch-1	LIFECODES (ELISA) (Immucor)<br>High optical density values in the range of 2.9 to 3.8	NA	Heparin-induced multiple-electrode aggregometry on a Multiplate analyzer (Dynabyte Medical) Platelets in Patients 1, 3, 4, and 5 were clearly activated in the absence of added heparin	NA

Sch-2	LIFECODES (ELISA) (Immucor)<br><br>High optical density values in the range of 2.9 to 3.8	NA	Heparin-induced multiple-electrode aggregometry on a Multiplate analyzer (Dynabyte Medical); inconclusive	NA
Sch-3	LIFECODES (ELISA) (Immucor)<br><br>High optical density values in the range of 2.9 to 3.8	NA	Heparin-induced multiple-electrode aggregometry on a Multiplate analyzer (Dynabyte Medical) Platelets in Patients 1, 3, 4, and 5 were clearly activated in the absence of added heparin	NA
Sch-4	LIFECODES (ELISA) (Immucor)<br><br>High optical density values in the range of 2.9 to 3.8	NA	Heparin-induced multiple-electrode aggregometry on a Multiplate analyzer (Dynabyte Medical) Platelets in Patients 1, 3, 4, and 5 were clearly activated in the absence of added heparin	NA
Sch-5	LIFECODES (ELISA) (Immucor)<br><br>High optical density values in the range of 2.9 to 3.8	NA	Heparin-induced multiple-electrode aggregometry on a Multiplate analyzer (Dynabyte Medical) Platelets in Patients 1, 3, 4, and 5 were clearly activated in the absence of added heparin	NA
G11-1	PF4–heparin ELISA (optical density): 3.16	PF4-dependent platelet activation assay: Positive	NA	Modified platelet-activation test: Positive
G11-2	PF4–heparin ELISA (optical density): 3.08	PF4-dependent platelet activation assay: Positive	NA	Modified platelet-activation test: Positive
G11-3	PF4–heparin ELISA (optical density): 3.50	PF4-dependent platelet activation assay: Positive	NA	Modified platelet-activation test: Positive
G11-4	PF4–heparin ELISA (optical density): 3.40	PF4-dependent platelet activation assay: Positive	NA	Modified platelet-activation test: Positive
G11-5	PF4–heparin ELISA (optical density): 1.20	PF4-dependent platelet activation assay: Positive	NA	Modified platelet-activation test: Positive
G11-6	PF4–heparin ELISA (optical density): NA	PF4-dependent platelet activation assay: NA	NA	Modified platelet-activation test: Positive

G11-7	PF4–heparin ELISA (optical density): NA	PF4-dependent platelet activation assay: NA	NA	Modified platelet-activation test: Positive
G11-8	PF4–heparin ELISA (optical density): 2.02	PF4-dependent platelet activation assay: Positive	NA	Modified platelet-activation test: Positive
G11-9	PF4–heparin ELISA (optical density): 3.51	PF4-dependent platelet activation assay: Positive	NA	Modified platelet-activation test: Positive
G11-10	PF4–heparin ELISA (optical density): 2.35	PF4-dependent platelet activation assay: Positive	NA	Modified platelet-activation test: Positive
G11-11	PF4–heparin ELISA (optical density): 2.16	PF4-dependent platelet activation assay: Positive	NA	Modified platelet-activation test: Positive
Ba1	Lateral flow immunoassay: Negative	NA	NA	NA
Bl-1	Anti-PF- 4 IgG: positive ELISA	NA	NA	NA
Meh-1	-	NA	NA	NA
Meh-2	Platelet factor-4 antibodies: Positive	NA	NA	NA
Cas-1	NA	NA	NA	NA
D-1	NA	NA	NA	NA
E-1	NA	NA	NA	NA
E-2	Heparin-induced thrombocytopenia: negative	NA	NA	NA
F-1	PF4 ELISA, Immucor: 2.6 (Ref: <0.4)	NA	NA	NA
Gar-1	Lifecodes PF4 IgG Assay, Immucor® positive	NA	Functional platelet activation :Positive	NA
Ge-1	Latex Immunoturbidimetric Assay HemosIL1 HIT-Ab (PF4-H): Negative<br>LIFECODES PF4 IgG, Immucor1: Positive	NA	Serotonin Release Assay: Positive	NA
Ge-2	Latex Immunoturbidimetric Assay HemosIL1 HIT-Ab (PF4-H): Negative<br>LIFECODES PF4 IgG, Immucor1: Positive	NA	Serotonin Release Assay: Positive	NA
GC-1	Yes	NA	NA	NA
GC-2	Yes	NA	NA	NA

GC-3	Yes	NA	NA	NA
GC-4	Yes	NA	NA	NA
GC-5	Yes	NA	NA	NA
GC-6	Yes	NA	NA	NA
GC-7	Yes	NA	NA	NA
GC-8	No	NA	NA	NA
GC-9	Yes	NA	NA	NA
GC-10	Yes	NA	NA	NA
GC-11	Yes	NA	NA	NA
GC-12	Yes	NA	NA	NA
GC-13	No	NA	NA	NA
GC-14	No	NA	NA	NA
GC-15	ND	NA	NA	NA
GC-16	Yes	NA	NA	NA
GC-17	ND	NA	NA	NA
GC-18	No	NA	NA	NA
GC-19	ND	NA	NA	NA
GC-20	ND	NA	NA	NA
GC-21	ND	NA	NA	NA
GC-22	ND	NA	NA	NA
GC-23	No	NA	NA	NA
GC-24	ND	NA	NA	NA
GC-25	ND	NA	NA	NA
GC-26	ND	NA	NA	NA
GC-27	ND	NA	NA	NA
J-1	HemosIL HIT-Ab (PF4-H): Negative	NA	SRA positive	NA
Ram-1	Anti-PF4 antibody assay was 2.509 (normal range 0–0.4)	NA	NA	NA
W-1	HIT platelet factor 4 antibody (PF4)-IgG EIA (Immucor, Dreieich, Germany): Negative<br>ELISA: Positive	HIPA-Ab: negative	NA	NA

W-2	HIT Negative ELISA Positive	HIPA-Ab: negative	NA	NA
W-3	HIT Negative ELISA Positive	HIPA-Ab: negative	NA	NA
X-1	NA	NA	NA	NA
Mu-1	NA	NA	NA	NA
Al-1	NA	NA	NA	NA
Alt-1	EIA (optical density [OD] 2.59±0.64) As range for all of them Versus control 0.176±0.073<br><br>In-house EIA (OD IgG antibodies against PF4: 1.03±0.04 *vs.* 0.110±0.002	NA	NA	One of the sera from patients with severe COVID-19 who were tested positive in PF4/heparin EIA showed platelet activation in the HIPA assay
Alt-2	EIA positive In-house EIA positive	NA	NA	NA
Alt-3	EIA positive In-house EIA positive	NA	NA	NA
Alt-4	EIA positive In-house EIA positive	NA	NA	NA
Alt-5	EIA positive In-house EIA positive	NA	NA	NA
Alt-6	EIA positive	NA	NA	NA
Alt-7	NA	NA	NA	NA
Alt-8	In-house EIA positive	NA	NA	NA
Ban-1	Rapid particle gel immunoassay (ID-PaGIA Heparin/PF4 Antibody Test, Bio-Rad): positive<br><br>HIT ELISA: positive<br><br>ELISA immunoassays (optical density (OD) 2.871; cut-off for positive reaction >0.4).	NA	Platelet functional assay positive	NA
Ban-2	Rapid particle gel immunoassay: negative<br><br>HIT ELISA : positive (OD 2.631)<br><br>cut-off for positive reaction >0.4).	NA	Platelet functional assay negative	NA
Ban-3	HIT ELISA<br><br>positive (OD=2.423)<br><br>(optical density (cut-off<br><br>for positive reaction >0.400))	NA	platelet functional assay negative	NA

Bon-1	ELISA (Immucor, Waukesha, WI, USA).<br><br>ELISA (OD=1.918, reference value <0.4)	NA	Platelet-activation test (PAT) positive	NA
Bour-1	(LIFECODES PF4 IgG/IgA/IgM enzyme-linked immunosorbent assay [ELISA], Immucor): 2.70	NA	14C SRA: weakly positive for HIT	NA
Bour-2	(LIFECODES PF4 IgG/IgA/IgM enzyme-linked immunosorbent assay [ELISA], Immucor): 1.78	NA	14C SRA: atypical result, with 35% serotonin release observed in the absence of heparin and was inhibited with the addition of heparin	NA
Bour-3	(LIFECODES PF4 IgG/IgA/IgM enzyme-linked immunosorbent assay [ELISA], Immucor): 2.69	NA	14C SRA: atypical result, with serotonin release of 78% with heparin	NA
Cho-1	PF4 ELISA Ab test: positive (0.72, optical-density units, Lifecodes PF4 IgG assay (Immucor Inc., Norcross, GA, USA) [normal range, < 0.4])	NA	NA	NA
Cp-1	Anti-PF4 antibodies: positive 1.35 (normal range <0.4)	NA	NA	NA
Cp-2	Positive anti-PF4 antibodies	NA	NA	NA
Cp-3	Positive anti-PF4 antibodies	NA	NA	NA
Gan-1	HIT ELISA : OD: 3.5	NA	NA	NA
Gan-2	HIT ELISA : OD: 2.2	NA	NA	NA
Gan-3	HIT ELISA : OD: 3	NA	NA	NA
Gan-4	HIT ELISA : OD: 2.04	NA	NA	NA
Gan-5	HIT ELISA : OD: 2.5	NA	NA	NA
Gan-6	HIT ELISA : Positive	NA	NA	NA
Gatt-1	Negative HemosIL HIT IgG	NA	NA	NA
Gatt-2	Negative HemosIL HIT IgG	NA	NA	NA
Gess-1	NA	NA	NA	NA
Gess-2	NA	NA	NA	NA
Gra-1	NA	NA	NA	NA
Gua-1	(PF-4) (ELLISA) Positive	NA	NA	NA
H-1	Anti-PF4/polyanion ELISA (MyBioSource, San Diego, CA, USA): positive<br><br>1.214 OD (normal range ≤ 0.399)	NA	NA	NA
Ik-1	(HIT) Positive<br><br>Particle gel agglutination immunoassay ID-PaGIA	(HIPA) : POSITIVE	NA	(PIPA, a modified HIPA test) Positive

Ja-1	HIT ELISA (Immucor) LIFECODES PF4 optical density 2.4566; POSITIVE (cut off for positive result >0.4)<br><br>HIT chemiluminescent immunoassay (HemosILVR AcuStar HIT); NEGATIVE	NA	NA	NA
Kh-1	(PF4) ELISA (Zymutest HIA IgG, HYPHEN BioMed, Neuville-sur-Oise, France): positive (OD) of 2.10 (normal OD, <0.4)	(AggRAM Analyzer®, Helena Laboratories, Beaumont, Texas).<br><br>HIPA positive at low heparin concentration	NA	NA
Ma-1	Undetectable anti-PF4 antibodies HIT IgG (anti PF4)<br><br>Result 0,38 U/mL<br><br>Ref: ≤ 1,00 U/mL	NA	NA	NA
Mi-1	High levels (IgG, IgM, and IgA) to PF4 not positive (IgG/IgA/IgM, Immucor, Lifecodes, Waukesha, WI)	NA	Functional test : Positive	NA
Mi-2	High levels of (IgG, IgM, and IgA) to PF4 positive (1.29 OD405), (IgG/IgA/IgM, Immucor, Lifecodes, Waukesha, WI).	NA	Functional test : Positive	NA
Ps-1	Particle gel immune assay (ID-PaGIA Heparin/PF4 Antibody test) positive	NA	NA	NA
Pat-1	OD : PF4 ELISA 2.38	NA	Positive PF4 SRA	NA
Pat-2	OD : PF4 ELISA 2.06	NA	Positive PF4 SRA	NA
Pat-3	OD : PF4 ELISA 2.28	NA	Positive PF4 SRA	NA
Pal-1	(PF4) antibodies Negative HIT	NA	NA	NA
R-1	NA	NA	NA	NA
Sol-1	AcuStarHIT-IgG negative<br><br>STAGO Asserachrom HPIA ELISA (PF4) antibodies positive	NA	NA	NA
Sol-2	AcustarHIT negative<br><br>Anti-PF4 ELISA positive	NA	NA	NA
Sol-3	AcutstarHIT negative<br><br>Anti-PF4 ELISA negative	NA	NA	NA
Sør-1	ELISA OD 2.2 (upper limit of normal,%0.4 (LIFECODES PF4 IgG by Immucor, Norcross, Georgia)	HIPA negative	NA	NA
Sur-1	PF4 antibodies Positive OD: 3.125 (cut-off for positive reactions >0.400).	NA	NA	NA

Tej-1	(HIT) positive OD 1.23	NA	SRA Positive	NA
Ti-1	The **CLIA** (ACL AcuStar HIT IgG, Werfen, Munich, Germany) negative<br>**ELISA** positive<br>Zymutest HIT IgG (Hyphen BioMed, CoaChrom, Maria Enzersdorf, Austria)<br>Lifecodes PF4 IgG (Immucor GTI Diagnostics, Rodermark, Germany)	NA	SRA Negative	Negative
Ti-2	The **CLIA** (ACL AcuStar HIT IgG, Werfen, Munich, Germany) negative<br>**ELISA** positive<br>Zymutest HIT IgG (Hyphen BioMed, CoaChrom, Maria Enzersdorf, Austria)<br>Lifecodes PF4 IgG (Immucor GTI Diagnostics, Rodermark, Germany)	NA	SRA Negative	Negative
Ti-3	The **CLIA** (ACL AcuStar HIT IgG, Werfen, Munich, Germany) negative<br>**ELISA** positive<br>Zymutest HIT IgG (Hyphen BioMed, CoaChrom, Maria Enzersdorf, Austria)<br>Lifecodes PF4 IgG (Immucor GTI Diagnostics, Rodermark, Germany).	NA	SRA Negative	Negative
Ti-4	The **CLIA** (ACL AcuStar HIT IgG, Werfen, Munich, Germany) negative<br>**ELISA** positive<br>Zymutest HIT IgG (Hyphen BioMed, CoaChrom, Maria Enzersdorf, Austria)<br>Lifecodes PF4 IgG (Immucor GTI Diagnostics, Rodermark, Germany)	NA	SRA Negative	Negative
Ti-5	The **CLIA** (ACL AcuStar HIT IgG, Werfen, Munich, Germany) negative<br>**ELISA** positive<br>Zymutest HIT IgG (Hyphen BioMed, CoaChrom, Maria Enzersdorf, Austria)<br>Lifecodes PF4 IgG (Immucor GTI Diagnostics, Rodermark, Germany)	NA	SRA Negative	Negative
Tu-1	chemiluminescence (Hemosil AcuStar HIT-IgGPF4-H; IL, Bedford, MA, USA) negative<br>ELISA negative OD:0.04 (cut-off: 1.0)	HIPA positive	NA	NA
Var-1	(PF4) antibody positive	NA	NA	NA
Vay-1	STIC EXPERT HIT Negative<br>ID PaGIA H/PF4 ND<br>Hemosil AcuStar HIT-IgG Negative<br>Hemosil HIT-Ab ND<br>PVS/PF4 Elisa OD: 1.90	NA	Standard SRA: POSITIVE<br>SRA-PF4: POSITIVE	NA

Vay-2	STIC EXPERT HIT Negative<br>ID PaGIA H/PF4 ND<br>Hemosil AcuStar HIT-IgG Negative<br>Hemosil HIT-Ab Negative<br>PVS/PF4 Elisa OD: 1.97	NA	Standard SRA: POSITIVE<br>SRA-PF4: POSITIVE	NA
Vay-3	STIC EXPERT HIT Negative<br>ID PaGIA H/PF4 ND<br>Hemosil AcuStar HIT-IgG Negative<br>Hemosil HIT-Ab Negative<br>PVS/PF4 Elisa OD: 1.55	NA	Standard SRA: POSITIVE<br>SRA-PF4: POSITIVE	NA
Vay-4	STIC EXPERT HIT Negative<br>ID PaGIA H/PF4 ND<br>Hemosil AcuStar HIT-IgG Negative<br>Hemosil HIT-Ab ND<br>PVS/PF4 Elisa OD: 0.24	NA	Standard SRA: NEGATIVE<br>SRA-PF4: NEGATIVE	NA
Vay-5	STIC EXPERT HIT Negative<br>ID PaGIA H/PF4 Negative<br>Hemosil AcuStar HIT-IgG Negative<br>Hemosil HIT-Ab ND<br>PVS/PF4 Elisa OD: 1.44	NA	Standard SRA: NEGATIVE<br>SRA-PF4: POSITIVE	NA
Vay-6	STIC EXPERT HIT Negative<br>ID PaGIA H/PF4 Negative<br>Hemosil AcuStar HIT-IgG Negative<br>Hemosil HIT-Ab ND<br>PVS/PF4 Elisa OD: 1.52	NA	Standard SRA: POSITIVE<br>SRA-PF4: POSITIVE	NA
Vay-7	STIC EXPERT HIT Negative<br>ID PaGIA H/PF4 Negative<br>Hemosil AcuStar HIT-IgG Negative<br>Hemosil HIT-Ab ND<br>PVS/PF4 Elisa OD: 0.02	NA	Standard SRA: NEGATIVE<br>SRA-PF4: NEGATIVE	NA

Vay-8	STIC EXPERT HIT Negative<br>ID PaGIA H/PF4 Pos/Neg<br>Hemosil AcuStar HIT-IgG Negative<br>Hemosil HIT-Ab ND<br>PVS/PF4 Elisa OD: 0.74	NA	Standard SRA: NEGATIVE<br>SRA-PF4: POSITIVE	NA
Vay-9	STIC EXPERT HIT Negative<br>ID PaGIA H/PF4 ND<br>Hemosil AcuStar HIT-IgG Negative<br>Hemosil HIT-Ab ND<br>PVS/PF4 Elisa OD: 2.8	NA	Standard SRA: POSITIVE<br>SRA-PF4: POSITIVE	NA
Wan-1	Positive anti-PF4 antibodies<br>113.17 (normal _ 40) ng/ml.	NA	NA	NA
Wi-1	PF4/PVS ELISA IgG (OD) Ref <0.4; 2.8<br>PF4/PVS ELISA, Heparin inhibition (%) Ref <50; 99	NA	Heparin-induced multiple-electrode aggregometry (HIMEA) on a Multiplate analyzer (Dynabyte Medical) Positive	NA
Z-1	(PF4) IgG antibodies positive (2.59 optical density; normal <0.4)	NA	NA	NA
